# Magnetothermal Convection of Water with the Presence or Absence of a Magnetic Force Acting on the Susceptibility Gradient

**DOI:** 10.1371/journal.pone.0160090

**Published:** 2016-09-08

**Authors:** Syou Maki

**Affiliations:** Laboratory of Molecular Chemistry, Faculty of Pharmacy, Osaka Ohtani University, 3-11-1, Nishikiori-kita, Tondabayashi City, Osaka Pref. 584-8540, Japan; University of Glasgow, UNITED KINGDOM

## Abstract

Heat transfer of magnetothermal convection with the presence or absence of the magnetic force acting on the susceptibility gradient (*f*_sc_) was examined by three-dimensional numerical computations. Thermal convection of water enclosed in a shallow cylindrical vessel (diameter over vessel height = 6.0) with the Rayleigh-Benard model was adopted as the model, under the conditions of Prandtl number 6.0 and Ra number 7000, respectively. The momentum equations of convection were nondimensionalized, which involved the term of *f*_sc_ and the term of magnetic force acting on the magnetic field gradient (*f*_b_). All the computations resulted in axisymmetric steady rolls. The values of the averaged Nu, the averaged velocity components *U*, *V*, and *W*, and the isothermal distributions and flow patterns were almost completely the same, regardless of the presence or absence of the term of *f*_sc_. As a result, we found that the effect of *f*_sc_ was extremely small, although much previous research emphasized the effect with paramagnetic solutions under an unsteady state. The magnitude of *f*_sc_ depends not only on magnetic conditions (magnitudes of magnetic susceptibility and magnetic flux density), but also on the thermal properties of the solution (thermal conductivity, thermal diffusivity, and viscosity). Therefore the effect of *f*_b_ becomes dominant on the magnetothermal convection. Active control over the density gradient with temperature will be required to advance heat transfer with the effect of *f*_sc_.

## Nomenclature

*b*_*r*_ = radial component of magnetic flux density [T = Wb/m^2^ = V·s/m^2^]

*b*_*ϕ*_ = circumferential component of magnetic flux density [T = Wb/m^2^ = V·s/m^2^]

*b*_*z*_ = vertical component of magnetic flux density [T = Wb/m^2^ = V·s/m^2^]

$\vec{b}$ = magnetic flux density vector; $\vec{b}\;=\;(b_{r},b_{\phi },b_{z})$

*B*_*R*_ = nondimensionalized radial component of magnetic flux density [–]

*B*_*θ*_ = nondimensionalized circumferential component of magnetic flux density [–]

*B*_*Z*_ = nondimensionalized vertical component of magnetic flux density [–]

*$\vec{B}$* = nondimensionalized magnetic flux density vector; $\vec{B}\;=\;(B_{R},B_{\theta },B_{Z})$

*b*_*a*_ = representative magnetic flux density; *b*_*a*_ = μ_0_ *i* /*h*_*z*_ [T]

*f*_b_ = magnetic force acting on the magnetic field gradient [N/m^3^]

*f*_sc_ = magnetic force acting on the susceptibility gradient [N/m^3^]

*f*_*mR*_ = radial component of magnetic force [N/m^3^]

*f*_*mθ*_ = circumferential component of magnetic force [N/m^3^]

*f*_*mZ*_ = vertical component of magnetic force [N/m^3^]

$\vec{f_{m}}$ = magnetic force vector; $\vec{f_{m}}\;=\;(f_{mR},f_{m}{{}_{\theta }}_{,}f_{mZ})$

*F*_*mR*_ = nondimensionalized radial component of magnetic force [–]

*F*_*mθ*_ = nondimensionalized circumferential component of magnetic force [–]

*F*_*mZ*_ = nondimensionalized vertical component of magnetic force [–]

$\vec{F_{m}}$ = nondimensionalized magnetic force vector; $\vec{F_{m}}\;=\;(F_{mR},F_{m}{{}_{\theta }}_{,}F_{mZ})$

Gr = Grashof number; $Gr=\frac{g\;\beta_{0}\;\left(\Theta_{hot}-\Theta_{cold}\right)\;h_{z}^{3}}{\nu^{2}}$ [–]

$\vec{g}$ = gravitational vector; $\vec{g}\;=\;(0,\;0,\;-\text{g})$

g = gravitational acceleration (9.807) [m/s^2^]

*h*_*z*_ = height of vessel, standard length for the nondimensionalization [m]

*i* = electric current in a coil [A]

Nu = Nusselt number [–]

**Fig 1:**
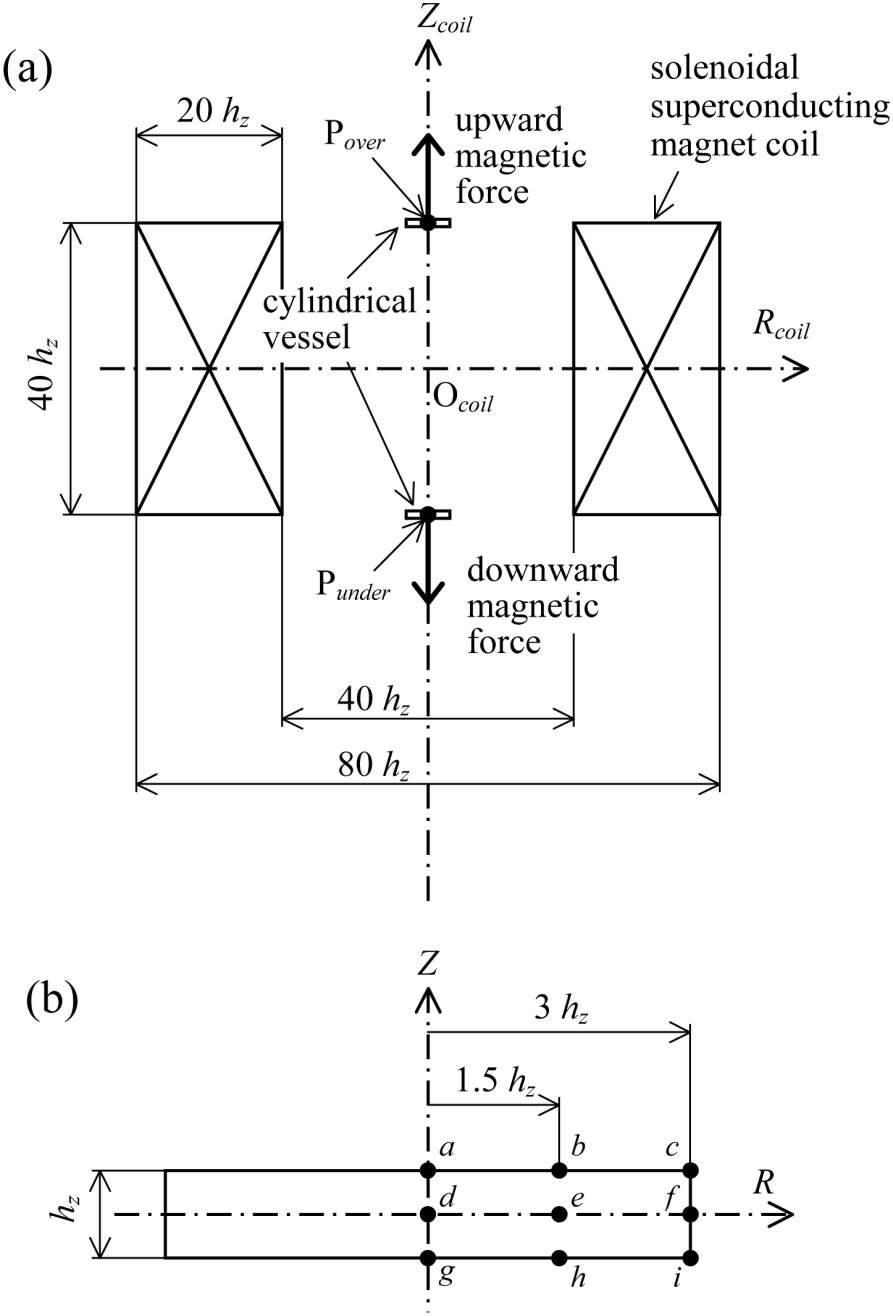
(a) is a schematic illustration of the positional relationship between the cylindrical vessel and the solenoidal superconducting magnet coil. In the bore of the magnet, the vertical component of magnetic force is symmetrical about the coil center (see bold arrows). The magnitude of $\text{grad}({\vec{b}}^{2})$ becomes largest around the representative points of P_*over*_ and P_*under*_, and its direction is oriented to the vertical. Consequently, the effect of gravity can be most efficiently controlled by the magnetic force. (b) the representative points *a–i* marked on the vertical cross-section of the cylindrical vessel.

O_*coil*_ = Origin of the cylindrical coordinate system on the magnet coil (Fig 1)

**Fig 2:**
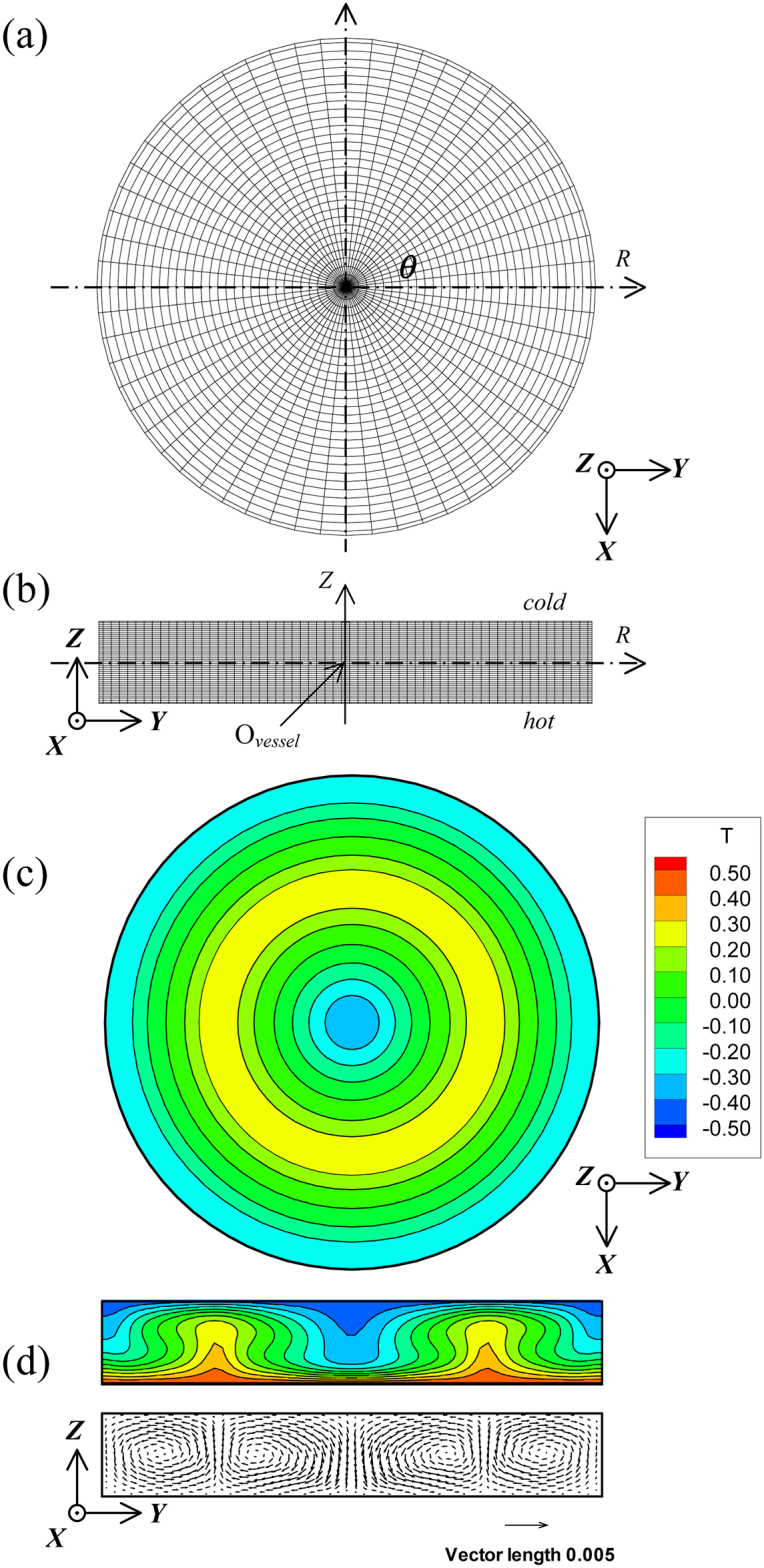
(a) and (b) show the horizontal and vertical cross-sections of the computation meshes used in this study. (c) and (d) show the three-dimensional numerical computations of Rayleigh-Benard convection at Pr = 6.0 and Ra = 7000. (c) is the isothermal distribution cross-sectioned on the *Z* = 0 plane. (d) is the isothermal and velocity distributions cross-sectioned on the *θ* = 0 plane.

O_*vessel*_ = Origin of the cylindrical coordinate system on the liquid vessel at its center (Fig 2)

*p* = pressure [N/m^2^]

*P* = nondimensionalized pressure [–]

*p*_0_ = pressure at *Θ*0 [N/m^2^]

*p*’ = perturbation term of pressure [N/m^2^]

P_*over*_ = representative point located on the *z* axis in the vicinity of the upper coil edge

P_*under*_ = representative point located on the *z* axis in the vicinity of the lower coil edge

Pr = Prandtl number; Pr = / [–]

*r* = radial component of the cylindrical coordinate system on the vessel to carry out the computation of convection [m]

*R* = nondimensionalized radial component of the cylindrical coordinate system on the vessel to carry out the computation of convection [–]

*R*_*coil*_ = nondimensionalized radial component on the cylindrical coordinate system defined at the center of the magnet coil [–]

Ra = Rayleigh number; $\text{Ra}=\frac{g\;\beta_{0}\;\left(\Theta_{hot}-\Theta_{cold}\right)\;h_{z}^{3}}{\alpha \;\nu }$ [–]

Ra_m_ = magnetic Rayleigh number; ${\text{Ra}}_{\text{m}}\;=\text{ Ra}\cdot \;\left(1+\frac{\gamma }{2}\cdot \frac{\partial \;(B^{2})}{\partial \;Z}\right)$ [–]

*t* = time [s]

*T* = nondimensionalized temperature [–]

*u* = radial velocity component [m/s]

$\vec{u}$ = velocity vector; $\vec{u}\;=\;\left(u,v,w\right)$

*U* = nondimensionalized radial velocity component [–]

$\vec{U}$ = nondimensionalized velocity vector; $\vec{U}\;=\;\left(U,V,W\right)$

*v* = circumferential velocity component [m/s]

vel_max_ = actual maximum velocity [m/s]

*V* = nondimensionalized circumferential velocity component [–]

Vel_max_ = nondimensionalized maximum velocity [–]

*w* = axial velocity component [m/s]

*W* = nondimensionalized axial velocity component [–]

*X* = nondimensionalized *x*-direction of Cartesian coordinates system defined on the center of the magnet coil O_*coil*_ [–]

*Y* = nondimensionalized *y*-direction of Cartesian coordinates system defined on the center of the magnet coil O_*coil*_ [–]

*z* = axial component [m]

*Z* = nondimensionalized axial component [–]

*Z*_*coil*_ = nondimensionalized axial component on the coordinate system defined on the center of the magnet coil O_*coil*_ [–]

### Greek letters

α = thermal diffusivity of solution [m^2^/s]

β = volumetric coefficient of expansion of fluid [1/K]

β_0_ = volumetric coefficient of expansion of fluid at *Θ*0 [1/K]

γ = nondimensional parameter representing the magnitude of the magnetic force; $\gamma \;=\;\frac{\chi_{0}b_{0}{}^{2}}{\mu_{0}\;g\;h_{z}}$ [–]

*θ* = angular coordinate for nondimensionalized equation [rad]

*Θ*_*hot*_ = hot surface temperature [K]

*Θ*_*cold*_ = cold surface temperature [K]

*Θ*0 = representative temperature [K]

μ = coefficient of viscosity [Pa·s]

μ_0_ = magnetic permeability of vacuum [H/m]

ν = kinematic viscosity [m^2^/s]

ρ(*Θ*) = density of solution, this is a function of temperature [kg/m^3^]

ρ_0_ = density of solution at *Θ*0 [kg/m^3^]

ρ_∞_ = density of solution sufficiently away from the (*Θ*) [kg/m^3^]

*τ* = nondimensionalized time [–]

*ϕ* = angular coordinate [rad]

χ_m_ = mass magnetic susceptibility [m^3^/kg]

χ_0_ = volumetric magnetic susceptibility at *Θ*0 [–]

χ_v_ = volumetric magnetic susceptibility [–]

∇^2^ = differential operator (Laplacian)

## Introduction

Magnetic force, a body force, was characterized by M. Faraday in 1847 [1]. In order to utilize the magnetic force as a driving force in heat and mass transfer, an extremely large magnetic flux density is necessary, since the magnetic susceptibility of a diamagnetic substance, even that of paramagnetic materials, is very small. Because generating such a large magnetic flux was difficult at that time, there was hardly any research on magnetic force until the control over thermal convection with a magnetic force was published in 1991 by Braithwaite, *et al*. [2,3]. Owing to the practical progress of a helium-free superconducting magnet, which makes it possible to generate a strong and stable magnetic field for a long time, many kinds of studies related to magnetic force rapidly spread into a variety of fields in the latter half of the 1990s. At present, technical applications of magnetic force, such as heat and mass transfer [4–9] and magnetic separation [10–16] are being explored in new fields of engineering, as well as in the fields of biochemistry [17–21], crystal growth [22–28], and other magneto-sciences [29–35].

Generally, two different forces, i.e., $\frac{\chi_{\text{v}}}{2\mu_{0}}\text{grad}{\left(\vec{b}\right)}^{2}$ and $\frac{{\left(\vec{b}\right)}^{2}}{2\mu_{0}}\text{grad}(\chi_{\text{v}})$, are known as the magnetic force. They are derived by the gradient of the magnetic energy, as shown in Ref. [36]. Further details are also given in Ref [37]. The force of $\frac{\chi_{\text{v}}}{2\mu_{0}}\text{grad}{\left(\vec{b}\right)}^{2}$ is well known as a conventional magnetic force. On the other hand, the force of $\frac{{\left(\vec{b}\right)}^{2}}{2\mu_{0}}\text{grad}(\chi_{\text{v}})$ is known as “(magnetic) concentration gradient force” [36, 38–40], “paramagnetic (gradient) force” [41–43], or “concentration gradient paramagnetic force” [44]. In this paper, the force of $\frac{\chi_{\text{v}}}{2\mu_{0}}\text{grad}{\left(\vec{b}\right)}^{2}$ is called “a magnetic force acting on the magnetic field gradient (*f*_b_)”, and the force of $\frac{{\left(\vec{b}\right)}^{2}}{2\mu_{0}}\text{grad}(\chi_{\text{v}})$ is called “a magnetic force acting on the susceptibility gradient (*f*_sc_)”. Most studies using *f*_sc_ are associated with research on the magnetic effect on diffusive convection [28, 36–47]. On the other hand, most studies on magnetothermal convection [3–6,8,9] have been conducted with the force of *f*_b_ only. The difference in the impact of these forces has been studied in the field of diffusive convection [38, 39, 46], but it is virtually unknown in the field of thermal convection. This is one of the motives of this study.

The volumetric magnetic susceptibility χ_v_ is a nondimensional property expressed by the product of mass magnetic susceptibility χ_m_ and density ρ. Where there is a local specific change in density due to temperature difference or the like, nonuniformity in a magnetic force occurs, even if $\text{grad}({\vec{b}}^{2})$ stays constant. On the one hand, conventional thermal convection, i.e. Rayleigh-Benard convection, is induced by the nonuniformity in the medium due to the local temperature differences, the driving force of which is attributable to the gravitational force. Gravitational force is a body force, as is magnetic force, hence the driving mechanisms in Rayleigh-Benard convection and magnetothermal convection have many features in common, except for one great difference between the two: the direction of the driving force in magnetic force is dependent on that of $\text{grad}({\vec{b}}^{2})$, while the driving force in Rayleigh-Benard convection directs only vertically. Ozoe, *et al*. focused on the common features of the driving forces in magnetothermal convection and Rayleigh-Benard convection, and nondimensionalized the momentum equation where both the Boussinesq term and the magnetic force term of *f*_b_ were joined together [5,6]. The newly introduced nondimensional parameter is the magnetic Rayleigh number, Ra_m_. The method by Ozoe, *et al*. is often utilized in numerical computations of magnetothermal convection [8, 34].

In this study, the effect of *f*_sc_ on convection was numerically examined. Nobody knows how much influence the presence or absence of the term of *f*_sc_ has on the isothermal distributions and flow patterns. In Discussion, the effect of *f*_sc_ was verified with actual magnet size and thermal properties.

## Equations

In many cases, the magnetothermal convection was approximated by the following momentum equation.

$$\rho_{0}\frac{D\vec{u}}{Dt}=-\text{grad}(p)+\mu \;\nabla^{2}\;\vec{u}+(\rho (\Theta )\;-\rho_{\infty })\;\vec{g}+\frac{\;\chi_{\text{v}}}{2\mu_{0}}\text{grad}(\;{\vec{b}}^{2})$$ (1)

The first term in the right-hand side denotes pressure. The second term denotes viscosity, and the third term is buoyancy by Boussinesq approximation. The fourth term corresponds to the term of *f*_b_.

In this study, the following equation was considered as the momentum equation of magnetothermal convection.

$$\rho_{0}\frac{D\vec{u}}{Dt}=-\text{grad}(p)+\mu \nabla^{2}\vec{u}+(\rho (\Theta )\;-\rho_{\infty })\;\vec{g}+\frac{\;\chi_{\text{v}}}{2\mu_{0}}\text{grad}(\;{\vec{b}}^{2})+\frac{\;{\left(\vec{b}\right)}^{2}}{2\mu_{0}}\text{grad}(\chi_{\text{v}})$$ (2)

The first to fourth terms on the right-hand side are the same as those in Eq 1. The newly added fifth term corresponds to the term of *f*_sc_. A similar expression is presented in Ref [31]. Eq 2 can be arranged below.

$$\rho_{0}\frac{D\vec{u}}{Dt}=-\text{grad}(p)+\mu \nabla^{2}\vec{u}+(\rho (\Theta )\;-\rho_{\infty })\;\vec{g}+\frac{\;1}{2\mu_{0}}\text{grad}[\chi_{\text{v}}\;{\left(\vec{b}\right)}^{2}]$$ (3)

In the process of the nondimensionalization of Eq 3, we advanced the Ozoe and Tagawa approach [5,6]. We also adopted the Hellums and Churchill method [48]. As shown in Appendix A, we succeeded in the nondimensionalization of Eq 3. In the bore of a solenoidal subperconducting magnet, we know the magnetic force distributes axisymmetrically. Thereby the momentum equation was expressed with the cylindrical coordinate system (*R*, *θ*, *Z*) as given in Eq 4 below. Here, *B*^2^ = *B*_*R*_^2^ + *B*_*θ*_^2^ + *B*_*Z*_^2^.

$$\begin{array}{l} \frac{\partial \;U}{\partial \;\tau }+U\frac{\partial \;U}{\partial \;R}+\frac{V}{R}\frac{\partial \;U}{\partial \theta }-\frac{V^{2}}{R}+W\frac{\partial \;U}{\partial \;Z}=-\frac{\partial \;P}{\partial \;R}\;\\ \;\;+\text{Pr }\left[\frac{\partial }{\partial \;R}\left\{\frac{1}{R}\cdot \frac{\partial \;(R\cdot U)}{\partial \;R}\right\}+\frac{1}{R^{2}}\frac{\partial^{2}U}{\partial \theta^{2}}-\frac{2}{R^{2}}\frac{\partial \;V}{\partial \theta }+\frac{\partial^{2}U}{\partial \;Z^{2}}\right]-\frac{\gamma }{2}\cdot \text{Pr}\cdot \text{Ra}\cdot \;\frac{\partial \;(T\cdot \;B^{2})}{\partial \;R} \end{array}$$ (4a)

$$\begin{array}{l} \frac{\partial \;V}{\partial \;\tau }+U\frac{\partial \;V}{\partial \;R}+\frac{V}{R}\frac{\partial \;V}{\partial \theta }+\frac{U\cdot V}{R}+W\frac{\partial \;V}{\partial \;Z}=-\frac{1}{R}\frac{\partial \;P}{\partial \;\theta }\;\\ \;\;+\text{Pr}\left[\frac{\partial }{\partial \;R}\left\{\frac{1}{R}\cdot \frac{\partial \;(R\cdot V)}{\partial \;R}\right\}+\frac{1}{R^{2}}\frac{\partial^{2}V}{\partial \theta^{2}}+\frac{2}{R^{2}}\frac{\partial \;U}{\partial \theta }+\frac{\partial^{2}V}{\partial \;Z^{2}}\right]-\frac{\gamma }{2}\cdot \text{Pr}\cdot \text{Ra}\cdot \frac{1}{R}\frac{\partial \;(T\cdot \;B^{2})}{\partial \;\theta } \end{array}$$ (4b)

$$\begin{array}{l} \frac{\partial \;W}{\partial \;\tau }+U\frac{\partial \;W}{\partial \;R}+\frac{V}{R}\frac{\partial \;W}{\partial \theta }+W\frac{\partial \;W}{\partial \;Z}=-\frac{\partial \;P}{\partial \;Z}\;\\ \;\;+\text{Pr }\left[\frac{1}{R}\frac{\partial }{\partial \;R}\left\{R\cdot \frac{\partial \;W}{\partial \;R}\right\}+\frac{1}{R^{2}}\frac{\partial^{2}W}{\partial \theta^{2}}+\frac{\partial^{2}W}{\partial \;Z^{2}}\right]-\text{Pr}\cdot \text{Ra}\cdot \;T\;-\frac{\gamma }{2}\cdot \text{Pr}\cdot \text{Ra}\cdot \frac{\partial \;(T\cdot \;B^{2})}{\partial \;Z} \end{array}$$ (4c)

The nondimensionalization processes to introduce Eqs 4a–4c from Eq 3 are described in Appendix A.

Eq 4c is expanded as follows.

$$\begin{array}{l} \frac{\partial \;W}{\partial \;\tau }+U\frac{\partial \;W}{\partial \;R}+\frac{V}{R}\frac{\partial \;W}{\partial \theta }+W\frac{\partial \;W}{\partial \;Z}=-\frac{\partial \;P}{\partial \;Z}+Pr\;\left[\frac{1}{R}\frac{\partial }{\partial \;R}\left\{R\cdot \frac{\partial \;W}{\partial \;R}\right\}+\frac{1}{R^{2}}\frac{\partial^{2}W}{\partial \theta^{2}}+\frac{\partial^{2}W}{\partial \;Z^{2}}\right]\\ \;\;\;\;-\text{Pr}\cdot \text{Ra}\cdot \;T-\frac{\gamma }{2}\cdot \text{Pr}\cdot \text{Ra}\cdot \;T\cdot \frac{\partial \;(B^{2})}{\partial \;Z}-\frac{\gamma }{2}\cdot \text{Pr}\cdot \text{Ra}\cdot B^{2}\frac{\partial \;T}{\partial \;Z} \end{array}$$ (4d)

The fourth term in the right-hand side is the nondimensionalized *f*_b_, and the fifth term is the nondimensionalized *f*_sc_.

By the use of Ra_m_, Eq 4d is presented as follows.

$$\begin{array}{l} \frac{\partial \;W}{\partial \;\tau }+U\frac{\partial \;W}{\partial \;R}+\frac{V}{R}\frac{\partial \;W}{\partial \theta }+W\frac{\partial \;W}{\partial \;Z}=-\frac{\partial \;P}{\partial \;Z}+Pr\;\left[\frac{1}{R}\frac{\partial }{\partial \;R}\left\{R\cdot \frac{\partial \;W}{\partial \;R}\right\}+\frac{1}{R^{2}}\frac{\partial^{2}W}{\partial \theta^{2}}+\frac{\partial^{2}W}{\partial \;Z^{2}}\right]\\ \;\;\;\;-\text{Pr}\cdot {\text{Ra}}_{\text{m}}\cdot \;T-\frac{\gamma }{2}\cdot \text{Pr}\cdot \text{Ra}\cdot B^{2}\frac{\partial \;T}{\partial \;Z} \end{array}$$ (4e)

When we ignored the effect of *f*_sc_, the *z*-directional component of magnetic force is presented as follows.

$$\frac{\partial \;W}{\partial \;\tau }+U\frac{\partial \;W}{\partial \;R}+\frac{V}{R}\frac{\partial \;W}{\partial \theta }+W\frac{\partial \;W}{\partial \;Z}=-\frac{\partial \;P}{\partial \;Z}+Pr\left[\frac{1}{R}\frac{\partial }{\partial \;R}\left\{R\cdot \frac{\partial \;W}{\partial \;R}\right\}+\frac{1}{R^{2}}\frac{\partial^{2}W}{\partial \theta^{2}}+\frac{\partial^{2}W}{\partial \;Z^{2}}\right]-\text{Pr}\cdot {\text{Ra}}_{\text{m}}\cdot \;T$$ (4f)

To conduct the three-dimensional numerical computation, the equation of continuity (Eq 5) and the energy equation (Eq 6), as presented below, are indispensable.

$$\text{div }\vec{u}=0$$ (5)

$$\frac{D\Theta }{Dt}=\alpha \;\nabla^{2}\;\Theta$$ (6)

In a way similar to that of Hellums and Churchill [48], Eqs 5 and 6 were nondimensionalized as shown below.

$$\frac{1}{R}\frac{\partial }{\partial \;R}(R\cdot U)+\frac{1}{R}\frac{\partial \;V}{\partial \phi }+\frac{\partial \;W}{\partial \;Z}=0$$ (7)

$$\frac{\partial \;Τ}{\partial \;\tau }+U\frac{\partial \;Τ}{\partial \;R}+\frac{V}{R}\frac{\partial \;Τ}{\partial \theta }+W\frac{\partial \;Τ}{\partial \;Z}=\frac{1}{R}\frac{\partial }{\partial \;R}(R\cdot \frac{\partial Τ}{\partial \;R})+\frac{1}{R^{2}}\frac{\partial^{2}Τ}{\partial \theta^{2}}+\frac{\partial^{2}Τ}{\partial \;Z^{2}}$$ (8)

Finally, five unknown numbers of velocity, *U*, *V*, and *W*, temperature *T*, and pressure *P*, were analytically solved by using the five Eqs 4a, 4b, 4e, 7 and 8.

In this study, other computations using the five eqs 4a, 4b, 4f, 7 and 8 were independently conducted as described in the last paragraph in Introduction.

## Models

In this study, thermal convection in the Rayleigh-Benard model was used for a comparison between new types of magnetothermal convection with the terms of *f*_b_ and *f*_sc_ (e.g., Eq 3) and the conventional magnetothermal convection with *f*_b_ only (e.g., Eq 1). We used a cylindrical vessel where the aspect ratio (diameter/height) was 6.0. For the conditions of velocity boundary, the top and bottom surfaces and the sidewall were solid. For the conditions of temperature boundary, the top surface was cooled, the bottom heated, and the sidewall adiabatic.

Fig 1(a) is a schematic illustration of the positional relationship between the cylindrical vessel and the solenoidal superconducting magnet coil. The distribution of magnetic field was numerically computed in accordance with an actual solenoidal superconducting magnet; i.e., the size of the solenoidal magnet corresponds to 200 mm in the inner diameter, 400 mm in the outer diameter, and 200 mm in height in the direction *z*. The magnet coil was approximated with a multi-layer coil where a single coil was uniformly arranged on the coil cross-section (100 mm in width and 200 mm in height) with 40 turns in the radial direction and 80 turns in the direction *z*, for 3,200 turns in all. The distribution of the magnetic field around the multi-layer coil was calculated by the superposition of all the magnetic field distributions established by each single coil. The magnet bore was orientated vertically and the inclination of the magnet was disregarded.

In the bore of the magnet, the magnetic field was nondimensionalized by a process similar to that of Ozoe, *et al* [5, 6]. The nondimensionalized magnetic force vector $\vec{F_{m}}\;=\;(F_{mR},F_{m\theta }{}_{,}F_{mZ})$ was defined by using the nondimensionalized magnetic field hereinbefore. The vertical component of magnetic force (*F*_*mZ*_) is symmetrical about the coil center. On the other hand, the radial component of magnetic force (*F*_*mR*_) directs axisymmetricaly, and the magnitude theoretically becomes zero as it approaches the axis *z*. For a diamagnetic substance like water, the directions of *F*_*mZ*_ and that of gravity are mutually reversed at the upper coil edge, and the effect of gravitational force is cancelled by the magnetic force, weakening the intensity of thermal convection. At the lower bore edge, the magnetic force enhances the magnitude of convection because the directions of *F*_*mZ*_ and gravitational force are equal. Hence in this study, a representative point located on the *z* axis in the vicinity of the upper coil edge (P_*over*_) and that of the lower coil edge (P_*under*_) were selected for the computations.

Fig 1(b) shows the representative points *a–i* marked on the vertical cross-section of the cylindrical vessel. The cylindrical vessel was horizontally located so that the vessel center (point *d* in Fig 1(b)) coincided with the P_*over*_ or the P_*under*_. The reason why the representative points of P_*over*_ and P_*under*_ were selected is that the solenoidal superconducting magnet has its largest $\text{grad}({\vec{b}}^{2})$ in the vicinity of the bore edge and, what is more, the direction of $\text{grad}({\vec{b}}^{2})$ is oriented to that of the direction of the bore axis. Consequently, the effect of *F*_*mR*_ is relieved, and the effect of gravity can be most efficiently controlled by the magnetic force *F*_*mZ*_. This simplification is useful for investigating the effect of *f*_sc_ on the heat transfer of convection.

## Computational Methodology

We utilized an equal-interval staggered mesh on the cylindrical coordinate system. We also used the Highly Simplify Marker and Cell method (HSMAC method) [49] and solved the equations by means of the explicit method. The averaged Nusselt number (Nu) was measured on the cooled surface by using the temperature gradient calculated on each spatially-weighted mesh. The velocity distributions along the center axis of the cylindrical vessel were computed by means of Ozoe and Toh’s approach [50].

The working fluid was assumed to be water at room temperature (26.5°C). Prandtl number (Pr) was set at 6.0. The effect of the magnetic force on water is worth examining for a number of studies of protein crystal growth [22,26] and magnetic separation [13,16]. In addition, we referred to Silveston’s results [51]. Silveston’s results represent the relationship between Ra and Nu on the double logarithmic chart. The most sensitive range of Ra to evaluate the effect of *f*_sc_ with the use of Nu is in the 5000 < Ra < 8000 range. In this study, Ra was fixed at 7000.

As regards the number of meshes for the numerical computation, a preliminary three-dimensional numerical computation of Rayleigh-Benard convection was carried out at Pr = 6.0 and Ra = 7000 by changing the number of meshes. The maximum number of meshes, where almost no change in the Nu number was found, was utilized, even though the number of meshes was large. All the results computed with different mesh sizes are shown in Table 1. Based on these results, we adopted the numbers 31, 61, and 41 in directions *R*, *θ*, and *Z*. Fig 2(a) and 2(b) show the horizontal and vertical cross-sections of the computation meshes used in this study. The horizontal cross-section passing through the center of the vessel was defined as the *Z* = 0 plane, and the height of the vessel was regarded as the standard length *h*_*z*_ (= 1.0) for the nondimensionalization. The condition for the nondimensional temperature at *Z* = ± 0.5 *h*_*z*_ was *T* = ∓0.5. Fig 2(c) and 2(d) show the temperature distribution on the *Z* = 0 plane and the isothermal and velocity distributions on the *θ* = 0 plane in Rayleigh-Benard convection at Pr = 6.0 and Ra = 7000 on the aforementioned computation meshes. This result was utilized as the common initial condition for all the computations of magnetothermal convection.

**Table 1:** Influence of the number of computational meshes. Pr = 6.0, Ra = 7000.

*R*	*θ*	*Z*	Nu
21	41	25	2.191
21	41	31	2.175
31	61	36	2.149
31*	61*	41*	2.145*
31	61	46	2.143

* The result of (*R*, *θ*, *Z*) = (31, 61, 41) was used as the initial condition in all the cases of computations of magnetothermal convection.

The magnitude of magnetic force was adjusted with the nondimensional parameter γ, which represents the intensity of magnetic force [6–8]. The value of γ was varied to −1.25471×10^−4^ and −6.27353×10^−5^. When γ is −1.25471×10^−4^, a pseudo-weightless condition is established at P_*over*_, and a strong hyper-gravity condition about twice that of gravity is simultaneously established at P_*under*_. When γ is −6.27353×10^−5^, a partial gravity condition about half that of gravity is established at P_*over*_, and a weak hyper-gravity condition of 1.5 times that of gravity is simultaneously established at P_*under*_. Table 2 show the magnitudes of *F*_*mR*_, *F*_*mZ*,_ and the resultant force between the *F*_*mZ*_ and gravity, measured at the typical points (*a ~ i*) on the vessel cross section shown in Fig 1(b) at P_*over*_ (*z* = 20 *h*_*z*_). Similarly, Table 3 summarized the magnitudes of *F*_*mR*_, *F*_*mZ*,_ and the resultant force, measured at the typical points in Fig 1(b) at P_*under*_ (*z* = 20 *h*_*z*_). Notice that the magnitude of nondimensionalized gravitational force is presented as 1. In the pseudo-weightless condition, the maximum vertical driving force (i.e., *F*_*mZ*_ + 1) was only 2.2% of gravity at point *i* (γ is −6.27353×10^−5^), and the maximum *F*_*mR*_ was only 4.9% of gravity at the same point *i*. Thus, the representative points of P_*over*_ and P_*under*_ are suitable for evaluating the effect of *f*_sc_.

**Table 2:** Radial and vertical components of the magnetic force calculated at representative points on the vessel at P_*over*_ (*z* = 20 *h*_*z*_).

γ	-1.25471×10^−4^			-6.27353×10^−5^		
	*F*_*mR*_	*F*_*mZ*_	*F*_*mZ*_ +1	*F*_*mR*_	*F*_*mZ*_	*F*_*mZ*_ +1
a*	0.000	-0.989	0.011	0.000	-0.494	0.506
*b*	-2.127 ×10^−2^	-0.992	0.008	-1.063 ×10^−2^	-0.496	0.504
*c*	-4.082 ×10^−2^	-1.001	0.001	-2.041 ×10^−2^	-0.501	0.499
*d*	0.000	-1.000	0.000	0.000	-0.500	0.500
*e*	-2.344 ×10^−2^	-1.004	-0.004	-1.172 ×10^−2^	-0.502	0.498
*f*	-4.500 ×10^−2^	-1.013	-0.013	-2.250 ×10^−2^	-0.506	0.494
*g*	0.000	-1.010	-0.010	0.000	-0.505	-0.495
*h*	-2.565 ×10^−2^	-1.013	-0.013	-1.282 ×10^−2^	-0.507	0.494
*i*	-4.926 ×10^−2^	-1.022	-0.022	-2.463 ×10^−2^	-0.511	0.489

* Representative points *a—i* are shown in Fig 1(B).

**Table 3:** Radial and vertical components of the magnetic force calculated at representative points on the vessel at P_*under*_ (*z* = 20 *h*_*z*_).

γ	-6.27353×10^−5^			-1.25471×10^−4^		
	*F*_*mR*_	*F*_*mZ*_	*F*_*mZ*_ +1	*F*_*mR*_	*F*_*mZ*_	*F*_*mZ*_ +1
a*	0.000	0.505	1.505	0.000	1.010	2.010
*b*	-1.282 ×10^−2^	0.507	1.507	-2.565 ×10^−2^	1.013	2.013
*c*	-2.463 ×10^−2^	0.511	1.511	-4.926 ×10^−2^	1.022	2.022
*d*	0.000	0.500	1.500	0.000	1.000	2.000
*e*	-1.172 ×10^−2^	0.502	1.502	-2.344 ×10^−2^	1.004	2.004
*f*	-2.250 ×10^−2^	0.506	1.506	-4.500 ×10^−2^	1.013	2.013
*g*	0.000	0.494	1.494	0.000	0.989	1.989
*h*	-1.063 ×10^−2^	0.496	1.496	-2.127 ×10^−2^	0.992	1.992
*i*	-2.041 ×10^−2^	0.501	1.501	-4.082 ×10^−2^	1.001	2.001

* Representative points *a—i* are shown in Fig 1(B).

In this study, magnetothermal convection with the terms of *f*_b_ and *f*_sc_ was labeled as cases A to D, while conventional magnetothermal convection, that is, the magnetic force term using *f*_b_ only, was labeled as cases E to H. The vessel center in cases A, B, E, and F was located at P_*over*_. The vessel center in cases C, D, G, and H was located at P_*under*_. The magnitude of γ was set at −1.25471×10^−4^ in cases A, D, E, and H, and was set at −6.27353×10^−5^ in cases B, C, F, and G.

## Results

**Fig 3:**
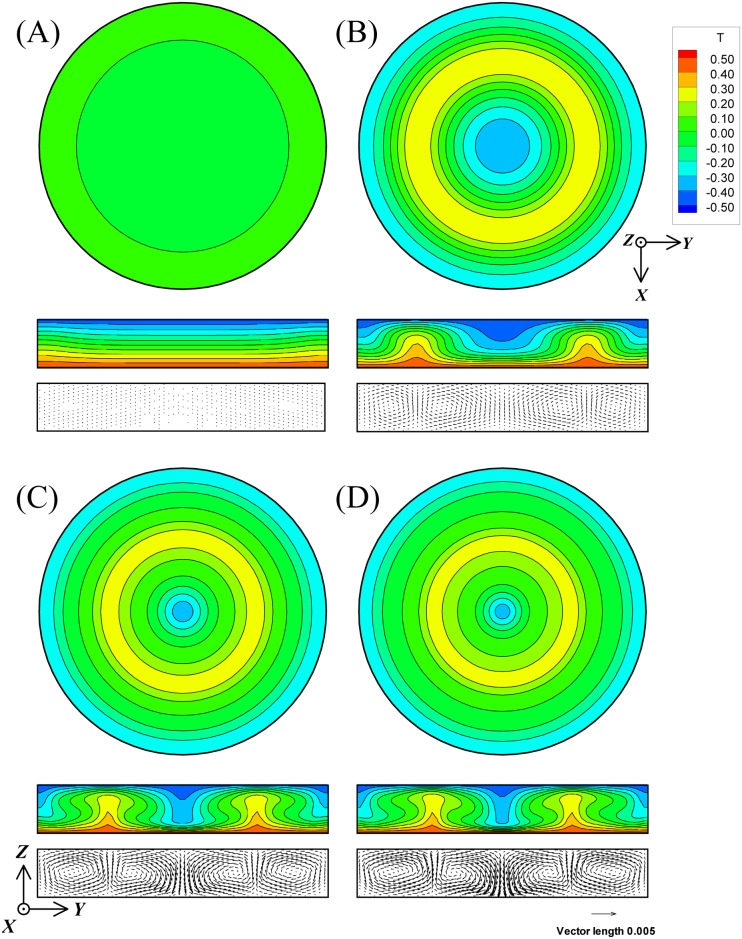
The isothermal and velocity distributions of magnetothermal convection in cases A, B, C, and D cross-sectioned with *Z* = 0 plane and *θ* = 0 plane. The figure numbers A to D correspond to each case. Pr and Ra are 6.0 and 7000, respectively. The magnitudes of γ were set at −1.25471×10^−4^ in cases A and D, and at −6.27353×10^−5^ in cases B and C.

**Fig 4:**
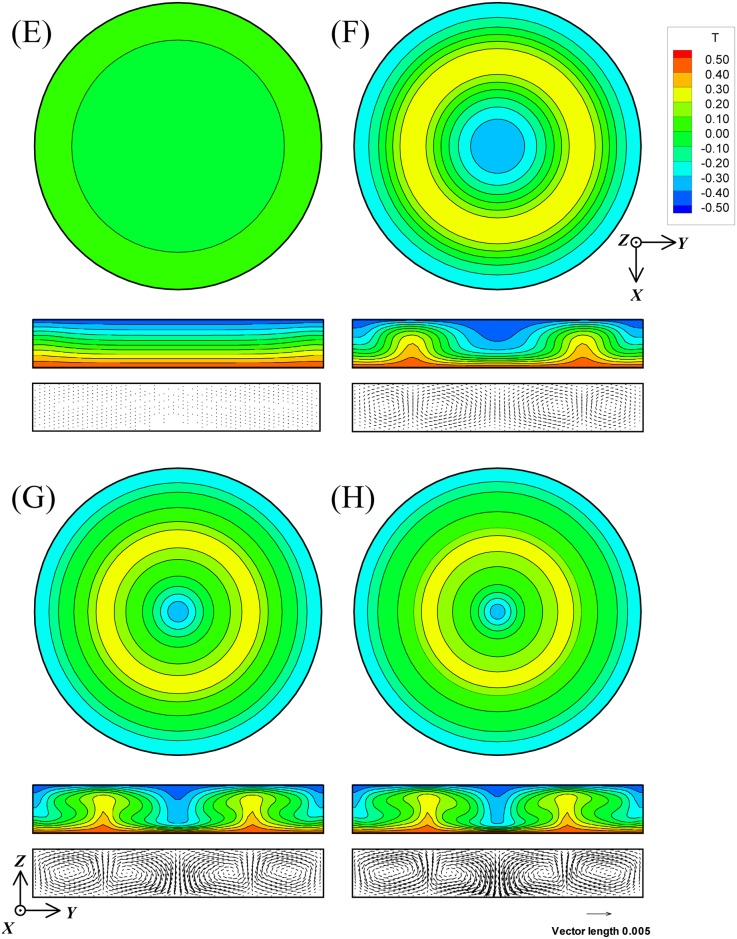
The isothermal and velocity distributions of magnetothermal convection in cases E, F, G, and H cross-sectioned with *Z* = 0 plane and *θ* = 0 plane. The figure numbers E to H correspond to each case. Pr and Ra are 6.0 and 7000, respectively. The magnitudes of γ were set at −1.25471×10^−4^ in cases E and H, and at −6.27353×10^−5^ in cases F and G. Ra_m_ of cases E to H corresponds to 0, 3500, 10500, and 14000, respectively.

**Fig 5:**
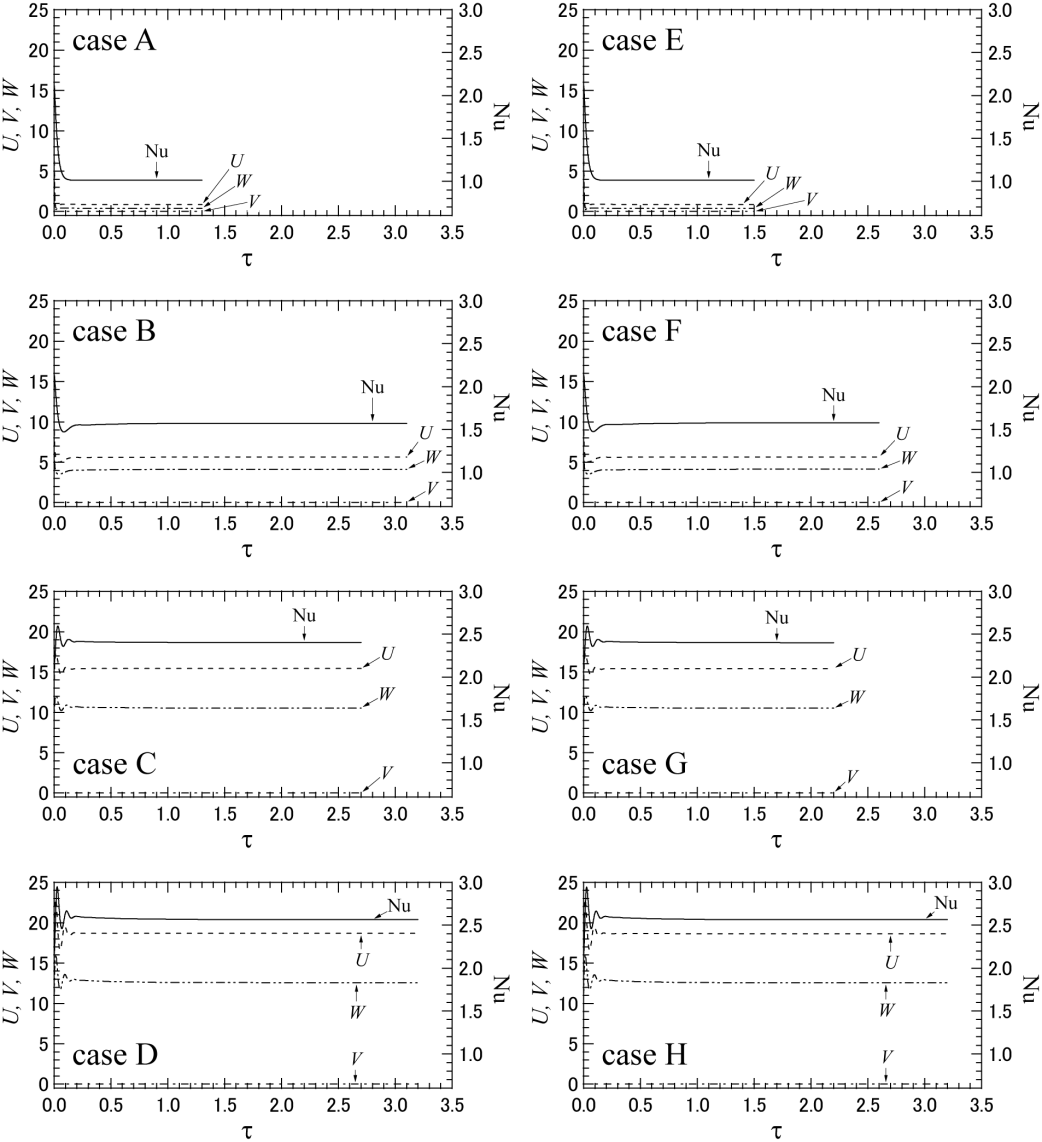
The transient response curves of velocity components *U*, *V*, and *W* and Nu in all cases. We can find that these curves were completely the same regardless of the presence or absence of the term of *f*_sc_.

Fig 3 shows the isothermal distributions on the *Z* = 0 cross-section in cases A to D, and also the isothermal and velocity distributions on the *θ* = 0 cross-section. Similarly, Fig 4 shows the isothermal and velocity distributions in cases E to H. The figure numbers A to H in Figs 3 and 4 correspond to cases A to H, respectively. Fig 5 shows the transient response curves of the velocity components *U*, *V*, and *W* and Nu in all cases. Table 4 summarizes the values of *U*, *V*, and *W* and Nu under the steady state in the Rayleigh-Benard convection at Pr = 6.0 and Ra = 7000 and the results of cases A to D. Table 5 summarizes the values of *U*, *V*, and *W* and Nu in the cases E to H. In these tables, the actual averaged velocity components *u*, *v*, and *w*, and the maximum velocities Vel_max_ and vel_max_ are also exhibited, considering the standard length *h*_*z*_ and thermal diffusivity α to be 0.005 m and 1.456×10^−7^ m^2^/s, respectively. Here, α is the thermal property of water at 26.5°C.

**Table 4:** Computational results of the magnetothermal convection with the terms of *f*_b_ and *f*_sc_. All the computations converged on a stable solution.

Case	Ra	Ra_m_	^1)^ *U*	^1)^ *V*	^1)^ *W*	Vel_max_	^3)^ Nu
			^2)^ (*u*) [mm/s]	^2)^ (*v*)	^2)^ (*w*)	^2)^ (vel_max_)	
	7000	7000	11.48	9.539×10^−3^	7.972	30.53	2.145
			(3.343×10^−1^)	(2.778×10^−4^)	(2.321×10^−1^)	(8.890×10^−1^)	
A	7000	0	8.799×10^−1^	2.237×10^−6^	3.952×10^−1^	1.690	1.011
			(2.562×10^−2^)	(6.514×10^−8^)	(1.151×10^−2^)	(4.921×10^−2^)	
B	7000	3500	5.629	4.381×10^−6^	4.130	12.62	1.573
			(1.639×10^−1^)	(1.276×10^−7^	(1.203×10^−1^)	(3.676×10^−1^)	
C	7000	10500	15.44	2.503×10^−6^	10.53	52.78	2.403
			(4.496×10^−1^)	(7.289×10^−8^)	(3.066×10^−1^)	1.537	
D	7000	14000	18.67	2.991×10^−6^	12.57	75.36	2.567
			(5.473×10^−1^)	(8.710×10^−1^)	(3.660×10^−1^)	(2.194)	

^1)^ *U*, *V*, and *W* are the averaged velocity components calculated under steady state, respectively.

^2)^ *u*, *v*, *w* and vel_max_ were actual velocities calculated by the method of Hellums and Churchill (see A19 in Appendix A). For example, *u* = *u*_0_*U* = (*α* / *h*_*z*_)*U* = 2.912×10^-5^*U* [m/s] = 2.912×10^-2^*U* [mm/s]. Here, α and *h*_*z*_ are 1.456×10^−7^ m^2^/s and 0.005 m, respectively.

^3)^ Nu is the averaged Nu calculated on the cold surface under steady state.

**Table 5:** Computational results of the magnetothermal convection with the term of *f*_b_ only. All the computations converged on a stable solution.

Case	Ra	Ra_m_	^1)^ *U*	^1)^ *V*	^1)^ *W*	Vel_max_	^3)^ Nu
			^2)^ (*u*) [mm/s]	^2)^ (*v*)	^2)^ (*w*)	^2)^ (vel_max_)	
E	7000	0	8.833×10^−1^	6.705×10^−6^	3.973×10^−1^	1.699	1.011
			(2.572×10^−2^)	(1.952×10^−7^)	(1.157×10^−2^)	(4.948×10^−2^)	
F	7000	3500	5.665	3.192×10^−5^	4.154	12.69	1.577
			(1.650×10^−1^)	(9.295×10^−7^)	(1.210×10^−1^)	(3.695×10^−1^)	
G	7000	10500	15.42	1.426×10^−3^	10.52	52.68	2.402
			(4.490×10^−1^)	(4.153×10^−5^)	(3.063×10^−1^)	(1.534)	
H	7000	14000	18.65	2.763×10^−3^	12.55	75.17	2.566
			(5.431×10^−1^)	(8.046×10^−5^)	(3.655×10^−1^)	(2.189)	

^1)^ *U*, *V*, and *W* are the averaged velocity components calculated under steady state, respectively.

^2)^ *u*, *v*, *w* and vel_max_ were actual velocities calculated by the same procedures as in Table 4.

^3)^ Nu was calculated by the same procedures as in Table 4.

As shown in Figs 3 and 4, every convection (cases A to H) resulted in axisymmetric steady rolls. Therefore, the circumferential velocity component *V* became nearly zero in the transient response curves in Fig 5.

As the effect of *f*_sc_, no differences were revealed in any of the comparisons of the isothermal and velocity distributions between case A and case E, case B and case F, case C and case G, and case D and case H. Furthermore, as shown in Tables 4 and 5, the averaged Nu and the *U*, *V*, and *W* coincided almost completely, with or without the term of *f*_sc_. In addition, as shown in Fig 5, the transient response curves were completely the same regardless of the presence or absence of the term of *f*_sc_. In summary, the computational results strengthen the fact that the effect of *f*_sc_ was extremely small.

## Discussions

### Verification of the effect of *f*_sc_

We investigated the effect of *f*_sc_ by using practical data of a magnetic field and thermal properties. When the thermal convection of water at 26.5°C is completely suppressed by an upward magnetic force, the intensity of the magnetic force should be almost equal to the gravitational force of water. This is calculated by the product of water density (996.6 kg/m^3^) and gravitational acceleration (9.807 m/s^2^), and is estimated as 9774 N. Hence a magnetic field condition of 1362 T^2^/m is necessary to completely suppress the thermal convection of water (See Appendix B).

With reference to the helium-free superconducting magnet (13T-100, JASTEC Co., Ltd) in the National Institute for Materials Science in Tsukuba, the maximum values of the vertical magnetic induction *b*_z_ and $b_{z}\frac{d\;b_{z}}{dz}$ are 13.00 T and 585.94 T^2^/m, respectively. Under such conditions, the magnetic induction at P_*over*_ and P_*under*_ becomes 9.16 T. If this magnet is to have the capability of generating a magnetic field condition of 1362 T^2^/m, the magnetic induction should be increased up to 19.82 T or more (See Appendix C), and the magnetic flux density at P_*over*_ and P_*under*_ should be 13.97 T (See Appendix D).

On the other hand, in order to realize the Rayleigh-Benard convection of Pr = 6.0 and Ra = 7000 in a cylindrical vessel of 0.005 m in height, the temperature difference between the cooled and heated surfaces should be modulated to 2.80°C (See Appendix E). This system is practicable when the cooled surface of the vessel is adjusted to 25.1°C and the heated one to 27.9°C. The density of water was estimated to be 996.9 kg/m^3^ at 25.1°C, and 996.2 kg/m^3^ at 27.9°C, respectively (the equation F1 and F2 in Appendix F). Then, the value of $\frac{d}{d\;z}\left(\;\rho (\Theta )\;\right)$ was deduced for 140 kg/m^3^ (the equation F3 in Appendix F). Finally, when the vessel was located at P_*over*_ and P_*under*_ and the magnetic field condition of 1362 T^2^/m was applied, the value of *f*_sc_, i.e., $\frac{{\left(\vec{b}\right)}^{2}\chi_{\text{m}}}{2\mu_{0}}\frac{d}{d\;z}\left(\;\rho (\Theta )\;\right)$, could be estimated as −98.39 N (the equation F4 in Appendix F). This value was only a 1.007% contribution as compared to the value of 9774 N.

If a thermal convection with the same magnitude mentioned above (Ra = 7000) is realized in a half-sized cylindrical vessel (*h*_z_ = 0.0025 m and Ra = 7000), the temperature difference between the hot and cold surfaces should increase to eight times larger than that of the present case (since Ra is proportional to the cube of *h*_z_). This causes an increase in the temperature gradient and leads to the enhancement of grad(χ_v_). On the other hand, the distance between the vessel and the magnet coil is doubled in the nondimensinalized space. According to the Biot-Savart law, the magnetic induction is inversely proportional to the square of distance. Therefore, the magnitude of $\vec{b}$ becomes a quarter-magnitude, and hence the magnitude of ${\left(\vec{b}\right)}^{2}$ becomes a sixteenth part. The temperature gradient in the half-sized cylindrical vessel equilibrates by doubling that of the initial vessel to correspond to the same interval in the computational grid. In summary, if the temperature gradient is linearly approximated as calculated in the previous paragraph, the magnitude of $\frac{{\left(\vec{b}\right)}^{2}}{2\mu_{0}}\text{grad}(\chi_{\text{v}})$ is constant, regardless of the vessel size. Through the above verifications, there is no doubt that the effect of *f*_sc_ on convection becomes negligibly small.

The effect of *f*_sc_ has been emphasized in many previous studies [28, 36, 38–47]. These studies are related to the unsteady mass transfer with paramagnetic solutions when a locally large grad(χ_v_) was spontaneously realized. In contrast, the present study evaluated the effect of *f*_sc_ with a diamagnetic solution under steady conditions. Then, inducement of large grad(χ_v_) is suppressed due to thermal diffusion of water. Consequently the effect of *f*_sc_ was changed substantially negligible, compared with the effect of *f*_b_. In other words, the effect of *f*_sc_ on convection depends not only on magnetic conditions χ and $\vec{b}$, but also thermal properties of the fluid, e.g. thermal conductivity, thermal diffusivity, and viscosity.

### Magnetothermal convection and Rayleigh-Benard convection

The difference between magnetothermal convection and Rayleigh-Benard convection was examined with Ra and Ra_m_ being equal. Four types of Rayleigh-Benard convection, Pr = 6.0 and Ra = 0, 3500, 10500, and 14000, were independently computed with the same mesh numbers and computational method, and they were labeled as cases I, J, K, and L, respectively.

**Fig 6:**
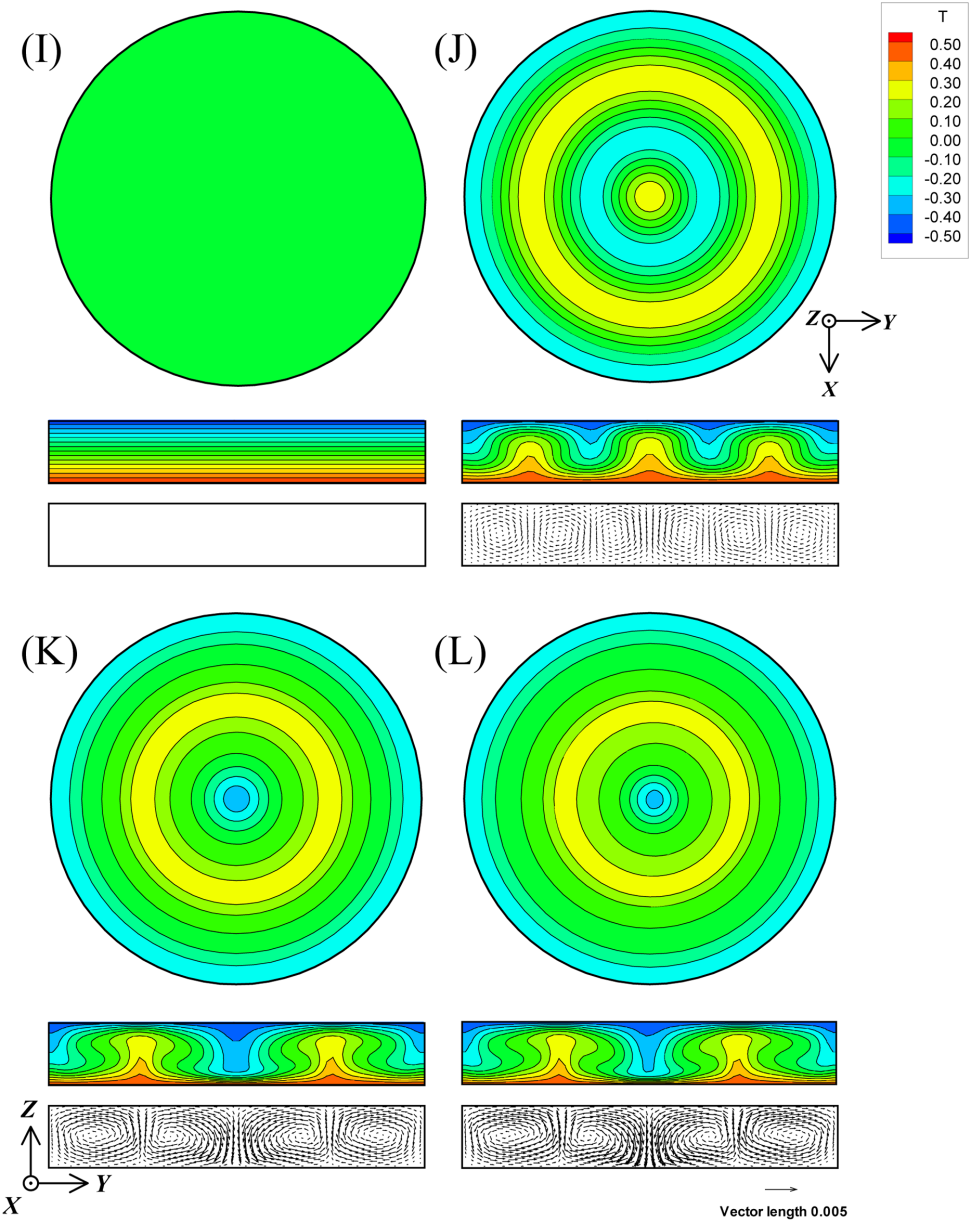
The isothermal and velocity distributions of Rayleigh-Benard convection in cases I, J, K, and L. The figure numbers correspond to each case. Pr is 6.0. Ra of cases I to K is 0, 3500, 10500, and 14000, respectively.

Fig 6 shows the isothermal and velocity distributions in cases I to L. Table 6 shows the averaged Nu and the averaged velocities of *U*, *V*, and *W* in these cases. The actual averaged velocity components of *U*, *V*, and *W*, and the maximum velocities Vel_max_ are also exhibited.

**Table 6:** Computational results of four types of Rayleigh-Benard convection. All the computations converged on a stable solution.

Case	Ra	^1)^ *U*	^1)^ *V*	^1)^ *W*	Vel_max_	^3)^ Nu
		^2)^ (*u*) [mm/s]	^2)^ (*v*)	^2)^ (*w*)	^2)^ (vel_max_)	
I	0	0.000	0.000	0.000	0.000	1.000
		(0.000)	(0.000)	(0.000)	(0.000)	
J	3500	5.490	1.224×10^−3^	5.123	21.59	1.668
		(1.599×10^−1^)	(3.564×10^−5^)	(1.492×10^−1^)	(6.288×10^−1^)	
K	10500	15.48	4.789×10^−2^	10.53	53.14	2.407
		(4.508×10^−1^)	(1.395×10^−3^)	(3.066×10^−1^)	(1.548)	
L	14000	18.73	7.842×10^−1^	12.56	76.48	2.574
		(5.454×10^−1^)	(2.284×10^−2^)	(3.657×10^−1^)	(2.227)	

^1)^ *U*, *V*, and *W* are the averaged velocity components calculated under steady state, respectively.

^2)^ *u*, *v*, *w* and vel_max_ were actual velocities calculated by the same procedures as in Table 4.

^3)^ Nu was calculated by the same procedures as in Table 4.

As shown in Fig 6, all the convections resulted in axisymmetric steady rolls. The results of the flow patterns and heat transfer performance (see Table 6) were similar to those of the magnetothermal convections, provided that Ra and Ra_m_ were equal. This also suggests that the effects of magnetic force on convection depend on the magnitude of *f*_b_, not so much on the term of *f*_sc_.

## Conclusions

The effect of magnetic force acting on the susceptibility gradient (*f*_sc_) was examined by three-dimensional numerical computations, with thermal convection of water (diamagnetic substance) enclosed in a shallow cylindrical vessel of the Rayleigh-Benard model. We succeeded in nondimensionalizing the momentum equations of magnetothermal convection, which involved the term of *f*_sc_ and the term of the magnetic force acting on a magnetic field gradient (*f*_b_). As a result, the transient response curves of the averaged velocity components *U*, *V*, *W*, and Nu, and the isothermal distributions and the flow patterns (axisymmetric steady rolls) coincided almost completely, regardless of the presence or absence of the term of *f*_sc_. These results are different from those of previous reports, which considered unsteady phenomena with a paramagnetic solution. The effect of *f*_sc_ depends not only on the magnetic conditions of χ and $\vec{b}$, but also on the thermal properties of the fluid. When water is used as the working fluid, the inducement of a locally large grad(χ_v_) is suppressed more than in the case of paramagnetic solution. Therefore, the effect of *f*_b_ on the magnetothermal convection becomes dominant. Active control over the density gradient with temperature will be required to advance heat transfer with the effect of *f*_sc_,.

## Appendix A: Deduction of the momentum equation of Eq 3

The pressure perturbation at representative temperature *Θ* is assumed to be *p* = *p*_0_ + *p*’. In addition, it is assumed that density ρ and mass magnetic susceptibility χ_m_ of the solution are functions of temperature *Θ*, and that *ρ* = *ρ* (*Θ*), *χ*_m_ = *χ*_m_ (*Θ*). Eq 2 is changed into the equation below.

$$\rho_{0}\frac{D\vec{u}}{Dt}=-\text{grad}(p_{0}+p\prime )+\mu \nabla^{2}\vec{u}+(\rho (\Theta )\;-\rho_{\infty })\;\vec{g}+\frac{1}{2\;\mu_{0}}\text{grad}(\rho (\Theta )\;\chi_{\text{m}}(\Theta )\;b^{2})$$ (A1)

In Eq (A1) when *Θ* = *Θ*_0_, it is assumed that $\vec{u}\;=\;0$ at, *p*(*Θ*_0_) = *p*_0_, and *ρ*(*Θ*_0_) = *ρ*_0_. Hence the following equation is obtainable:

$$0=-\text{grad}(p_{0})+0+(\rho_{0}-\;\rho_{\infty })\;\vec{g}+\frac{1}{2\mu_{0}}\text{grad}(\rho_{0}\;\chi_{\text{m}}(\Theta_{0})\;b^{2})$$ (A2)

By the calculation of Eqs (A1) and (A2), the following relation is obtained.

$$\begin{array}{l} \rho_{0}\frac{D\vec{u}}{Dt}=-\text{grad}(p\prime )+\mu \nabla^{2}\vec{u}+(\rho (\Theta )-\rho_{0})\;\vec{g}\\ \;\;\;\;+\frac{1}{2\mu_{0}}\text{grad}\{(\rho (\Theta )\;\chi_{\text{m}}(\Theta )\;-\rho_{0}\;\chi_{\text{m}}(\Theta_{0})\;)b^{2}\} \end{array}$$ (A3)

*ρ*(*Θ*) and *χ*_m_ (*Θ*) are put into first order approximation by Taylor expansion.

$$\rho (\Theta )-\rho_{0}\approx {\left(\frac{\partial \rho (\Theta )}{\partial \Theta }\right)}_{0}(\Theta -\Theta_{0})\;$$ (A4a)

$$\chi_{\text{m}}(\Theta )-\chi_{\text{m}}(\Theta_{0})\approx {\left(\frac{\partial \chi_{\text{m}}(\Theta )}{\partial \Theta }\right)}_{0}(\Theta -\Theta_{0})\;$$ (A4b)

For the density, deformation of equation is performed with the use of coefficient of volumetric expansion β.

$$\beta =-\frac{1}{\rho (\Theta )}\frac{\partial \rho (\Theta )}{\partial \Theta }\;$$ (A5)

When *Θ* = *Θ*0:

$${\left(\frac{\partial \rho (\Theta )}{\partial \Theta }\right)}_{0}=-\beta_{0}\rho_{0}$$ (A6)

By substituting Eq (A6) for Eq (A4a),

$$\rho (\Theta )-\rho_{0}=-\rho_{0}\beta_{0}(\Theta -\Theta_{0})\;$$ (A7)

For a paramagnetic substance, Curie’s law [52] is applied to the magnetic susceptibility.

$$\chi_{\text{m}}(\Theta )=\frac{A}{\Theta }\;$$ (A8)

$$∴\;\frac{\partial \chi_{\text{m}}(\Theta )}{\partial \Theta }=-\frac{A}{\Theta^{2}}=-\frac{\chi_{\text{m}}(\Theta )}{\Theta }$$ (A9)

When *Θ* = *Θ*0:

$${\left(\frac{\partial \chi_{\text{m}}(\Theta )}{\partial \Theta }\right)}_{0}=-\frac{\chi_{0}}{\Theta_{0}}$$ (A10)

For a diamagnetic substance, temperature difference is very small in the magnetic susceptibility.

$${\left(\frac{\partial \chi_{\text{m}}(\Theta )}{\partial \Theta }\right)}_{0}=0$$ (A11)

By substituting Eq (A11) for Eq (A4b),

$$\chi_{\text{m}}(\Theta )-\chi_{\text{m}}(\Theta_{0})=0$$ (A12)

Similarly, *χ*_m_(*Θ*) *ρ*(*Θ*) is put into first order approximation by Taylor expansion.

$$\begin{array}{l} \rho (\Theta )\chi_{\text{m}}(\Theta )=\rho (\Theta_{0})\chi_{\text{m}}(\Theta_{0})+\frac{\partial (\rho (\Theta )\chi_{\text{m}}(\Theta ))}{\partial \Theta }{|}_{0}\;(\Theta -\Theta_{0})\;+\frac{1}{2}\frac{\partial^{2}(\rho (\Theta )\chi_{\text{m}}(\Theta ))}{\partial \Theta^{2}}{|}_{0}(\Theta -\Theta_{0}){\;}^{2}+\cdot \cdot \cdot \cdot \\ \;\approx \;\rho_{0}\chi_{0}+{\left[(\rho (\Theta )\frac{\partial \chi_{\text{m}}(\Theta )}{\partial \Theta })+(\chi_{\text{m}}(\Theta )\frac{\partial \rho (\Theta )}{\partial \Theta })\right]}_{0}(\Theta -\Theta_{0})\\ \;=\rho_{0}\chi_{0}+[\rho_{0}{\left(\frac{\partial \chi_{\text{m}}(\Theta )}{\partial \Theta }\right)}_{0}+\chi_{0}{\left(\frac{\partial \rho (\Theta )}{\partial \Theta }\right)}_{0}]\;(\Theta -\Theta_{0}) \end{array}$$ (A13)

Eqs (A6) and (A11) are substituted for Eq (A13), and the following equation is obtained.

$$\begin{array}{l} \rho (\Theta )\chi_{\text{m}}(\Theta )=\rho_{0}\chi_{0}+[\rho_{0}\;0+\chi_{0}(-\beta_{0}\rho_{0})]\;(\Theta -\Theta_{0})\\ \;\;\;\;=\rho_{0}\chi_{0}-\rho_{0}\chi_{0}\beta_{0}\;(\Theta -\Theta_{0}) \end{array}$$ (A14)

If the working fluid is paramagnetic, Eq (A13) is altered as follows, by using Eqs (A6) and (A10).

$$\rho (\Theta )\chi_{\text{m}}(\Theta )=\rho_{0}\chi_{0}-\rho_{0}\chi_{0}\beta_{0}\;(1+\frac{1}{\beta_{0}\Theta_{0}})\;(\Theta -\Theta_{0})$$ (A15)

Eqs (A7) and (A14) are substituted for Eq (A3).

$$\begin{array}{l} \rho_{0}\frac{D\vec{u}}{Dt}=-\text{grad }p\prime +\mu \;\nabla^{2}\;\vec{u}-\rho_{0}\beta_{0}(\Theta -\Theta_{0})\;\vec{g}\;\\ \;\;\;\;-\frac{1}{2\;\mu_{0}}\text{grad}[\rho_{0}\;\chi_{0}\beta_{0}\;(\Theta -\Theta_{0})\;{\left(\vec{b}\right)}^{2}] \end{array}$$ (A16)

Here, the relationship of ∇ *p* = ∇ *p’* is introduced, and the equation below is obtained.

$$\frac{D\vec{u}}{Dt}=-\frac{1}{\rho_{0}}\nabla \;p+\nu \;\nabla^{2}\;\vec{u}-\beta_{0}(\Theta -\Theta_{0})\;g\;{\left(0,0,1\right)}^{\text{T}}-\frac{\chi_{0}\beta_{0}}{2\;\mu_{0}}\text{grad}[\;(\Theta -\Theta_{0})\;{\left(\vec{b}\right)}^{2}]$$ (A17)

Component *r*:

$$\begin{array}{l} \frac{\partial \;u}{\partial \;t}+u\frac{\partial \;u}{\partial \;r}+\frac{v}{r}\frac{\partial \;u}{\partial \phi }-\frac{v^{2}}{r}+w\frac{\partial \;u}{\partial \;z}=-\frac{1}{\rho_{0}}\frac{\partial \;p}{\partial \;r}\;\\ \;\;\;\;\;\;+\nu \;[\frac{\partial }{\partial r}\{\frac{1}{r}\cdot \frac{\partial \;(r\cdot u)}{\partial r}\}+\frac{1}{r^{2}}\frac{\partial^{2}u}{\partial \phi^{2}}-\frac{2}{r^{2}}\frac{\partial \;v}{\partial \phi }+\frac{\partial^{2}u}{\partial z^{2}}]\\ \;\;\;\;\;\;-\frac{\chi_{0}\beta_{0}}{2\mu_{0}}\frac{\partial }{\partial \;r}[\;(\Theta -\Theta_{0})\;{\left(\vec{b}\right)}^{2}] \end{array}$$ (A18a)

Component *ϕ*:

$$\begin{array}{l} \frac{\partial \;v}{\partial \;t}+u\frac{\partial \;v}{\partial \;r}+\frac{v}{r}\frac{\partial \;v}{\partial \phi }+\frac{u\;v}{r}+w\frac{\partial \;v}{\partial \;z}=-\frac{1}{\rho_{0}}\frac{1}{r}\frac{\partial \;p}{\partial \;\phi }\;\\ \;\;\;\;\;\;+\nu \;[\frac{\partial }{\partial r}\{\frac{1}{r}\cdot \frac{\partial \;(r\cdot v)}{\partial r}\}+\frac{1}{r^{2}}\frac{\partial^{2}v}{\partial \phi^{2}}+\frac{2}{r^{2}}\frac{\partial \;u}{\partial \phi }+\frac{\partial^{2}v}{\partial z^{2}}]\\ \;\;\;\;\;\;-\frac{\chi_{0}\beta_{0}}{2\mu_{0}}\frac{1}{r}\frac{\partial }{\partial \phi }[(\Theta -\Theta_{0})\;b^{2}] \end{array}$$ (A18b)

Component *z*:

$$\begin{array}{l} \frac{\partial \;w}{\partial \;t}+u\frac{\partial \;w}{\partial \;r}+\frac{v}{r}\frac{\partial \;w}{\partial \varphi }+w\frac{\partial \;w}{\partial \;z}=-\frac{1}{\rho_{0}}\frac{\partial \;p}{\partial \;z}\;\\ \;\;\;\;\;\;+\nu \;[\frac{1}{r}\cdot \frac{\partial }{\partial r}(r\cdot \frac{\partial \;w}{\partial r})+\frac{1}{r^{2}}\frac{\partial^{2}w}{\partial \phi^{2}}+\frac{\partial^{2}w}{\partial z^{2}}]\\ \;\;\;\;\;\;-\beta_{0}(\Theta -\Theta_{0})\;g\;-\frac{\chi_{0}\beta_{0}}{2\mu_{0}}\frac{\partial }{\partial z}[\;(\Theta -\Theta_{0})\;b^{2}] \end{array}$$ (A18c)

Next, nondimensionalization is performed by the method of Hellums and Churchill.

$$\begin{array}{l} \frac{r}{r_{0}}\;=\;R,\;\phi \;=\;\theta ,\;\frac{z}{r_{0}}\;=\;Z,\;\frac{t}{t_{0}}\;=\;\tau ,\\ \frac{u}{u_{0}}\;=\;U,\;\frac{v}{u_{0}}\;=\;V,\;\frac{w}{u_{0}}\;=\;W,\;\frac{\Theta \;-\;\Theta_{0}}{\Theta_{hot}\;-\;\Theta_{cold}}\;=\;T,\\ \frac{p}{p_{0}}\;=\;P,\;u_{0}\;=\;\frac{\alpha }{h_{z}},\;t_{0}=\frac{r_{0}}{u_{0}}\;=\;\frac{r_{0}{}^{2}}{\alpha },\;\frac{b}{b_{0}}\;=\;B. \end{array}$$ (A19)

It is assumed that the following relationship holds:

$$\begin{array}{l} \frac{r_{0}}{t_{0}\;u_{0}}=1,\;\frac{p_{0}}{\rho_{0}u_{0}^{2}}=1,\;\frac{\alpha }{\rho_{0}u_{0}^{2}}=1,\;\frac{r_{0}}{h_{z}}=\;1,\\ Pr=\frac{\nu }{\alpha },\;Gr=\frac{\text{g}\beta_{0}\;(\Theta_{hot}-\Theta_{cold})\;h_{z}^{3}}{\nu^{2}},\;\gamma =\frac{\chi_{0}\;b_{0}^{2}}{\mu_{0}\;g\;h_{z}} \end{array}$$ (A20)

By substituting Eqs (A19) and (A20) for Eq (A18a),

$$\begin{array}{l} \frac{\partial \;(u_{0}U)}{\partial \;(t_{0}\tau )}+u_{0}U\frac{\partial \;(u_{0}U)}{\partial \;(r_{0}R)}+\frac{u_{0}V}{r_{0}R}\frac{\partial \;(u_{0}U)}{\partial \theta }-\frac{{\left(u_{0}V\right)}^{2}}{r_{0}R}+u_{0}W\frac{\partial \;(u_{0}U)}{\partial \;(r_{0}Z)}\\ =-\frac{1}{\rho_{0}}\frac{\partial \;(p_{0}P)}{\partial \;(r_{0}R)}\;+\nu \;[\frac{\partial }{\partial \;(r_{0}R)}\{\frac{1}{r_{0}R}\cdot \frac{\partial \;(r_{0}R\cdot u_{0}U)}{\partial \;(r_{0}R)}\}+\frac{1}{{\left(r_{0}R\right)}^{2}}\frac{\partial^{2}(u_{0}U)}{\partial \theta^{2}}-\frac{2}{{\left(r_{0}R\right)}^{2}}\frac{\partial \;(u_{0}V)}{\partial \theta }+\frac{\partial^{2}(u_{0}U)}{\partial \;{\left(r_{0}Z\right)}^{2}}]\\ -\frac{\chi_{0}\beta_{0}}{2\mu_{0}}\frac{\partial }{\partial \;(r_{0}R)}[(\Theta_{hot}-\Theta_{cold})\;T\;{\left(b_{0}B\right)}^{2}] \end{array}$$ (A20a)

By substituting Eqs (A19) and (A20) for Eq (A18b),

$$\begin{array}{l} \frac{\partial \;(u_{0}V)}{\partial \;(t_{0}\tau )}+u_{0}U\frac{\partial \;(u_{0}V)}{\partial \;(r_{0}R)}+\frac{u_{0}V}{r_{0}R}\frac{\partial \;(u_{0}V)}{\partial \theta }+\frac{u_{0}U\cdot u_{0}V}{r_{0}R}+u_{0}W\frac{\partial \;(u_{0}V)}{\partial \;(r_{0}Z)}\\ =-\frac{1}{\rho_{0}}\frac{1}{r_{0}R}\frac{\partial \;(p_{0}P)}{\partial \;\theta }\;+\nu \;[\frac{\partial }{\partial \;(r_{0}R)}\{\frac{1}{r_{0}R}\cdot \frac{\partial \;(r_{0}R\cdot u_{0}V)}{\partial \;(r_{0}R)}\}+\frac{1}{{\left(r_{0}R\right)}^{2}}\frac{\partial^{2}(u_{0}V)}{\partial \theta^{2}}+\frac{2}{{\left(r_{0}R\right)}^{2}}\frac{\partial \;(u_{0}U)}{\partial \theta }+\frac{\partial^{2}(u_{0}V)}{\partial \;{\left(r_{0}Z\right)}^{2}}]\\ -\frac{\chi_{0}\beta_{0}}{2\mu_{0}}\frac{1}{r_{0}R}\frac{\partial }{\partial \;\theta }[(\Theta_{hot}-\Theta_{cold})\;T\;{\left(b_{0}B\right)}^{2}] \end{array}$$ (A20b)

By substituting Eqs (A19) and (A20) for Eq (A18c),

$$\begin{array}{l} \frac{\partial \;(u_{0}W)}{\partial \;(t_{0}\tau )}+u_{0}U\frac{\partial \;(u_{0}W)}{\partial \;(r_{0}R)}+\frac{u_{0}V}{r_{0}R}\frac{\partial \;(u_{0}W)}{\partial \theta }+u_{0}W\frac{\partial \;(u_{0}W)}{\partial \;(r_{0}Z)}\\ =-\frac{1}{\rho_{0}}\frac{\partial \;(p_{0}P)}{\partial \;(r_{0}R)}\;+\nu \;\left[\frac{1}{r_{0}R}\frac{\partial }{\partial \;(r_{0}R)}\left\{r_{0}R\frac{\partial \;(u_{0}W)}{\partial \;(r_{0}R)}\right\}+\frac{1}{{\left(r_{0}R\right)}^{2}}\frac{\partial^{2}(u_{0}W)}{\partial \theta^{2}}+\frac{\partial^{2}(u_{0}W)}{\partial \;{\left(r_{0}Z\right)}^{2}}\right]\\ -g\;\beta_{0}(\Theta_{hot}-\Theta_{cold})\;T\;-\frac{\chi_{0}\beta_{0}}{2\mu_{0}}\frac{\partial }{\partial \;(r_{0}Z)}[(\Theta_{hot}-\Theta_{cold})\;T\;{\left(b_{0}B\right)}^{2}] \end{array}$$ (A20c)

Both sides are multiplied by $\frac{r_{0}}{u_{0}^{2}}$ and deformed.

$$\begin{array}{l} (\frac{r_{0}}{u_{0}t_{0}})\frac{\partial \;U}{\partial \;\tau }+U\frac{\partial \;U}{\partial \;R}+\frac{V}{R}\frac{\partial \;U}{\partial \theta }-\frac{V^{2}}{R}+W\frac{\partial \;U}{\partial \;Z}\\ =-\frac{p_{0}}{\rho_{0}u_{0}^{2}}\frac{\partial \;P}{\partial \;R}\;+\frac{r_{0}\;\nu }{u_{0}^{2}}\;\left[\frac{r_{0}\;u_{0}}{r_{0}^{3}}\frac{\partial }{\partial \;R}\left\{\frac{1}{R}\cdot \frac{\partial \;(R\cdot U)}{\partial \;R}\right\}+\frac{u_{0}}{r_{0}^{2}}\frac{1}{R^{2}}\frac{\partial^{2}U}{\partial \theta^{2}}-\frac{u_{0}}{r_{0}^{2}}\frac{2}{R^{2}}\frac{\partial \;V}{\partial \theta }+\frac{u_{0}}{r_{0}^{2}}\frac{\partial^{2}U}{\partial \;Z^{2}}\right]\\ -\frac{r_{0}}{u_{0}^{2}}\frac{\chi_{0}\beta_{0}}{2\mu_{0}}b_{0}^{2}\frac{\left(\Theta_{hot}-\Theta_{cold}\right)}{r_{0}}\frac{\partial \;(T\cdot \;B^{2})}{\partial \;R} \end{array}$$ (A21a)

$$\begin{array}{l} \left(\frac{r_{0}}{u_{0}t_{0}}\right)\frac{\partial \;V}{\partial \;\tau }+U\frac{\partial \;V}{\partial \;R}+\frac{V}{R}\frac{\partial \;V}{\partial \theta }+\frac{U\cdot V}{R}+W\frac{\partial \;V}{\partial \;Z}\\ =\;-\frac{p_{0}}{\rho_{0}u_{0}^{2}}\frac{1}{R}\frac{\partial \;P}{\partial \;\theta }\;+\frac{r_{0}\;\nu }{u_{0}^{2}}\;\left[\frac{u_{0}}{r_{0}^{2}}\frac{\partial }{\partial \;R}\left\{\frac{1}{R}\cdot \frac{\partial \;(R\cdot V)}{\partial \;R}\right\}+\frac{u_{0}}{r_{0}^{2}}\frac{1}{R^{2}}\frac{\partial^{2}V}{\partial \theta^{2}}+\frac{u_{0}}{r_{0}^{2}}\frac{2}{R^{2}}\frac{\partial \;U}{\partial \theta }+\frac{u_{0}}{r_{0}^{2}}\frac{\partial^{2}V}{\partial \;Z^{2}}\right]\\ \;-\frac{r_{0}}{u_{0}^{2}}\frac{\chi_{0}}{2\mu_{0}}\frac{g\;\beta_{0}\left(\Theta_{hot}-\Theta_{cold}\right)\;r_{0}^{3}}{\nu^{2}}\frac{\nu^{2}}{g\;r_{0}^{3}}\frac{b_{0}^{2}}{r_{0}}\frac{1}{R}\frac{\partial \;(T\cdot \;B^{2})}{\partial \;\theta } \end{array}$$ (A21b)

$$\begin{array}{l} (\frac{r_{0}}{u_{0}t_{0}})\frac{\partial \;W}{\partial \;\tau }+U\frac{\partial \;W}{\partial \;R}+\frac{V}{R}\frac{\partial \;W}{\partial \theta }+W\frac{\partial \;W}{\partial \;Z}\\ =\;-\frac{p_{0}}{\rho_{0}u_{0}^{2}}\frac{\partial \;P}{\partial \;Z}\;+\;\frac{r_{0}\;\nu }{u_{0}^{2}}\;\left[\frac{u_{0}}{r_{0}^{2}}\frac{1}{R}\frac{\partial }{\partial \;R}\left\{R\;\frac{\partial \;W}{\partial \;R}\right\}+\frac{u_{0}}{r_{0}^{2}}\frac{1}{R^{2}}\frac{\partial^{2}W}{\partial \theta^{2}}+\frac{u_{0}}{r_{0}^{2}}\frac{\partial^{2}W}{\partial \;Z^{2}}\right]\\ \;-\frac{r_{0}}{u_{0}^{2}}\frac{g\;\beta_{0}(\Theta_{hot}-\Theta_{cold})\;r_{0}^{3}}{\nu^{2}}\;\frac{\nu^{2}}{r_{0}^{3}}T\;-\frac{r_{0}}{u_{0}^{2}}\frac{\chi_{0}}{2\mu_{0}}\frac{g\;\beta_{0}(\Theta_{hot}-\Theta_{cold})\;r_{0}^{3}}{\nu^{2}}\frac{\nu^{2}}{g\;r_{0}^{3}}\frac{b_{0}^{2}}{r_{0}}\frac{\partial \;(T\cdot \;B^{2})}{\partial \;Z} \end{array}$$ (A21c)

The relational expression of $\frac{r_{0}}{t_{0}\;u_{0}}=1,\;\frac{\alpha }{r_{0}\;u_{0}}=1,\;\frac{p_{0}}{\rho_{0}u_{0}^{2}}=1$ is substituted and the other relational expression of $Pr=\frac{\nu }{\alpha },\;Gr=\frac{g\;\beta_{0}\;(\Theta_{hot}-\Theta_{cold})\;h_{z}^{3}}{\nu^{2}},\;\gamma =\frac{\chi_{0}\;b_{0}^{2}}{\mu_{0}\;g\;h_{z}}$ is introduced. Then, Eqs (A21a), (A21b) and (A21c) are arranged as follows:

$$\begin{array}{l} \frac{\partial \;U}{\partial \;\tau }+U\frac{\partial \;U}{\partial \;R}+\frac{V}{R}\frac{\partial \;U}{\partial \theta }-\frac{V^{2}}{R}+W\frac{\partial \;U}{\partial \;Z}=-\frac{\partial \;P}{\partial \;R}\;\\ +Pr\;\left[\frac{\partial }{\partial \;R}\left\{\frac{1}{R}\cdot \frac{\partial \;(R\cdot U)}{\partial \;R}\right\}+\frac{1}{R^{2}}\frac{\partial^{2}U}{\partial \theta^{2}}-\frac{2}{R^{2}}\frac{\partial \;V}{\partial \theta }+\frac{\partial^{2}U}{\partial \;Z^{2}}\right]-\frac{1}{2}\cdot \gamma \cdot Pr\cdot Ra\cdot \;\frac{\partial \;(T\cdot \;B^{2})}{\partial \;R} \end{array}$$ (A22a)

$$\begin{array}{l} \frac{\partial \;V}{\partial \;\tau }+U\frac{\partial \;V}{\partial \;R}+\frac{V}{R}\frac{\partial \;V}{\partial \theta }+\frac{U\cdot V}{R}+W\frac{\partial \;V}{\partial \;Z}=-\frac{1}{R}\frac{\partial \;P}{\partial \;\theta }\;\\ +Pr\;\left[\frac{\partial }{\partial \;R}\left\{\frac{1}{R}\cdot \frac{\partial \;(R\cdot V)}{\partial \;R}\right\}+\frac{1}{R^{2}}\frac{\partial^{2}V}{\partial \theta^{2}}+\frac{2}{R^{2}}\frac{\partial \;U}{\partial \theta }+\frac{\partial^{2}V}{\partial \;Z^{2}}\right]-\frac{1}{2}\cdot \gamma \cdot Pr\cdot Ra\cdot \frac{1}{R}\frac{\partial \;(T\cdot \;B^{2})}{\partial \;\theta } \end{array}$$ (A22b)

$$\begin{array}{l} \frac{\partial \;W}{\partial \;\tau }+U\frac{\partial \;W}{\partial \;R}+\frac{V}{R}\frac{\partial \;W}{\partial \theta }+W\frac{\partial \;W}{\partial \;Z}=-\frac{\partial \;P}{\partial \;Z}\\ \;+Pr\;\left[\frac{1}{R}\frac{\partial }{\partial \;R}\left\{R\cdot \frac{\partial \;W}{\partial \;R}\right\}+\frac{1}{R^{2}}\frac{\partial^{2}W}{\partial \theta^{2}}+\frac{\partial^{2}W}{\partial \;Z^{2}}\right]-Pr\cdot Ra\cdot \;T\;-\frac{1}{2}\cdot \gamma \cdot Pr\cdot Ra\cdot \frac{\partial \;(T\cdot \;B^{2})}{\partial \;Z} \end{array}$$ (A22c)

## Appendix B

$$\rho_{0}\;g=\frac{\rho_{0}\;\chi_{\text{m}}}{\mu_{0}}b_{z}\frac{\partial b_{z}}{\partial z}$$

$$b_{z}\frac{\partial b_{z}}{\partial z}=\frac{\mu_{0}\;g}{\chi_{\text{m}}}=\frac{4\pi \times 10^{-7}\times 9.807}{0.905\times 10^{-8}}=1362\;\left[{\text{T}}^{2}/\text{m}\right]$$ (B1)

## Appendix C

$$13.00\times \sqrt{\frac{1361.75}{585.94}}=19.82\;\left[\text{T}\right]$$ (C1)

## Appendix D

$$\frac{19.82\times 9.16}{13.00}=13.97\;\left[\text{T}\right]$$ (D1)

## Appendix E

$$\Theta_{hot}-\Theta_{cold}=Ra\frac{\alpha \;\nu }{g\;\beta_{0}\;h_{z}{}^{3}}=7000\times \frac{1.456\times 10^{-7}\times 0.872\times 10^{-6}}{9.807\times 0.259\times 10^{-3}\times 0.005^{3}}=2.799\;[{}^{\circ}\text{C}]$$ (E1)

## Appendix F

According to Ref. [53, 54], the density of water is 998.2 kg/m3 at 25°C and 995.7 kg/m3 at 30°C, respectively.

At 25.1°C

$$\rho \;=\;998.2\;+\;(25.1-20.0)/(30.0-20.0)\times (995.7-998.2)\;=\;996.9\;[\text{kg}/{\text{m}}^{3}]$$ (F1)

At 27.9°C

$$\rho \;=\;998.2\;+\;(27.9-20.0)/(30.0-20.0)\times (995.7-998.2)\;=\;996.2\;[\text{kg}/{\text{m}}^{3}]$$ (F2)

$$\frac{d}{d\;z}(\;\rho (\Theta ))=\frac{996.9-996.2}{0.005}=140.0$$ (F3)

According to Appendix D, the magnetic flux density at P_*over*_ and P_*under*_ was 13.97 [T]. Then, the magnitude of *f*_sc_ was estimated as follows.

$$\frac{b^{2}\chi_{\text{m}}}{2\;\mu_{0}}\frac{d}{dz}(\;\rho (\Theta )\;)=\frac{13.97^{2}\times (-9.05\times 10^{-9})}{2\times 4\pi \times 10^{-7}}\times 140.0=-98.39\;[\text{N}]$$ (F4)

## Supporting Information

S1 File (ZIP)
