# Supplementary material for: Magnetothermal Convection of Water with the Presence or Absence of a Magnetic Force Acting on the Susceptibility Gradient
Source: PLoS One. 2016 Sep 8;11(9):e0160090. doi: 10.1371/journal.pone.0160090 (PMC5015909; doi:10.1371/journal.pone.0160090)
Supplement: S1 File — (ZIP) [file pone.0160090.s001.zip › S1 File Maki/code145_fsc_and_fb.pdf]

```
C**********************************************************************C
C      THREE DIMENSIONAL NUMERICAL COMPUTATION OF CZ BULK FLOW        C
C         AT THE InSb MELT                                             C
C                    July 20 , 94    Ver.1.0   Mitsuo IWAMOTO          C
C 94.08.10   V1.1 Rewrite for VP2600                                   C
C 94.08.11   V1.2 Add thr iteration for output                         C
C 94.08.11   V1.3 Mesh 15*26*15 --> 21*38*21                           C
C 94.08.13 BUG FIX : SUBROUTINE   COMUVW -- U-COMPONENT                C
C              TJD= ..../RDZ ---> *RDZ : TJU= ..../RDZ ---> *RDZ       C
C 94.08.25   V1.5 Mesh 21*38*21 --> 26*38*31                           C
C 94.09.01   Cell Re-number 15 (Mesh 37*26*51)                         C
C 94.11.15   TRN.TXT BUG FIX /U,V,W-COMPONENT                          C
C              AVEU = DSQRT(SSU)/DBLE(M)                               C
C              ---> = DSQRT(SSU/DBLE(M))                               C
C 94.11.16   ADD OF THE RESULTM1.TXT AND RESULTM2.TXT                  C
C 95.01.11   Rewrite PDATA1,PDATA2                                     C
C**********************************************************************C
C      97.02.07 modified fisher M.Akamatsu                             C
C**********************************************************************C
C      2000.10.31   Rewrite Maki                                       C
C**********************************************************************C
C      2002.10.12   Rewrite Maki  加熱面 Nu                             C
C**********************************************************************C
      IMPLICIT DOUBLE PRECISION(A-H,O-Z)
      INTEGER ICYL
      PARAMETER(IP=51,JP=81,KP=70)
      COMMON/MAPMT/   IDX,MAXIT
      COMMON/ICYL1/ ICYL
      COMMON/TA/ TAU
      COMMON/MATOTYU/MATOA,MATOB,MATOC,MATOD
      COMMON/NCMF/ MFORNC
C
C       COMMON/NUSSLT/ ANUL(IP,JP),AVENU,QB,QF
C*********************************************************************
C****************************C
C*****     OPEN I/O UNIT     *****C
C****************************C
      CALL OPNUT
C-------------------------------------------------------------------C
C               INPUT PARAMETERS ( FROM FILE UNIT 20 )              C
C-------------------------------------------------------------------C
      CALL INP
C-------------------------------------------------------------------C
C               CALCULATION GRID                                    C
C-------------------------------------------------------------------C
      CALL GRID
C-------------------------------------------------------------------C
C               INITIALIZATION OF VALUES                            C
C-------------------------------------------------------------------C
      LIMIT=0
      CALL INIT(IDX)
C-------------------------------------------------------------------C
C               NUSS CALCURATE T-COBNDUCTIVE                        C
C-------------------------------------------------------------------C
      CALL TCOND
C
C-------------------------------------------------------------------C
C               MAGNETIZING FORCE TERM                              C
```

```
C-----------------------------------------------------------------C
      IF(MFORNC.NE.111) GOTO 1000
      CALL JIKARYOKU
C
*******************************************************************
C-----------------------------------------------------------------C
C              START CYCLE                                         C
C-----------------------------------------------------------------C
 1000 CONTINUE
C
      IF(ICYL.EQ. MATOA) CALL OUTA
      IF(ICYL.EQ. MATOB) CALL OUTB
      IF(ICYL.EQ. MATOC) CALL OUTC
      IF(ICYL.EQ. MATOD) CALL OUTD
C
C-----   ADVANCE TIME    -----C
C
      CALL ADV(LIMIT)
C
C-----   COMPUTE INITIAL ITERATES U,V AND W    -----C
C
      CALL COMUVW
C
C-----   COMPUTE UPDATEDCELL PRESSURE AND VELOCITES    -----C
C
      ITER=1
 2000 CONTINUE
      IFLAG=0
      CALL PRSS(IFLAG)
C
C-----   HAS CONVERGENCE BEEN REACHED    -----C
C
      IF(IFLAG.EQ.0) GOTO 3000
      ITER=ITER+1
      IF(ITER.LT.10000) GOTO 2000
      WRITE(33,*) ' ***    THIS CALCULATION HAS DIVERGENCED***'
      GO TO 1
 3000 CONTINUE
C
C-----   ADVANCE TEMPERATURE    -----C
C
      CALL TMP
C
C-----   COMPUTE UPDATTED ELECTRIC S-POTENTIAL & CURRENT DENSITITIES --C
C
 2500 CONTINUE
C
C-----   LIST TRANSIENT VALU ------C
C
      CALL TRNST(ITER)
C
C-----   LIST RESULT    -----C
C
      IF(ICYL.GE.MAXIT) THEN
      GOTO 1
      ELSE
      GOTO 1000
      END IF
```

```
C
CCCCCCCCCCCCCCCCCCCCCCCCCCCCCCCCCCCCCCCCCCCCCCCCC
      GOTO 1000
C
    1 CALL OUT
      CALL TECOUT
      CALL DATAVIEW
C
      STOP
      END
C
C
C##################################################################C
C##################################################################C
C
C***********************************************C
      SUBROUTINE OPNUT
C***********************************************C
C --- OPEN I/O UNIT ---C
      OPEN(20,FILE='MA2PMT.txt'        ,STATUS='OLD')
      OPEN(21,FILE='mainp.txt'         ,STATUS='OLD')
      OPEN(33,FILE='MATRNVOL.txt'      ,STATUS='unknown')
      OPEN(41,FILE='maout.txt'         ,STATUS='unknown')
      OPEN(42,FILE='maouta.txt'        ,STATUS='unknown')
      OPEN(43,FILE='maoutb.txt'        ,STATUS='unknown')
      OPEN(44,FILE='maoutc.txt'        ,STATUS='unknown')
      OPEN(45,FILE='maoutd.txt'        ,STATUS='unknown')
      OPEN(51,FILE='matec.txt'         ,STATUS='unknown')
      OPEN(52,FILE='mateca.txt'        ,STATUS='unknown')
      OPEN(53,FILE='matecb.txt'        ,STATUS='unknown')
      OPEN(54,FILE='matecc.txt'        ,STATUS='unknown')
      OPEN(55,FILE='matecd.txt'        ,STATUS='unknown')
C
      OPEN(102,FILE='MAJIBAOUT.txt' ,STATUS='old')
      OPEN(109,FILE='DATAview.txt'   ,STATUS='unknown')
      RETURN
      END
CC*****************************************************************C
      SUBROUTINE INP
C*****************************************************************C
      IMPLICIT DOUBLE PRECISION(A-H,O-Z)
      INTEGER ICYL
      PARAMETER(IP=51,JP=81,KP=70)
      COMMON/MAPMT/   IDX,MAXIT
      COMMON/TIME/    DT
      COMMON/NODE/ NR,NR1,NTH,NTH1,NX,NX1,NY,NY1,NYC,NZ,NZ1,NWR,NWZ,NTHC
      COMMON/DIMLSS/   RA,PR,RER,REC,GRA
      COMMON/ITPMT/   OMEGA,EPS
      COMMON/INTVAL/ IOUT
      COMMON/RGRID/    R(IP),RD(IP),DR,DRC(IP),HR
      COMMON/THGRID/   TH(JP),THD(JP),DTH,DTHC(JP),HTH
      COMMON/XGRID/    X(IP),XD(IP),DX,DXC(IP),HX
      COMMON/YGRID/    Y(JP),YD(JP),DY,DYC(JP),HY
      COMMON/ZGRID/    Z(KP),ZD(KP),DZ,DZC(KP),HZ
      COMMON/ICYL1/ ICYL
      COMMON/TA/ TAU
      COMMON/MATOTYU/MATOA,MATOB,MATOC,MATOD
      COMMON/JIKA/ GANMA
```

```
      COMMON/FUNC/ZGETA,RGETA
      COMMON/LEAN/SLOPER,SLOPED,PAI
      COMMON/NCMF/ MFORNC
      COMMON/MAGLIQ/    CONST
      common/Teramoto/hosei
C
C----------------      MAPMT    -------------------------------------
      READ(20,50)ICYL,TAU
      READ(20,70)MFORNC
      READ(20,100)IDX,MAXIT,DT
      READ(20,120)MATOA,MATOB,MATOC,MATOD
      READ(20,150)OMEGA,EPS
      READ(20,200)NR,NTH,NZ,NWR,NWZ
      READ(20,220)HR,HZ
      READ(20,230)ZGETA,RGETA
      READ(20,300)IOUT
      READ(20,350)PR
      READ(20,400)RA
C        READ(20,450)GRA
      READ(20,500)GANMA,CONST
      READ(20,650)SLOPED
   50 FORMAT(/I9,D12.5)
   70 FORMAT(/I5)
  100 FORMAT(/I5,I10,D9.2)
  120 FORMAT(/4I9)
  150 FORMAT(/2D12.4)
  200 FORMAT(/5I5)
  220     FORMAT(/2D12.5)
  230 FORMAT(/2D12.5)
  300 FORMAT(/I5)
  350 FORMAT(/D12.5)
  400 FORMAT(/D12.5)
C   450 FORMAT(/D12.5)
  500 FORMAT(/2D12.5)
  650 FORMAT(/D12.5)
C
      WRITE(6,101)IDX,MFORNC,MAXIT,DT
      WRITE(6,121)MATOA,MATOB
      WRITE(6,122)MATOC,MATOD
      WRITE(6,151)OMEGA,EPS
      WRITE(6,201)NR,NTH,NZ,NWR,NWZ
      WRITE(6,221)HR,HZ
      WRITE(6,301)IOUT
      WRITE(6,351)PR
      WRITE(6,401)RA
C        WRITE(6,451)GRA
      WRITE(6,501)GANMA,CONST
      WRITE(6,651)SLOPED,SLOPER
C
  101 FORMAT(/' IDX= ',I5,'    MFORNC=',I5,'    MAXIT=',I10,'    DT=',D9.2)
  121 FORMAT('    maouta = ',I9,'    maoutb = ',I9)
  122 FORMAT('    maoutc = ',I9,'    maoutd = ',I9)
  151 FORMAT('    OMEGA=',D12.4,'          EPS=',D12.4)
  201 FORMAT(/5I5)
  221     FORMAT(/'             HR=',D12.5,'             HZ=',D12.5)
  301 FORMAT('         IOUT=',I5)
  351 FORMAT('             PR=',D12.5)
  401 FORMAT('             RA=',D12.5)
```

```
C    451 FORMAT('            GRA=',D12.5)
     501 FORMAT('        GANMA=',D12.5,'        CONST=',D12.5)
C    601 FORMAT('              Xm=',D12.3)
C
         G=9.80665D0
         PAI=3.141592654D0
         NR1 = NR + 1
         NTH1 = NTH + 1
         NZ1 = NZ + 1
         NTHC = NTH / 2 + 1
         SLOPER=(PAI/180D0)*SLOPED
         GRA=RA/PR
c 磁場勾配項の補正
c    容器中心とコイル中心間距離で補正
c            Hosei=2.0D0*3.141592653D0*SQRT(ZGETA**2+RGETA**2)
C
     651 FORMAT('      SLOPED=',D12.5,'      SLOPER=',D12.5)
C
         WRITE(33,1000) PR,GRA,RA
         WRITE(33,1010)IDX,MFORNC,MAXIT,DT
         WRITE(33,1210)MATOA,MATOB
         WRITE(33,1220)MATOC,MATOD
         WRITE(33,1510)OMEGA,EPS
         WRITE(33,*)'    NR    NTH      NZ    NWR    NWZ'
         WRITE(33,2010)NR,NTH,NZ,NWR,NWZ
         WRITE(33,2210)HR,HZ
         WRITE(33,3010)IOUT
         WRITE(33,5010)GANMA,CONST
         WRITE(33,6510)SLOPED,SLOPER
    1000 FORMAT(/' Pr =',1PD12.5,' Gr = ',1PD12.5,' Ra = ',1PD12.5)
         WRITE(33,1400)
    1400 FORMAT(/'        ICYL ','      TAU      ','           Vol.AVE(U)    ',
        &             ' Vol.AVE(V)    ',' Vol.AVE(W)    ',' Vol.AVE(T)    ',
        &             '    AVENUc    ','        AVENUh    ','      Iter    ')
C
         IF (IDX.EQ.100) THEN
         WRITE(33,1600)
    1600 FORMAT(/'           0          0.0               0.0                 0.0           ',
        &             '          0.0                    0.0                     0.0')
         END IF
C---------------------------------------
C
    1010 FORMAT(' IDX= ',I5,'    MFORNC=',I5,'    MAXIT=',I10,'    DT=',D9.2)
    1210 FORMAT('    maouta = ',I9,'    maoutb = ',I9)
    1220 FORMAT('    maoutc = ',I9,'    maoutd = ',I9)
    1510 FORMAT('    OMEGA=',D12.4,'          EPS=',D12.4)
    2010 FORMAT(5I5)
    2210        FORMAT(/'              HR=',D12.5,'              HZ=',D12.5)
    3010 FORMAT('          IOUT=',I5)
    5010 FORMAT('        GANMA=',D12.5,'        CONST=',D12.5)
C 6010 FORMAT('              Xm=',D12.3)
    6510 FORMAT('      SLOPED=',D12.5,'      SLOPER=',D12.5)
C---------------------------------------
         RETURN
         END
C*****************************************************************C
         SUBROUTINE GRID
C*****************************************************************C
```

```
      IMPLICIT DOUBLE PRECISION(A-H,O-Z)
      PARAMETER(IP=51,JP=81,KP=70)
      INTEGER ICYL
      COMMON/RGRID/      R(IP),RD(IP),DR,DRC(IP),HR
      COMMON/THGRID/    TH(JP),THD(JP),DTH,DTHC(JP),HTH
      COMMON/XGRID/      X(IP),XD(IP),DX,DXC(IP),HX
      COMMON/YGRID/      Y(JP),YD(JP),DY,DYC(JP),HY
      COMMON/ZGRID/      Z(KP),ZD(KP),DZ,DZC(KP),HZ
      COMMON/NODE/ NR,NR1,NTH,NTH1,NX,NX1,NY,NY1,NYC,NZ,NZ1,NWR,NWZ,NTHC
      COMMON/DIMLSS/    RA,PR,RER,REC,GRA
      COMMON/NAM/          NAME(10)
      COMMON/JIKA/ GANMA
      COMMON/PAR2/        ICYL,ITER
      COMMON/WARI/        DRIN,DZIN,DTHIN
      COMMON/LEAN/SLOPER,SLOPED,PAI
C
C---------------- R    MAKI --------------------
C
      RD(1)= 0.0D0
      NRR=NR-1
      DR=HR/DBLE(NRR)
      DO 40 I=2,NRR
      RD(I)=RD(I-1)+DR
   40 CONTINUE
      RD(NR)=HR
      RD(NR+1)=HR
      DO 21 I=2,NR
        R(I)=(RD(I-1)+RD(I))*0.5D0
   21 CONTINUE
      R(1)=-R(2)
C       R(1)=R(2)
      R(NR+1)=HR
c       R(NR1)=RD(NR)+DR*0.5D0
C
C---------------- TH    MAKI --------------------
C
      PAI=3.14159265358979D+0
      HTH=2.0D0*PAI
      DTH=HTH/DBLE(NTH-1)
      DO 30 J=1,NTH+1
        TH(J)=DTH*DBLE(J-2)
   30 CONTINUE
      DO 31 J=1,NTH
        THD(J)=(TH(J)+TH(J+1))*0.5D0
   31 CONTINUE
      THD(NTH+1)= THD(NTH)+DTH
C
C---------------- Z    MAKI --------------------
      ZD(1)= 0.0D0
      DZ=HZ/DBLE(NZ-1)
      DO 10 K=2,NZ-1
        ZD(K)=DZ*DBLE(K-1)
   10 CONTINUE
      ZD(NZ)=HZ
      Z(1)=0.0D0
      DO 11 K=2,NZ
        Z(K)=(ZD(K-1)+ZD(K))*0.5D0
   11 CONTINUE
```

```
      Z(NZ+1)=HZ
C---------------- Z    MAKI --------------------
C
         DZIN=1.0D0/DZ
         DRIN=1.0D0/DR
         DTHIN=1.0D0/DTH
C
C-----------------------------------
      RETURN
      END
C
C*****************************************************************C
      SUBROUTINE INIT(IDX)
C*****************************************************************C
      IMPLICIT DOUBLE PRECISION(A-H,O-Z)
      INTEGER ICYL
      PARAMETER(IP=51,JP=81,KP=70)
      COMMON/VELOC/U(IP,0:JP+1,KP),    V(IP,0:JP+1,KP),    W(IP,0:JP+1,KP)
     &                ,U2(IP,0:JP+1,KP) ,V2(IP,0:JP+1,KP) ,W2(IP,0:JP+1,KP)
     &                ,U3(IP,0:JP+1,KP) ,V3(IP,0:JP+1,KP) ,W3(IP,0:JP+1,KP)
     &                ,V4(IP,0:JP+1,KP)
      COMMON/PRESS/    P(IP,0:JP+1,KP)
      COMMON/TEMP/      T(IP,0:JP+1,KP),TN(IP,0:JP+1,KP),TCO(IP,0:JP+1,KP)
      COMMON/RGRID/      R(IP),RD(IP),DR,DRC(IP),HR
      COMMON/THGRID/    TH(JP),THD(JP),DTH,DTHC(JP),HTH
      COMMON/XGRID/      X(IP),XD(IP),DX,DXC(IP),HX
      COMMON/YGRID/      Y(JP),YD(JP),DY,DYC(JP),HY
      COMMON/ZGRID/      Z(KP),ZD(KP),DZ,DZC(KP),HZ
      COMMON/NODE/ NR,NR1,NTH,NTH1,NX,NX1,NY,NY1,NYC,NZ,NZ1,NWR,NWZ,NTHC
      COMMON/VELOC2/UX(IP,0:JP+1,KP),    VY(IP,0:JP+1,KP)
      COMMON/ICYL1/ ICYL
      COMMON/TA/ TAU
      COMMON/MATOTYU/MATOA,MATOB,MATOC,MATOD
      COMMON/JIKA/ GANMA
      COMMON/LEAN/SLOPER,SLOPED,PAI
C
C-------------------------------------------------C
      DO 100 I=1,NR1
      DO 100 K=1,NZ1
      DO 100 J=0,NTH1+1
        T(I,J,K)=0.0D0
        U(I,J,K)=0.0D0
        V(I,J,K)=0.0D0
        W(I,J,K)=0.0D0
        P(I,J,K)=0.0D0
  100 CONTINUE
CCCCCCCCCCCCCCCCCCCCCCCCCCCCCCCCCCCCCCCCCCCCCCCCCCCC

C-------------------------------------------------C
      IF(IDX.EQ.800) THEN
C-------------------------------------------------C
      WRITE(6,*)'継続計算をします。'
      DO 400 K=1,NZ1
      DO 400 J=1,NTH1
      DO 400 I=1,NR1
      READ(21,120)
     &U(I,J,K),V(I,J,K),W(I,J,K),
     &T(I,J,K),P(I,J,K)
```

```
  120 FORMAT(5D25.15)
  400 CONTINUE
C
      WRITE(6,*)'2 継続計算をします。'
CCCCCCCCCCCCCCCCCCCCCCCCCCCCCCCCCCCCCCCCCCCCCCCCCCC
      END IF
C
        CALL BND
C
        CALL TBND
C
      RETURN
      END
C
C
C********************************************************************C
      SUBROUTINE ADV(LIMIT)
C********************************************************************C
      IMPLICIT DOUBLE PRECISION(A-H,O-Z)
      INTEGER ICYL
      PARAMETER(IP=51,JP=81,KP=70)
      COMMON/VELOC/U(IP,0:JP+1,KP),    V(IP,0:JP+1,KP),    W(IP,0:JP+1,KP)
     &             ,U2(IP,0:JP+1,KP) ,V2(IP,0:JP+1,KP) ,W2(IP,0:JP+1,KP)
     &             ,U3(IP,0:JP+1,KP) ,V3(IP,0:JP+1,KP) ,W3(IP,0:JP+1,KP)
     &             ,V4(IP,0:JP+1,KP)
      COMMON/VELOCN/ UN(IP,0:JP+1,KP),VN(IP,0:JP+1,KP),WN(IP,0:JP+1,KP)
      COMMON/TEMP/      T(IP,0:JP+1,KP),TN(IP,0:JP+1,KP),TCO(IP,0:JP+1,KP)
      COMMON/TIME/      DT
      COMMON/NODE/ NR,NR1,NTH,NTH1,NX,NX1,NY,NY1,NYC,NZ,NZ1,NWR,NWZ,NTHC
      COMMON/ICYL1/ ICYL
      COMMON/TA/ TAU
      COMMON/FUNC/ZGETA,RGETA
      COMMON/LEAN/SLOPER,SLOPED,PAI
C
      TAU=TAU+DT
      ICYL=ICYL+1
      LIMIT=LIMIT+1
      DO 1000 I=1,NR1
C       DO 1000 J=0,NTH1+1(akamatu)
      DO 1000 J=0,NTH1+1
      DO 1000 K=1,NZ1
         TN(I,J,K)=T(I,J,K)
         UN(I,J,K)=U(I,J,K)
         VN(I,J,K)=V(I,J,K)
         WN(I,J,K)=W(I,J,K)
 1000 CONTINUE
      RETURN
      END
C********************************************************************C
      SUBROUTINE PRSS(IFLAG)
C********************************************************************C
      IMPLICIT DOUBLE PRECISION(A-H,O-Z)
      PARAMETER(IP=51,JP=81,KP=70)
      COMMON/VELOC/U(IP,0:JP+1,KP),    V(IP,0:JP+1,KP),    W(IP,0:JP+1,KP)
     &             ,U2(IP,0:JP+1,KP) ,V2(IP,0:JP+1,KP) ,W2(IP,0:JP+1,KP)
     &             ,U3(IP,0:JP+1,KP) ,V3(IP,0:JP+1,KP) ,W3(IP,0:JP+1,KP)
     &             ,V4(IP,0:JP+1,KP)
      COMMON/PRESS/    P(IP,0:JP+1,KP)
```

```
      COMMON/MAPMT/       IDX,MAXIT
      COMMON/TIME/     DT
      COMMON/RGRID/     R(IP),RD(IP),DR,DRC(IP),HR
      COMMON/THGRID/    TH(JP),THD(JP),DTH,DTHC(JP),HTH
      COMMON/XGRID/     X(IP),XD(IP),DX,DXC(IP),HX
      COMMON/YGRID/     Y(JP),YD(JP),DY,DYC(JP),HY
      COMMON/ZGRID/     Z(KP),ZD(KP),DZ,DZC(KP),HZ
      COMMON/NODE/ NR,NR1,NTH,NTH1,NX,NX1,NY,NY1,NYC,NZ,NZ1,NWR,NWZ,NTHC
      COMMON/ITPMT/    OMEGA,EPS
      COMMON/WARI/       DRIN,DZIN,DTHIN
      COMMON/JIKA/ GANMA
      COMMON/LEAN/SLOPER,SLOPED,PAI
C
C            RDR=1.0D0/DR**2
C            RDTH=1.0D0/DTH**2
C            RDZ=1.0D0/DZ**2
          DR2IN=1.0D0/DR**2
          DTH2IN=1.0D0/DTH**2
          DZ2IN=1.0D0/DZ**2
*VOCL LOOP,TEMP(IFLAG)
*VOCL LOOP,NOVREC(W)
      DO 1000 K=2,NZ
      DO 1000 J=2,NTH
      DO 1000 I=2,NR
C            R2=1.0D0/(R(I)*R(I))
C            RRDR=1.0D0/(R(I)*DR)
C            RRDTH=1.0D0/(R(I)*DTH)
          RRIN=1.0D0/(R(I)*R(I))
          RDRIN=1.0D0/(R(I)*DR)
          RDTHIN=1.0D0/(R(I)*DTH)

C            BETA=OMEGA/(2.0D0*DT*(RDR+RDTH*RRIN+RDZ))
          BETA=OMEGA/(2.0D0*DT*(DR2IN+DTH2IN*RRIN+DZ2IN))
C            D=RRDR*(RD(I)*U(I,J,K)-RD(I-1)*U(I-1,J,K))
C     &       +RRDTH*(V(I,J,K)-V(I,J-1,K))
C     &       +DZIN*(W(I,J,K)-W(I,J,K-1))
          D=RDRIN*(RD(I)*U(I,J,K)-RD(I-1)*U(I-1,J,K))
     &       +RDTHIN*(V(I,J,K)-V(I,J-1,K))
     &       +DZIN*(W(I,J,K)-W(I,J,K-1))
C
          IF(DABS(D).GT.EPS) IFLAG=1
          DLTP=-BETA*D
          P(I,J,K)=P(I,J,K)+DLTP
          U( I ,J,K)=U( I ,J,K)+DLTP*DT*DRIN
          U(I-1,J,K)=U(I-1,J,K)-DLTP*DT*DRIN
          V(I, J ,K)=V(I, J ,K)+DLTP*DT*RDTHIN
          V(I,J-1,K)=V(I,J-1,K)-DLTP*DT*RDTHIN
          W(I,J, K )=W(I,J, K )+DLTP*DT*DZIN
          W(I,J,K-1)=W(I,J,K-1)-DLTP*DT*DZIN
 1000 CONTINUE
      CALL BND
      RETURN
      END
C*******************************************************************C
      SUBROUTINE TMP
C*******************************************************************C
      IMPLICIT DOUBLE PRECISION(A-H,O-Z)
      PARAMETER(IP=51,JP=81,KP=70)
```

```
      COMMON/VELOC/U(IP,0:JP+1,KP),    V(IP,0:JP+1,KP),    W(IP,0:JP+1,KP)
     &                ,U2(IP,0:JP+1,KP) ,V2(IP,0:JP+1,KP) ,W2(IP,0:JP+1,KP)
     &                ,U3(IP,0:JP+1,KP) ,V3(IP,0:JP+1,KP) ,W3(IP,0:JP+1,KP)
     &                ,V4(IP,0:JP+1,KP)
      COMMON/TEMP/     T(IP,0:JP+1,KP),TN(IP,0:JP+1,KP),TCO(IP,0:JP+1,KP)
      COMMON/MAPMT/       IDX,MAXIT
      COMMON/TIME/     DT
      COMMON/RGRID/     R(IP),RD(IP),DR,DRC(IP),HR
      COMMON/THGRID/    TH(JP),THD(JP),DTH,DTHC(JP),HTH
      COMMON/XGRID/     X(IP),XD(IP),DX,DXC(IP),HX
      COMMON/YGRID/     Y(JP),YD(JP),DY,DYC(JP),HY
      COMMON/ZGRID/     Z(KP),ZD(KP),DZ,DZC(KP),HZ
      COMMON/NODE/ NR,NR1,NTH,NTH1,NX,NX1,NY,NY1,NYC,NZ,NZ1,NWR,NWZ,NTHC
      COMMON/WARI/       DRIN,DZIN,DTHIN
      COMMON/JIKA/ GANMA
      COMMON/LEAN/SLOPER,SLOPED,PAI
      COMMON/MAGLIQ/    CONST
C
      DO 1000 I=2,NR
      DO 1000 J=2,NTH
      DO 1000 K=2,NZ
C
c           RDR=1.0D0/(R(I)*DR)
c           RDTH=1.0D0/(R(I)*DTH)
         RDRIN=1.0D0/(R(I)*DR)
         RDTHIN=1.0D0/(R(I)*DTH)
C
         UE=U(I,J,K)
         UW=U(I-1,J,K)
         VA=V(I,J,K)
         VB=V(I,J-1,K)
         WD=W(I,J,K)
         WU=W(I,J,K-1)
C
C***************** 3UPWIND METHOD QUICK***********************
C
      IF((I.EQ.2).OR.(I.EQ.NR)) THEN
C
         TJE=RD(I)*(UE*(TN(I+1,J,K)+TN(I,J,K))*0.5D0
     &       -(1.0D0+DABS(UE)*DR*0.5D0)*(TN(I+1,J,K)-TN(I,J,K))*DRIN)
         TJW=RD(I-1)*(UW*(TN(I,J,K)+TN(I-1,J,K))*0.5D0
     &       -(1.0D0+DABS(UW)*DR*0.5D0)*(TN(I,J,K)-TN(I-1,J,K))*DRIN)
      ELSE
         TJE=RD( I )*(UE
     &       *(-TN(I+2,J,K)+9.0D0*TN(I+1,J,K)
     &       +9.0D0*TN(I,J,K)-TN(I-1,J,K))*0.0625D0
     &       -DABS(UE)
     &       *(-TN(I+2,J,K)+3.0D0*TN(I+1,J,K)
     &       -3.0D0*TN(I,J,K)+TN(I-1,J,K))
     &       *0.0625D0
     &       -(TN(I+1,J,K)-TN(I,J,K))*DRIN)
         TJW=RD( I-1 )*(UW
     &       *(-TN(I+1,J,K)+9.0D0*TN(I,J,K)
     &       +9.0D0*TN(I-1,J,K)-TN(I-2,J,K))*0.0625D0
     &       -DABS(UW)
     &       *(-TN(I+1,J,K)+3.0D0*TN(I,J,K)
     &       -3.0D0*TN(I-1,J,K)+TN(I-2,J,K))
     &       *0.0625D0
```

```
     &           -(TN(I,J,K)-TN(I-1,J,K))*DRIN)
        END IF
C
         TJA=VA
     &           *(-TN(I,J+2,K)+9.0D0*TN(I,J+1,K)
     &           +9.0D0*TN(I,J,K)-TN(I,J-1,K))*0.0625D0
     &           -DABS(VA)
     &           *(-TN(I,J+2,K)+3.0D0*TN(I,J+1,K)
     &           -3.0D0*TN(I,J,K)+TN(I,J-1,K))
     &           *0.0625D0
     &           -(TN(I,J+1,K)-TN(I,J,K))*RDTHIN
          TJB=VB
     &           *(-TN(I,J+1,K)+9.0D0*TN(I,J,K)
     &           +9.0D0*TN(I,J-1,K)-TN(I,J-2,K))*0.0625D0
     &           -DABS(VB)
     &           *(-TN(I,J+1,K)+3.0D0*TN(I,J,K)
     &           -3.0D0*TN(I,J-1,K)+TN(I,J-2,K))
     &           *0.0625D0
     &           -(TN(I,J,K)-TN(I,J-1,K))*RDTHIN
C
       IF((K.EQ.2).OR.(K.EQ.NZ)) THEN
C
          TJD=WD*(TN(I,J,K+1)+TN(I,J,K))*0.5D0
     &           -(1.0D0+DABS(WD)*DZ*0.5D0)
     &           *(TN(I,J,K+1)-TN(I,J,K))*DZIN
          TJU=WU*(TN(I,J,K)+TN(I,J,K-1))*0.5D0
     &           -(1.0D0+DABS(WU)*DZ*0.5D0)
     &           *(TN(I,J,K)-TN(I,J,K-1))*DZIN
C
       ELSE
C
          TJD=WD
     &           *(-TN(I,J,K+2)+9.0D0*TN(I,J,K+1)
     &           +9.0D0*TN(I,J,K)-TN(I,J,K-1))*0.0625D0
     &           -DABS(WD)
     &           *(-TN(I,J,K+2)+3.0D0*TN(I,J,K+1)
     &           -3.0D0*TN(I,J,K)+TN(I,J,K-1))
     &           *0.0625D0
     &           -(TN(I,J,K+1)-TN(I,J,K))*DZIN
           TJU=WU
     &           *(-TN(I,J,K+1)+9.0D0*TN(I,J,K)
     &           +9.0D0*TN(I,J,K-1)-TN(I,J,K-2))*0.0625D0
     &           -DABS(WU)
     &           *(-TN(I,J,K+1)+3.0D0*TN(I,J,K)
     &           -3.0D0*TN(I,J,K-1)+TN(I,J,K-2))
     &           *0.0625D0
     &           -(TN(I,J,K)-TN(I,J,K-1))*DZIN
       END IF
C
C*****************************************************************
          TJR=(TJE-TJW)*RDRIN
          TJTH=(TJA-TJB)*RDTHIN
          TJZ=(TJD-TJU)*DZIN
          T(I,J,K)=TN(I,J,K)+DT*(-TJR-TJTH-TJZ)
 1000 CONTINUE
       CALL TBND
       RETURN
       END
```

```
C*********************************************************************C
      SUBROUTINE TRNST(ITER)
C*********************************************************************C
      IMPLICIT DOUBLE PRECISION(A-H,O-Z)
      INTEGER ICYL
      PARAMETER(IP=51,JP=81,KP=70)
      COMMON/NUSSLT/ ANUL(IP,JP),AVENUh,AVENUc,QBc,QB,QBh,QFc,QFh
      COMMON/INTVAL/ IOUT
      COMMON/VELOC/U(IP,0:JP+1,KP),    V(IP,0:JP+1,KP),    W(IP,0:JP+1,KP)
     &             ,U2(IP,0:JP+1,KP) ,V2(IP,0:JP+1,KP) ,W2(IP,0:JP+1,KP)
     &             ,U3(IP,0:JP+1,KP) ,V3(IP,0:JP+1,KP) ,W3(IP,0:JP+1,KP)
     &             ,V4(IP,0:JP+1,KP)
      COMMON/RGRID/     R(IP),RD(IP),DR,DRC(IP),HR
      COMMON/THGRID/    TH(JP),THD(JP),DTH,DTHC(JP),HTH
      COMMON/XGRID/     X(IP),XD(IP),DX,DXC(IP),HX
      COMMON/YGRID/     Y(JP),YD(JP),DY,DYC(JP),HY
      COMMON/ZGRID/     Z(KP),ZD(KP),DZ,DZC(KP),HZ
      COMMON/NODE/ NR,NR1,NTH,NTH1,NX,NX1,NY,NY1,NYC,NZ,NZ1,NWR,NWZ,NTHC
      COMMON/TEMP/      T(IP,0:JP+1,KP),TN(IP,0:JP+1,KP),TCO(IP,0:JP+1,KP)
      COMMON/DIMLSS/    RA,PR,RER,REC,GRA
      COMMON/ICYL1/ ICYL
      COMMON/TA/ TAU
      COMMON/MATOTYU/MATOA,MATOB,MATOC,MATOD
      COMMON/JIKA/ GANMA
      COMMON/FUNC/ZGETA,RGETA
      COMMON/LEAN/SLOPER,SLOPED,PAI
      COMMON/NCMF/ MFORNC
      COMMON/MAGLIQ/    CONST
C
      IF(MOD(ICYL,20).NE.0) GOTO 1111
C
      CALL AVVEL2(AVEU2,AVEV2,AVEW2,AVET2)
      CALL NUSS
C
      WRITE(6,649) ICYL,TAU,ITER,MFORNC,AVEU2,AVEV2,NR,NTH,NZ,
     &AVEW2,AVET2,SLOPED,CONST,QFc,QBc,AVENUc,QFh,QBh,AVENUh,
     &HR,HZ,ZGETA,RGETA,PR,RA,GRA,GANMA

  649 FORMAT(/1H ,'ICYL(=ITERATION) =',I10,2X,'TAU=',D12.5,2X,
     &                              'IN.ITER=',I4,2X,'MFORNC=',I5,2X,
     &        /2X,'AVE.U.=',1PD12.5,2X,'AVE.V.=',1PD12.5,
     &         2X,'(NR:NTH:NZ)=','(',I4,' :',I4,' :',I4,' )'
     &        /2X,'AVE.W.=',1PD12.5,2X,'AVE.T.=',1PD12.5,
     &         2X,'SLOPED=',1PD10.3,1X,'CONST=',G10.3,
     &        /2X,'(QFc=',D12.5,'    QBc=',D12.5,')',
     &         5X,'AVE.NUSS.(QFc/QBc)=',G13.6,
     &        /2X,'(QFh=',D12.5,'    QBh=',D12.5,')',
     &         5X,'AVE.NUSS.(QFh/QBh)=',G13.6,
     &        /2X,'HR=',D12.5,2X,'HZ=',D12.5,
     &         2X,'ZGETA=',D12.5,2X,'RGETA=',D12.5,
     &        /2X,'PR=',D12.5,2X,'RA=',D12.5,
     &         2X,'      GR=',D12.5,2X,'GANMA=',D12.5)
      IF(MOD(ICYL,IOUT).NE.0) GOTO 1111
      WRITE(33,3000) ICYL,TAU,AVEU2,AVEV2,AVEW2,AVET2,AVENUc,AVENUh,
     &ITER
 3000 FORMAT(I9,E13.5,3(1PE13.5),3E13.5,I5)
 1111 RETURN
      END
```

```
c
CXXXXXXXXXXXXXXXXXXXXXXXXXXXXXXXXXXXXXXXXXXXXXXXXXXXXXXXXXXXXXXXXXXXXXX
C
CX                                                                    XC
CX         SUBROUTINE -> SOBROUTINE                                   XC
CX                                                                    XC
CXXXXXXXXXXXXXXXXXXXXXXXXXXXXXXXXXXXXXXXXXXXXXXXXXXXXXXXXXXXXXXXXXXXXXX
C
C
C
C*******************************************************************C
      SUBROUTINE AVVEL2(AVEU,AVEV,AVEW,AVET)
C*******************************************************************C
      IMPLICIT DOUBLE PRECISION(A-H,O-Z)
      PARAMETER(IP=51,JP=81,KP=70)
      COMMON/VELOC/U(IP,0:JP+1,KP),    V(IP,0:JP+1,KP),    W(IP,0:JP+1,KP)
     &            ,U2(IP,0:JP+1,KP) ,V2(IP,0:JP+1,KP) ,W2(IP,0:JP+1,KP)
     &            ,U3(IP,0:JP+1,KP) ,V3(IP,0:JP+1,KP) ,W3(IP,0:JP+1,KP)
     &            ,V4(IP,0:JP+1,KP)
      COMMON/TEMP/     T(IP,0:JP+1,KP),TN(IP,0:JP+1,KP),TCO(IP,0:JP+1,KP)
      COMMON/RGRID/     R(IP),RD(IP),DR,DRC(IP),HR
      COMMON/THGRID/   TH(JP),THD(JP),DTH,DTHC(JP),HTH
      COMMON/XGRID/     X(IP),XD(IP),DX,DXC(IP),HX
      COMMON/YGRID/     Y(JP),YD(JP),DY,DYC(JP),HY
      COMMON/ZGRID/     Z(KP),ZD(KP),DZ,DZC(KP),HZ
      COMMON/NODE/ NR,NR1,NTH,NTH1,NX,NX1,NY,NY1,NYC,NZ,NZ1,NWR,NWZ,NTHC
      COMMON/LEAN/SLOPER,SLOPED,PAI
C
         SSU = 0.0D0
         SSV = 0.0D0
         SSW = 0.0D0
         SST = 0.0D0
C--- U - COMPONENT ---
         TVOL=0.0D0
         VOL=(R(2)**2)*DZ/DBLE(NTH-1)
         DO 1000 J=2,NTH
         DO 1000 K=2,NZ
         SSU=SSU+(U(1,J,K)**2)*VOL
         TVOL=TVOL+VOL
 1000 CONTINUE
         DO 2000 I=2,NR-1
         VOL=(R(I+1)**2 - R(I)**2)*DZ/DBLE(NTH-1)
         DO 2000 J=2,NTH
         DO 2000 K=2,NZ
         SSU=SSU+(U(I,J,K)**2)*VOL
         TVOL=TVOL+VOL
 2000 CONTINUE
         VOL=(RD(NR)**2 - R(NR)**2)*DZ/DBLE(NTH-1)
         DO 3000 J=2,NTH
         DO 3000 K=2,NZ
         SSU=SSU+(U(NR,J,K)**2)*VOL
         TVOL=TVOL+VOL
 3000 CONTINUE
         AVEU=DSQRT(SSU/TVOL)
C
C------ V , T ------
         TVOL=0.0D0
         DO 4000 I=2,NR
```

```
            VOL=(RD(I)**2 - RD(I-1)**2)*DZ/DBLE(NTH-1)
            DO 4000 J=2,NTH
            DO 4000 K=2,NZ
            SSV=SSV+(V(I,J,K)**2)*VOL
            SST=SST+T(I,J,K)*VOL
            TVOL=TVOL+VOL
 4000 CONTINUE
            AVEV=DSQRT(SSV/TVOL)
            AVET=SST/TVOL
C
C--- W - COMPONENT ---
            TVOL=0.0D0
            DO 5000 I=2,NR
            VOL=(RD(I)**2-RD(I-1)**2)*DZ/DBLE(NTH-1)
            HVOL=VOL*0.5D0
            DO 5000 J=2,NTH
            SSW=SSW+(W(I,J,1)**2)*HVOL
            TVOL=TVOL+HVOL
            DO 6000 K=2,NZ-1
            SSW=SSW+(W(I,J,K)**2)*VOL
            TVOL=TVOL+VOL
 6000 CONTINUE
            SSW=SSW+(W(I,J,NZ)**2)*HVOL
            TVOL=TVOL+HVOL
 5000 CONTINUE
            AVEW=DSQRT(SSW/TVOL)
C
        RETURN
        END
C
CXXXXXXXXXXXXXXXXXXXXXXXXXXXXXXXXXXXXXXXXXXXXXXXXXXXXXXXXXXXXXXXXXXXXXX
C
CX                                                                    XC
CX          SUBROUTINE -> SOBROUTINE -> SUBROUTINE                        XC
CX                                                                    XC
CXXXXXXXXXXXXXXXXXXXXXXXXXXXXXXXXXXXXXXXXXXXXXXXXXXXXXXXXXXXXXXXXXXXXXX
C
C
C**********************************************C
        SUBROUTINE TCOND
C**********************************************C
        IMPLICIT DOUBLE PRECISION(A-H,O-Z)
        PARAMETER(IP=51,JP=81,KP=70)
        COMMON/TEMP/     T(IP,0:JP+1,KP),TN(IP,0:JP+1,KP),TCO(IP,0:JP+1,KP)
        COMMON/NODE/ NR,NR1,NTH,NTH1,NX,NX1,NY,NY1,NYC,NZ,NZ1,NWR,NWZ,NTHC
        COMMON/NUSSLT/ ANUL(IP,JP),AVENUh,AVENUc,QBc,QB,QBh,QFc,QFh
        COMMON/RGRID/     R(IP),RD(IP),DR,DRC(IP),HR
        COMMON/ZGRID/     Z(KP),ZD(KP),DZ,DZC(KP),HZ
        COMMON/LEAN/SLOPER,SLOPED,PAI
C
        DIMENSION DTDZFc(IP,JP),DTDZFh(IP,JP),DTDZB(IP,JP),
       &              TF(IP,0:JP+1,KP),TB(IP,0:JP+1,KP),
       &              TFc(IP,0:JP+1,KP),TFh(IP,0:JP+1,KP)
C****************************************************C
C
        DO 410 K=1,NZ1
        DO 410 I=1,NR1
        DO 410 J=0,NTH1+1
```

```
            TB(I,J,K)=-0.5D0+Z(K)/HZ
  410 CONTINUE
C
        CALL GRDNTc(DTDZBc,TB)
        CALL AVE2(QBc,DTDZBc)
C
        CALL GRDNTh(DTDZBh,TB)
        CALL AVE2(QBh,DTDZBh)
C
        RETURN
        END
C
C**********************************************C
        SUBROUTINE NUSS
C**********************************************C
        IMPLICIT DOUBLE PRECISION(A-H,O-Z)
        PARAMETER(IP=51,JP=81,KP=70)
        COMMON/TEMP/    T(IP,0:JP+1,KP),TN(IP,0:JP+1,KP),TCO(IP,0:JP+1,KP)
        COMMON/NODE/ NR,NR1,NTH,NTH1,NX,NX1,NY,NY1,NYC,NZ,NZ1,NWR,NWZ,NTHC
        COMMON/NUSSLT/ ANUL(IP,JP),AVENUh,AVENUc,QBc,QB,QBh,QFc,QFh
        COMMON/RGRID/    R(IP),RD(IP),DR,DRC(IP),HR
        COMMON/ZGRID/    Z(KP),ZD(KP),DZ,DZC(KP),HZ
C
        DIMENSION DTDZFc(IP,JP),DTDZFh(IP,JP),DTDZB(IP,JP),
       &            TF(IP,0:JP+1,KP),TB(IP,0:JP+1,KP),
       &            TFc(IP,0:JP+1,KP),TFh(IP,0:JP+1,KP)
C
C****************************************************C
        DO 400 K=1,NZ1
        DO 400 I=1,NR1
        DO 400 J=0,NTH1+1
            TFc(I,J,K)=T(I,J,K)
            TFh(I,J,K)=T(I,J,K)
  400 CONTINUE
C****************************************************C
        DO 500 I=1,NR1
        DO 500 J=0,NTH1+1
            TFc(I,J,1)=0.5D0*(TFc(I,J,1)+TFc(I,J,2))
            TFh(I,J,NZ1)=0.5D0*(TFh(I,J,NZ)+TFh(I,J,NZ1))
  500 CONTINUE
C
        CALL GRDNTc(DTDZFc,TFc)
        CALL AVE2(QFc,DTDZFc)
        AVENUc=QFc/QBc
C
        CALL GRDNTh(DTDZFh,TFh)
        CALL AVE2(QFh,DTDZFh)
        AVENUh=QFh/QBh
c
        RETURN
        END
C
C**********************************************C
        SUBROUTINE AVE2(QVOL,DTDZ)
C**********************************************C
        IMPLICIT DOUBLE PRECISION(A-H,O-Z)
        PARAMETER(IP=51,JP=81,KP=70)
        COMMON/RGRID/    R(IP),RD(IP),DR,DRC(IP),HR
```

```
      COMMON/THGRID/   TH(JP),THD(JP),DTH,DTHC(JP),HTH
      COMMON/XGRID/    X(IP),XD(IP),DX,DXC(IP),HX
      COMMON/YGRID/    Y(JP),YD(JP),DY,DYC(JP),HY
      COMMON/ZGRID/    Z(KP),ZD(KP),DZ,DZC(KP),HZ
      COMMON/NODE/ NR,NR1,NTH,NTH1,NX,NX1,NY,NY1,NYC,NZ,NZ1,NWR,NWZ,NTHC
C
      DIMENSION DTDZ(IP,JP),TT(IP,0:JP+1,KP)
      DIMENSION DTDZFc(IP,JP),DTDZFh(IP,JP),DTDZB(IP,JP),
     &          TF(IP,0:JP+1,KP),TB(IP,0:JP+1,KP),
     &          TFc(IP,0:JP+1,KP),TFh(IP,0:JP+1,KP)
C
C------ Q ------
      QVOL= 0.0D0
      VOL=0.0D0
C
      DO 4000 I=2,NR-1
      DO 4000 J=1,NTH-1
        VOL =0.5D00*(RD(I)**2 - RD(I-1)**2)*DTH
        QVOL=QVOL+DTDZ(I,J)*VOL
 4000 CONTINUE
C
      DO 5000 J=1,NTH-1
CS
        VOL =0.5D0*(RD(NR)**2 - RD(NR-1)**2)*DTH
        QVOL=QVOL+DTDZ(NR,J)*VOL
 5000 CONTINUE
C
      RETURN
      END
C
C***********************************************C
      SUBROUTINE GRDNTc(DTDZc,TT)
C***********************************************C
      IMPLICIT DOUBLE PRECISION(A-H,O-Z)
      PARAMETER(IP=51,JP=81,KP=70)
      COMMON/RGRID/    R(IP),RD(IP),DR,DRC(IP),HR
      COMMON/THGRID/   TH(JP),THD(JP),DTH,DTHC(JP),HTH
      COMMON/XGRID/    X(IP),XD(IP),DX,DXC(IP),HX
      COMMON/YGRID/    Y(JP),YD(JP),DY,DYC(JP),HY
      COMMON/ZGRID/    Z(KP),ZD(KP),DZ,DZC(KP),HZ
      COMMON/NODE/ NR,NR1,NTH,NTH1,NX,NX1,NY,NY1,NYC,NZ,NZ1,NWR,NWZ,NTHC
      COMMON/TEMP/    T(IP,0:JP+1,KP),TN(IP,0:JP+1,KP),TCO(IP,0:JP+1,KP)
      DIMENSION DTDZc(IP,JP),DTDZh(IP,JP),TT(IP,0:JP+1,KP)
CC
C 冷却面の平均 Nu 数
      DO 1000 I=2,NR
      DO 1000 J=1,NTH-1
        DTDZc(I,J)=(-8.0D0*TT(I,J,1)+9.0D0*TT(I,J,2)-TT(I,J,3))
     &             /(3.0D0*DZ)
 1000 CONTINUE
      RETURN
      END
C
C***********************************************C
      SUBROUTINE GRDNTh(DTDZh,TT)
C***********************************************C
      IMPLICIT DOUBLE PRECISION(A-H,O-Z)
      PARAMETER(IP=51,JP=81,KP=70)
```

```
      COMMON/RGRID/    R(IP),RD(IP),DR,DRC(IP),HR
      COMMON/THGRID/   TH(JP),THD(JP),DTH,DTHC(JP),HTH
      COMMON/XGRID/    X(IP),XD(IP),DX,DXC(IP),HX
      COMMON/YGRID/    Y(JP),YD(JP),DY,DYC(JP),HY
      COMMON/ZGRID/    Z(KP),ZD(KP),DZ,DZC(KP),HZ
      COMMON/NODE/ NR,NR1,NTH,NTH1,NX,NX1,NY,NY1,NYC,NZ,NZ1,NWR,NWZ,NTHC
      COMMON/TEMP/     T(IP,0:JP+1,KP),TN(IP,0:JP+1,KP),TCO(IP,0:JP+1,KP)
      DIMENSION DTDZc(IP,JP),DTDZh(IP,JP),TT(IP,0:JP+1,KP)
      DIMENSION A(IP,JP),B(IP,JP)
CC
C 加熱面の平均 Nu 数
      DET=(Z(NZ-1)**2.0D0-Z(NZ)**2.0D0)*0.5D0*DZ
     &-(Z(NZ)**2.0D0-ZD(NZ)**2.0D0)*DZ
C
      DO 1000 I=2,NR
      DO 1000 J=1,NTH-1
C
      A(I,J)=(
     &0.5D0*DZ*(TT(I,J,NZ-1)-TT(I,J,NZ))
     &-DZ*(TT(I,J,NZ)-TT(I,J,NZ1))
     &)/DET
C
      B(I,J)=(
     &(ZD(NZ)**2.0D0-Z(NZ)**2.0D0)*(TT(I,J,NZ-1)-TT(I,J,NZ))
     &-(Z(NZ-1)**2.0D0-Z(NZ)**2.0D0)*(TT(I,J,NZ)-TT(I,J,NZ1))
     &)/DET
CC
      DTDZh(I,J)=2.0D0*A(I,J)*ZD(NZ)+B(I,J)
C
 1000 CONTINUE
      RETURN
      END
C
C*****************************************************************C
      SUBROUTINE OUT
C*****************************************************************C
      IMPLICIT DOUBLE PRECISION(A-H,O-Z)
      PARAMETER(IP=51,JP=81,KP=70)
      COMMON/VELOC/U(IP,0:JP+1,KP),   V(IP,0:JP+1,KP),   W(IP,0:JP+1,KP)
     &            ,U2(IP,0:JP+1,KP) ,V2(IP,0:JP+1,KP) ,W2(IP,0:JP+1,KP)
     &            ,U3(IP,0:JP+1,KP) ,V3(IP,0:JP+1,KP) ,W3(IP,0:JP+1,KP)
     &            ,V4(IP,0:JP+1,KP)
      COMMON/PRESS/   P(IP,0:JP+1,KP)
      COMMON/TEMP/     T(IP,0:JP+1,KP),TN(IP,0:JP+1,KP),TCO(IP,0:JP+1,KP)
      COMMON/DIMLSS/   RA,PR,RER,REC,GRA
      COMMON/ITPMT/    OMEGA,EPS
      COMMON/RGRID/    R(IP),RD(IP),DR,DRC(IP),HR
      COMMON/THGRID/   TH(JP),THD(JP),DTH,DTHC(JP),HTH
      COMMON/XGRID/    X(IP),XD(IP),DX,DXC(IP),HX
      COMMON/YGRID/    Y(JP),YD(JP),DY,DYC(JP),HY
      COMMON/ZGRID/    Z(KP),ZD(KP),DZ,DZC(KP),HZ
      COMMON/NODE/ NR,NR1,NTH,NTH1,NX,NX1,NY,NY1,NYC,NZ,NZ1,NWR,NWZ,NTHC
      COMMON/ELEF/     EU(IP,0:JP+1,KP),EV(IP,0:JP+1,KP),EW(IP,0:JP+1,KP)
      COMMON/VELOC2/UX(IP,0:JP+1,KP),   VY(IP,0:JP+1,KP)
      COMMON/MATOTYU/MATOA,MATOB,MATOC,MATOD
C
      DO 300 K=1,NZ1
      DO 300 J=1,NTH1
```

```
      DO 300 I=1,NR1
      WRITE(41,60)
     &U(I,J,K),V(I,J,K),W(I,J,K),
     &T(I,J,K),P(I,J,K)
   60 FORMAT(5D25.15)
  300 CONTINUE
C
      RETURN
      END
C
C*****************************************************************C
      SUBROUTINE OUTA
C*****************************************************************C
      IMPLICIT DOUBLE PRECISION(A-H,O-Z)
      PARAMETER(IP=51,JP=81,KP=70)
      COMMON/VELOC/U(IP,0:JP+1,KP),    V(IP,0:JP+1,KP),    W(IP,0:JP+1,KP)
     &            ,U2(IP,0:JP+1,KP) ,V2(IP,0:JP+1,KP) ,W2(IP,0:JP+1,KP)
     &            ,U3(IP,0:JP+1,KP) ,V3(IP,0:JP+1,KP) ,W3(IP,0:JP+1,KP)
     &            ,V4(IP,0:JP+1,KP)
      COMMON/PRESS/    P(IP,0:JP+1,KP)
      COMMON/TEMP/      T(IP,0:JP+1,KP),TN(IP,0:JP+1,KP),TCO(IP,0:JP+1,KP)
      COMMON/DIMLSS/    RA,PR,RER,REC,GRA
      COMMON/ITPMT/    OMEGA,EPS
      COMMON/RGRID/      R(IP),RD(IP),DR,DRC(IP),HR
      COMMON/THGRID/    TH(JP),THD(JP),DTH,DTHC(JP),HTH
      COMMON/XGRID/      X(IP),XD(IP),DX,DXC(IP),HX
      COMMON/YGRID/      Y(JP),YD(JP),DY,DYC(JP),HY
      COMMON/ZGRID/      Z(KP),ZD(KP),DZ,DZC(KP),HZ
      COMMON/NODE/ NR,NR1,NTH,NTH1,NX,NX1,NY,NY1,NYC,NZ,NZ1,NWR,NWZ,NTHC
      COMMON/ELEF/      EU(IP,0:JP+1,KP),EV(IP,0:JP+1,KP),EW(IP,0:JP+1,KP)
      COMMON/VELOC2/UX(IP,0:JP+1,KP),    VY(IP,0:JP+1,KP)
      COMMON/MATOTYU/MATOA,MATOB,MATOC,MATOD
C
      DO 300 K=1,NZ1
      DO 300 J=1,NTH1
      DO 300 I=1,NR1
      WRITE(42,60)
     &U(I,J,K),V(I,J,K),W(I,J,K),
     &T(I,J,K),P(I,J,K)
   60 FORMAT(5D25.15)
  300 CONTINUE
C
      CALL TECOUTA
      RETURN
      END
C*****************************************************************C
      SUBROUTINE OUTB
C*****************************************************************C
      IMPLICIT DOUBLE PRECISION(A-H,O-Z)
      PARAMETER(IP=51,JP=81,KP=70)
      COMMON/VELOC/U(IP,0:JP+1,KP),    V(IP,0:JP+1,KP),    W(IP,0:JP+1,KP)
     &            ,U2(IP,0:JP+1,KP) ,V2(IP,0:JP+1,KP) ,W2(IP,0:JP+1,KP)
     &            ,U3(IP,0:JP+1,KP) ,V3(IP,0:JP+1,KP) ,W3(IP,0:JP+1,KP)
     &            ,V4(IP,0:JP+1,KP)
      COMMON/PRESS/    P(IP,0:JP+1,KP)
      COMMON/TEMP/      T(IP,0:JP+1,KP),TN(IP,0:JP+1,KP),TCO(IP,0:JP+1,KP)
      COMMON/DIMLSS/    RA,PR,RER,REC,GRA
      COMMON/ITPMT/    OMEGA,EPS
```

```
      COMMON/RGRID/    R(IP),RD(IP),DR,DRC(IP),HR
      COMMON/THGRID/   TH(JP),THD(JP),DTH,DTHC(JP),HTH
      COMMON/XGRID/    X(IP),XD(IP),DX,DXC(IP),HX
      COMMON/YGRID/    Y(JP),YD(JP),DY,DYC(JP),HY
      COMMON/ZGRID/    Z(KP),ZD(KP),DZ,DZC(KP),HZ
      COMMON/NODE/ NR,NR1,NTH,NTH1,NX,NX1,NY,NY1,NYC,NZ,NZ1,NWR,NWZ,NTHC
      COMMON/ELEF/     EU(IP,0:JP+1,KP),EV(IP,0:JP+1,KP),EW(IP,0:JP+1,KP)
      COMMON/VELOC2/UX(IP,0:JP+1,KP),   VY(IP,0:JP+1,KP)
      COMMON/MATOTYU/MATOA,MATOB,MATOC,MATOD
C
      DO 300 K=1,NZ1
      DO 300 J=1,NTH1
      DO 300 I=1,NR1
      WRITE(43,60)
     &U(I,J,K),V(I,J,K),W(I,J,K),
     &T(I,J,K),P(I,J,K)
   60 FORMAT(5D25.15)
  300 CONTINUE
C
      CALL TECOUTB
      RETURN
      END
C*****************************************************************C
      SUBROUTINE OUTC
C*****************************************************************C
      IMPLICIT DOUBLE PRECISION(A-H,O-Z)
      PARAMETER(IP=51,JP=81,KP=70)
      COMMON/VELOC/U(IP,0:JP+1,KP),   V(IP,0:JP+1,KP),   W(IP,0:JP+1,KP)
     &             ,U2(IP,0:JP+1,KP) ,V2(IP,0:JP+1,KP) ,W2(IP,0:JP+1,KP)
     &             ,U3(IP,0:JP+1,KP) ,V3(IP,0:JP+1,KP) ,W3(IP,0:JP+1,KP)
     &             ,V4(IP,0:JP+1,KP)
      COMMON/PRESS/   P(IP,0:JP+1,KP)
      COMMON/TEMP/     T(IP,0:JP+1,KP),TN(IP,0:JP+1,KP),TCO(IP,0:JP+1,KP)
      COMMON/DIMLSS/   RA,PR,RER,REC,GRA
      COMMON/ITPMT/   OMEGA,EPS
      COMMON/RGRID/    R(IP),RD(IP),DR,DRC(IP),HR
      COMMON/THGRID/   TH(JP),THD(JP),DTH,DTHC(JP),HTH
      COMMON/XGRID/    X(IP),XD(IP),DX,DXC(IP),HX
      COMMON/YGRID/    Y(JP),YD(JP),DY,DYC(JP),HY
      COMMON/ZGRID/    Z(KP),ZD(KP),DZ,DZC(KP),HZ
      COMMON/NODE/ NR,NR1,NTH,NTH1,NX,NX1,NY,NY1,NYC,NZ,NZ1,NWR,NWZ,NTHC
      COMMON/ELEF/     EU(IP,0:JP+1,KP),EV(IP,0:JP+1,KP),EW(IP,0:JP+1,KP)
      COMMON/VELOC2/UX(IP,0:JP+1,KP),   VY(IP,0:JP+1,KP)
      COMMON/MATOTYU/MATOA,MATOB,MATOC,MATOD
C
      DO 300 K=1,NZ1
      DO 300 J=1,NTH1
      DO 300 I=1,NR1
      WRITE(44,60)
     &U(I,J,K),V(I,J,K),W(I,J,K),
     &T(I,J,K),P(I,J,K)
   60 FORMAT(5D25.15)
  300 CONTINUE
C
      CALL TECOUTC
      RETURN
      END
C*****************************************************************C
```

```
      SUBROUTINE OUTD
C*****************************************************************C
      IMPLICIT DOUBLE PRECISION(A-H,O-Z)
      PARAMETER(IP=51,JP=81,KP=70)
      COMMON/VELOC/U(IP,0:JP+1,KP),    V(IP,0:JP+1,KP),    W(IP,0:JP+1,KP)
     &                   ,U2(IP,0:JP+1,KP) ,V2(IP,0:JP+1,KP) ,W2(IP,0:JP+1,KP)
     &                   ,U3(IP,0:JP+1,KP) ,V3(IP,0:JP+1,KP) ,W3(IP,0:JP+1,KP)
     &                   ,V4(IP,0:JP+1,KP)
      COMMON/PRESS/    P(IP,0:JP+1,KP)
      COMMON/TEMP/      T(IP,0:JP+1,KP),TN(IP,0:JP+1,KP),TCO(IP,0:JP+1,KP)
      COMMON/DIMLSS/    RA,PR,RER,REC,GRA
      COMMON/ITPMT/    OMEGA,EPS
      COMMON/RGRID/      R(IP),RD(IP),DR,DRC(IP),HR
      COMMON/THGRID/    TH(JP),THD(JP),DTH,DTHC(JP),HTH
      COMMON/XGRID/      X(IP),XD(IP),DX,DXC(IP),HX
      COMMON/YGRID/      Y(JP),YD(JP),DY,DYC(JP),HY
      COMMON/ZGRID/      Z(KP),ZD(KP),DZ,DZC(KP),HZ
      COMMON/NODE/ NR,NR1,NTH,NTH1,NX,NX1,NY,NY1,NYC,NZ,NZ1,NWR,NWZ,NTHC
      COMMON/ELEF/      EU(IP,0:JP+1,KP),EV(IP,0:JP+1,KP),EW(IP,0:JP+1,KP)
      COMMON/VELOC2/UX(IP,0:JP+1,KP),    VY(IP,0:JP+1,KP)
      COMMON/MATOTYU/MATOA,MATOB,MATOC,MATOD
C
      DO 300 K=1,NZ1
      DO 300 J=1,NTH1
      DO 300 I=1,NR1
      WRITE(45,60)
     &U(I,J,K),V(I,J,K),W(I,J,K),
     &T(I,J,K),P(I,J,K)
   60 FORMAT(5D25.15)
  300 CONTINUE
C
      CALL TECOUTD
      RETURN
      END
C
CMMMMMMMMMMMMMMMMMMMMMMMMMMMMMMMMMMMMMMMMMMMMMMMMMMMMMMMMMMMMM
MMMMMMMMMMMMMMMMMMMMMC
C*****************************************************************C
      SUBROUTINE TECOUT
C*****************************************************************C
      IMPLICIT DOUBLE PRECISION(A-H,O-Z)
      PARAMETER(IP=51,JP=81,KP=70)
      COMMON/VELOC/U(IP,0:JP+1,KP),    V(IP,0:JP+1,KP),    W(IP,0:JP+1,KP)
     &                   ,U2(IP,0:JP+1,KP) ,V2(IP,0:JP+1,KP) ,W2(IP,0:JP+1,KP)
     &                   ,U3(IP,0:JP+1,KP) ,V3(IP,0:JP+1,KP) ,W3(IP,0:JP+1,KP)
     &                   ,V4(IP,0:JP+1,KP)
      COMMON/PRESS/    P(IP,0:JP+1,KP)
      COMMON/TEMP/      T(IP,0:JP+1,KP),TN(IP,0:JP+1,KP),TCO(IP,0:JP+1,KP)
      COMMON/DIMLSS/    RA,PR,RER,REC,GRA
      COMMON/ITPMT/    OMEGA,EPS
      COMMON/RGRID/      R(IP),RD(IP),DR,DRC(IP),HR
      COMMON/THGRID/    TH(JP),THD(JP),DTH,DTHC(JP),HTH
      COMMON/XGRID/      X(IP),XD(IP),DX,DXC(IP),HX
      COMMON/YGRID/      Y(JP),YD(JP),DY,DYC(JP),HY
      COMMON/ZGRID/      Z(KP),ZD(KP),DZ,DZC(KP),HZ
      COMMON/NODE/ NR,NR1,NTH,NTH1,NX,NX1,NY,NY1,NYC,NZ,NZ1,NWR,NWZ,NTHC
      COMMON/VELOC2/UX(IP,0:JP+1,KP),    VY(IP,0:JP+1,KP)
      COMMON/KOUBAI/    XX(IP-1,JP-1), YY(IP-1,JP-1)
```

```
      COMMON/MATOTYU/MATOA,MATOB,MATOC,MATOD
      COMMON/REFORM/ TT3(IP,0:JP+1,KP),TZ(IP,0:JP+1,KP)
     &              ,PPSI3(IP,0:JP+1,KP),PSIZ(IP,0:JP+1,KP)
     &              ,RR(IP)
C
C------------------   REFORMING BOUNDARY VALUE (TEMP.) -----------------C
      DO 100 J=1,NTH1
C
      DO 110 I=1,NR1
      TZ(I,J, 1 )=(T(I,J, 1 )+T(I,J, 2))*0.5D0
      DO 120 K=2,NZ1
      TZ(I,J, K )=T(I,J,K)
  120 CONTINUE
      TZ(I,J,NZ1)=(T(I,J,NZ1)+T(I,J,NZ))*0.5D0
  110 CONTINUE
C
      DO 150 K=1,NZ1
      TT3(1,J,K)=(TZ(1,J,K)+TZ(2,J,K))*0.5D0
      DO 155 I=2,NR+1
      TT3(I,J,K)=TZ(I,J,K)
  155 CONTINUE
      TT3(NR1,J,K)=(TZ(NR1,J,K)+TZ(NR,J,K))*0.5D0
  150 CONTINUE
C
  100 CONTINUE
C
      CALL TEC2
C
C---------------R GRID-----------------C
      RR(1)=RD(1)
      DO 310 I=2,NR
      RR(I)=R(I)
  310 CONTINUE
      RR(NR+1)=RR(NR)+DR*0.5D0
      RR(NR1)=RD(NR)
C
      DO 10 I=1,NR1
      DO 10 J=1,NTH
      XX(I,J)=RR(I)*DCOS(TH(J))
      YY(I,J)=RR(I)*DSIN(TH(J))
   10 CONTINUE
C
C--------------Z GRID------------------C
C
      DO 30 I=1,NR1
      DO 30 J=1,NTH
      DO 30 K=1,NZ1
      UX(I,J,K)=U3(I,J,K)*DCOS(TH(J))-V4(I,J,K)*DSIN(TH(J))
      VY(I,J,K)=U3(I,J,K)*DSIN(TH(J))+V4(I,J,K)*DCOS(TH(J))
   30 CONTINUE
C
      WRITE(51,*) 'variables = X,Y,Z,UX,VY,WZ,T,P'
      WRITE(51,156)NR1,NTH,NZ1
  156 FORMAT('zone i=',I5,'j=',I5,'k=',I5)
C
      DO 90 J=1,NTH
      DO 90 K=1,NZ1
        UX(1,J,K)=0.0D0
```

```
            VY(1,J,K)=0.0D0
            W3(1,J,K)=0.0D0
   90 CONTINUE
C
      DO 400 K=1,NZ1
      DO 400 J=1,NTH
      DO 400 I=1,NR1
      WRITE(51,160) XX(I,J),YY(I,J),Z(K),UX(I,J,K),
     &VY(I,J,K),W3(I,J,K),
     &TT3(I,J,K),P(I,J,K)
  160 FORMAT(8D25.15)
  400 CONTINUE
C
      RETURN
      END
C
C*******************************************************C
      SUBROUTINE TEC2
C*******************************************************C
      IMPLICIT DOUBLE PRECISION(A-H,O-Z)
      PARAMETER(IP=51,JP=81,KP=70)
      COMMON/NODE/ NR,NR1,NTH,NTH1,NX,NX1,NY,NY1,NYC,NZ,NZ1,NWR,NWZ,NTHC
      COMMON/VELOC/U(IP,0:JP+1,KP),    V(IP,0:JP+1,KP),    W(IP,0:JP+1,KP)
     &                 ,U2(IP,0:JP+1,KP) ,V2(IP,0:JP+1,KP) ,W2(IP,0:JP+1,KP)
     &                 ,U3(IP,0:JP+1,KP) ,V3(IP,0:JP+1,KP) ,W3(IP,0:JP+1,KP)
     &                 ,V4(IP,0:JP+1,KP)
      COMMON/MATOTYU/MATOA,MATOB,MATOC,MATOD
C
      DO 5010 I=1,NR1
      DO 5010 K=1,NZ1
         U(I,NTH1 ,K)=U(I, 2,K)
         U(I,NTH    ,K)=U(I, 1,K)
         V(I,NTH1 ,K)=V(I, 2,K)
         V(I,NTH    ,K)=V(I, 1,K)
         W(I,NTH1 ,K)=W(I, 2,K)
         W(I,NTH    ,K)=W(I, 1,K)
C          P(I,NTH1,K)=P(I, 2,K)
C          P(I, 1 ,K)=P(I,NTH,K)
 5010 CONTINUE
C----------------R component------------------C
C----------------- U -> U2 -------------------C
      DO 100 J=1,NTH1
      DO 110 I=1,NR
      U2(I,J, 1 )=(U(I,J, 1 )+U(I,J, 2))*0.5D0
      DO 120 K=2,NZ
      U2(I,J, K )=U(I,J,K)
  120 CONTINUE
      U2(I,J,NZ1)=(U(I,J,NZ1)+U(I,J,NZ))*0.5D0
  110 CONTINUE
C----------------- U2 -> U3 ------------------C
      DO 150 K=1,NZ1
      U3(1,J,K)=U2(1,J,K)
      DO 155 I=2,NR
      U3(I,J,K)=(U2(I-1,J,K)+U2(I,J,K))*0.5D0
  155 CONTINUE
      U3(NR1,J,K)=U2(NR,J,K)
  150 CONTINUE
C
```

```
      DO 170 I=1,NR1
      U3(I,J,1)=0.0D0
      U3(I,J,NZ1)=0.0D0
  170 CONTINUE
      DO 180 K=1,NZ1
      U3(NR1,J,K)=0.0D0
  180 CONTINUE
  100 CONTINUE
C
C----------------THETA component-------------------C
C----------------- V -> V2 -------------------C
      DO 300 J=1,NTH1
      DO 310 I=1,NR
      V2(I,J, 1 )=(V(I,J, 1 )+V(I,J, 2))*0.5D0
      DO 320 K=2,NZ1
      V2(I,J, K )=V(I,J,K)
  320 CONTINUE
      V2(I,J,NZ1)=(V(I,J,NZ1)+V(I,J,NZ))*0.5D0
  310 CONTINUE
C----------------- V2 -> V3 ------------------C
      DO 350 K=1,NZ1
      V3(1,J,K)=(V2(1,J,K)+V2(2,J,K))*0.5D0
      DO 355 I=2,NR1
      V3(I,J,K)=V2(I,J,K)
  355 CONTINUE
      V3(NR1,J,K)=(V2(NR1,J,K)+V2(NR,J,K))*0.5D0
  350 CONTINUE
C
      DO 370 I=1,NR1
      V3(I,J,1)=0.0D0
      V3(I,J,NZ1)=0.0D0
  370 CONTINUE
      DO 380 K=1,NZ1
      V3(NR1,J,K)=0.0D0
  380 CONTINUE
  300 CONTINUE
C----------------- V3 -> V4 ------------------C
      DO 231 K=1,NZ1
      DO 231 I=1,NR1
      V4(I,1,K)=(V3(I,1,K)+V3(I,NTH-1,K))*0.5D0
  231 CONTINUE
C
      DO 240 I=1,NR1
      DO 240 J=2,NTH
      DO 240 K=1,NZ1
      V4(I,J,K)=(V3(I,J-1,K)+V3(I,J,K))*0.5D0
  240 CONTINUE
C
C----------------- Z component-------------------C
C----------------- W -> W2 -------------------C
      DO 200 J=1,NTH1
      DO 210 K=1,NZ
      W2(1,J, K )=(W(1,J, K )+W(2,J, K))*0.5D0
      DO 220 I=2,NR
      W2(I,J, K )=W(I,J,K)
  220 CONTINUE
      W2(NR1,J,K)=(W(NR1,J,K)+W(NR,J,K))*0.5D0
  210 CONTINUE
```

```
C----------------- W2 -> W3 ------------------C
      DO 250 I=1,NR1
      W3(I,J,1)=W2(I,J,1)
      DO 255 K=2,NZ
      W3(I,J,K)=(W2(I,J,K)+W2(I,J,K-1))*0.5D0
  255 CONTINUE
      W3(I,J,NZ1)=W2(I,J,NZ1)
  250 CONTINUE
C
      DO 270 I=1,NR1
      W3(I,J,1)=0.0D0
      W3(I,J,NZ1)=0.0D0
  270 CONTINUE
      DO 280 K=1,NZ1
      W3(NR1,J,K)=0.0D0
  280 CONTINUE
  200 CONTINUE
      RETURN
      END
C
C*****************************************************************C
      SUBROUTINE TECOUTA
C*****************************************************************C
      IMPLICIT DOUBLE PRECISION(A-H,O-Z)
      PARAMETER(IP=51,JP=81,KP=70)
      COMMON/VELOC/U(IP,0:JP+1,KP),    V(IP,0:JP+1,KP),    W(IP,0:JP+1,KP)
     &                 ,U2(IP,0:JP+1,KP) ,V2(IP,0:JP+1,KP) ,W2(IP,0:JP+1,KP)
     &                 ,U3(IP,0:JP+1,KP) ,V3(IP,0:JP+1,KP) ,W3(IP,0:JP+1,KP)
     &                 ,V4(IP,0:JP+1,KP)
      COMMON/PRESS/    P(IP,0:JP+1,KP)
      COMMON/TEMP/      T(IP,0:JP+1,KP),TN(IP,0:JP+1,KP),TCO(IP,0:JP+1,KP)
      COMMON/DIMLSS/    RA,PR,RER,REC,GRA
      COMMON/ITPMT/    OMEGA,EPS
      COMMON/RGRID/      R(IP),RD(IP),DR,DRC(IP),HR
      COMMON/THGRID/    TH(JP),THD(JP),DTH,DTHC(JP),HTH
      COMMON/XGRID/      X(IP),XD(IP),DX,DXC(IP),HX
      COMMON/YGRID/      Y(JP),YD(JP),DY,DYC(JP),HY
      COMMON/ZGRID/      Z(KP),ZD(KP),DZ,DZC(KP),HZ
      COMMON/NODE/ NR,NR1,NTH,NTH1,NX,NX1,NY,NY1,NYC,NZ,NZ1,NWR,NWZ,NTHC
      COMMON/VELOC2/UX(IP,0:JP+1,KP),    VY(IP,0:JP+1,KP)
      COMMON/KOUBAI/    XX(IP-1,JP-1), YY(IP-1,JP-1)
      COMMON/MATOTYU/MATOA,MATOB,MATOC,MATOD
      COMMON/REFORM/ TT3(IP,0:JP+1,KP),TZ(IP,0:JP+1,KP)
     &                    ,PPSI3(IP,0:JP+1,KP),PSIZ(IP,0:JP+1,KP)
     &                    ,RR(IP)
C
C------------------    REFORMING BOUNDARY VALUE (TEMP.) -----------------C
      DO 100 J=1,NTH1
C
      DO 110 I=1,NR1
      TZ(I,J, 1 )=(T(I,J, 1 )+T(I,J, 2))*0.5D0
      DO 120 K=2,NZ1
      TZ(I,J, K )=T(I,J,K)
  120 CONTINUE
      TZ(I,J,NZ1)=(T(I,J,NZ1)+T(I,J,NZ))*0.5D0
  110 CONTINUE
C
      DO 150 K=1,NZ1
```

```
      TT3(1,J,K)=(TZ(1,J,K)+TZ(2,J,K))*0.5D0
      DO 155 I=2,NR+1
      TT3(I,J,K)=TZ(I,J,K)
  155 CONTINUE
      TT3(NR1,J,K)=(TZ(NR1,J,K)+TZ(NR,J,K))*0.5D0
  150 CONTINUE
C
  100 CONTINUE
C
C
      CALL TEC2
C
C---------------R GRID-----------------C
      RR(1)=RD(1)
      DO 310 I=2,NR
      RR(I)=R(I)
  310 CONTINUE
      RR(NR+1)=RR(NR)+DR*0.5D0
      RR(NR1)=RD(NR)
C
      DO 10 I=1,NR1
      DO 10 J=1,NTH
      XX(I,J)=RR(I)*DCOS(TH(J))
      YY(I,J)=RR(I)*DSIN(TH(J))
   10 CONTINUE
C
C--------------Z GRID------------------C
C
      DO 30 I=1,NR1
      DO 30 J=1,NTH
      DO 30 K=1,NZ1
      UX(I,J,K)=U3(I,J,K)*DCOS(TH(J))-V4(I,J,K)*DSIN(TH(J))
      VY(I,J,K)=U3(I,J,K)*DSIN(TH(J))+V4(I,J,K)*DCOS(TH(J))
   30 CONTINUE
C
      WRITE(52,*) 'variables = X,Y,Z,UX,VY,WZ,T,P'
      WRITE(52,156)NR1,NTH,NZ1
  156 FORMAT('zone i=',I5,'j=',I5,'k=',I5)
C
      DO 90 J=1,NTH
      DO 90 K=1,NZ1
        UX(1,J,K)=0.0D0
        VY(1,J,K)=0.0D0
        W3(1,J,K)=0.0D0
   90 CONTINUE
C
      DO 400 K=1,NZ1
      DO 400 J=1,NTH
      DO 400 I=1,NR1
      WRITE(52,160) XX(I,J),YY(I,J),Z(K),UX(I,J,K),
     &VY(I,J,K),W3(I,J,K),
     &TT3(I,J,K),P(I,J,K)
  160 FORMAT(8D25.15)
  400 CONTINUE
C
      RETURN
      END
C*****************************************************************C
```

```
      SUBROUTINE TECOUTB
C*****************************************************************C
      IMPLICIT DOUBLE PRECISION(A-H,O-Z)
      PARAMETER(IP=51,JP=81,KP=70)
      COMMON/VELOC/U(IP,0:JP+1,KP),    V(IP,0:JP+1,KP),    W(IP,0:JP+1,KP)
     &              ,U2(IP,0:JP+1,KP) ,V2(IP,0:JP+1,KP) ,W2(IP,0:JP+1,KP)
     &              ,U3(IP,0:JP+1,KP) ,V3(IP,0:JP+1,KP) ,W3(IP,0:JP+1,KP)
     &              ,V4(IP,0:JP+1,KP)
      COMMON/PRESS/    P(IP,0:JP+1,KP)
      COMMON/TEMP/      T(IP,0:JP+1,KP),TN(IP,0:JP+1,KP),TCO(IP,0:JP+1,KP)
      COMMON/DIMLSS/    RA,PR,RER,REC,GRA
      COMMON/ITPMT/    OMEGA,EPS
      COMMON/RGRID/      R(IP),RD(IP),DR,DRC(IP),HR
      COMMON/THGRID/    TH(JP),THD(JP),DTH,DTHC(JP),HTH
      COMMON/XGRID/      X(IP),XD(IP),DX,DXC(IP),HX
      COMMON/YGRID/      Y(JP),YD(JP),DY,DYC(JP),HY
      COMMON/ZGRID/      Z(KP),ZD(KP),DZ,DZC(KP),HZ
      COMMON/NODE/ NR,NR1,NTH,NTH1,NX,NX1,NY,NY1,NYC,NZ,NZ1,NWR,NWZ,NTHC
      COMMON/VELOC2/UX(IP,0:JP+1,KP),    VY(IP,0:JP+1,KP)
      COMMON/KOUBAI/    XX(IP-1,JP-1), YY(IP-1,JP-1)
      COMMON/MATOTYU/MATOA,MATOB,MATOC,MATOD
      COMMON/REFORM/ TT3(IP,0:JP+1,KP),TZ(IP,0:JP+1,KP)
     &                ,PPSI3(IP,0:JP+1,KP),PSIZ(IP,0:JP+1,KP)
     &                ,RR(IP)
C
C------------------    REFORMING BOUNDARY VALUE (TEMP.) -----------------C
      DO 100 J=1,NTH1
C
      DO 110 I=1,NR1
      TZ(I,J, 1 )=(T(I,J, 1 )+T(I,J, 2))*0.5D0
      DO 120 K=2,NZ1
      TZ(I,J, K )=T(I,J,K)
  120 CONTINUE
      TZ(I,J,NZ1)=(T(I,J,NZ1)+T(I,J,NZ))*0.5D0
  110 CONTINUE
C
      DO 150 K=1,NZ1
      TT3(1,J,K)=(TZ(1,J,K)+TZ(2,J,K))*0.5D0
      DO 155 I=2,NR+1
      TT3(I,J,K)=TZ(I,J,K)
  155 CONTINUE
      TT3(NR1,J,K)=(TZ(NR1,J,K)+TZ(NR,J,K))*0.5D0
  150 CONTINUE
C
  100 CONTINUE
C
      CALL TEC2
C
C---------------R GRID-----------------C
      RR(1)=RD(1)
      DO 310 I=2,NR
      RR(I)=R(I)
  310 CONTINUE
      RR(NR+1)=RR(NR)+DR*0.5D0
      RR(NR1)=RD(NR)
C
      DO 10 I=1,NR1
      DO 10 J=1,NTH
```

```
          XX(I,J)=RR(I)*DCOS(TH(J))
          YY(I,J)=RR(I)*DSIN(TH(J))
   10 CONTINUE
C--------------Z GRID------------------C
C
          DO 30 I=1,NR1
          DO 30 J=1,NTH
          DO 30 K=1,NZ1
          UX(I,J,K)=U3(I,J,K)*DCOS(TH(J))-V4(I,J,K)*DSIN(TH(J))
          VY(I,J,K)=U3(I,J,K)*DSIN(TH(J))+V4(I,J,K)*DCOS(TH(J))
   30 CONTINUE
          WRITE(53,*) 'variables = X,Y,Z,UX,VY,WZ,T,P'
          WRITE(53,156)NR1,NTH,NZ1
  156 FORMAT('zone i=',I5,'j=',I5,'k=',I5)
C
          DO 90 J=1,NTH
          DO 90 K=1,NZ1
            UX(1,J,K)=0.0D0
            VY(1,J,K)=0.0D0
            W3(1,J,K)=0.0D0
   90 CONTINUE
C
          DO 400 K=1,NZ1
          DO 400 J=1,NTH
          DO 400 I=1,NR1
          WRITE(53,160) XX(I,J),YY(I,J),Z(K),UX(I,J,K),
         &VY(I,J,K),W3(I,J,K),
         &TT3(I,J,K),P(I,J,K)
  160 FORMAT(8D25.15)
  400 CONTINUE
C
          RETURN
          END
C******************************************************************C
          SUBROUTINE TECOUTC
C******************************************************************C
          IMPLICIT DOUBLE PRECISION(A-H,O-Z)
          PARAMETER(IP=51,JP=81,KP=70)
          COMMON/VELOC/U(IP,0:JP+1,KP),    V(IP,0:JP+1,KP),    W(IP,0:JP+1,KP)
         &                    ,U2(IP,0:JP+1,KP) ,V2(IP,0:JP+1,KP) ,W2(IP,0:JP+1,KP)
         &                    ,U3(IP,0:JP+1,KP) ,V3(IP,0:JP+1,KP) ,W3(IP,0:JP+1,KP)
         &                    ,V4(IP,0:JP+1,KP)
          COMMON/PRESS/    P(IP,0:JP+1,KP)
          COMMON/TEMP/      T(IP,0:JP+1,KP),TN(IP,0:JP+1,KP),TCO(IP,0:JP+1,KP)
          COMMON/DIMLSS/    RA,PR,RER,REC,GRA
          COMMON/ITPMT/    OMEGA,EPS
          COMMON/RGRID/      R(IP),RD(IP),DR,DRC(IP),HR
          COMMON/THGRID/    TH(JP),THD(JP),DTH,DTHC(JP),HTH
          COMMON/XGRID/      X(IP),XD(IP),DX,DXC(IP),HX
          COMMON/YGRID/      Y(JP),YD(JP),DY,DYC(JP),HY
          COMMON/ZGRID/      Z(KP),ZD(KP),DZ,DZC(KP),HZ
          COMMON/NODE/ NR,NR1,NTH,NTH1,NX,NX1,NY,NY1,NYC,NZ,NZ1,NWR,NWZ,NTHC
          COMMON/VELOC2/UX(IP,0:JP+1,KP),    VY(IP,0:JP+1,KP)
          COMMON/KOUBAI/    XX(IP-1,JP-1), YY(IP-1,JP-1)
          COMMON/MATOTYU/MATOA,MATOB,MATOC,MATOD
          COMMON/REFORM/ TT3(IP,0:JP+1,KP),TZ(IP,0:JP+1,KP)
         &                        ,PPSI3(IP,0:JP+1,KP),PSIZ(IP,0:JP+1,KP)
         &                        ,RR(IP)
```

```
C
C------------------    REFORMING BOUNDARY VALUE (TEMP.) -----------------C
          DO 100 J=1,NTH1
C
          DO 110 I=1,NR1
          TZ(I,J, 1 )=(T(I,J, 1 )+T(I,J, 2))*0.5D0
          DO 120 K=2,NZ1
          TZ(I,J, K )=T(I,J,K)
   120 CONTINUE
          TZ(I,J,NZ1)=(T(I,J,NZ1)+T(I,J,NZ))*0.5D0
   110 CONTINUE
C
          DO 150 K=1,NZ1
          TT3(1,J,K)=(TZ(1,J,K)+TZ(2,J,K))*0.5D0
          DO 155 I=2,NR+1
          TT3(I,J,K)=TZ(I,J,K)
   155 CONTINUE
          TT3(NR1,J,K)=(TZ(NR1,J,K)+TZ(NR,J,K))*0.5D0
   150 CONTINUE
C
   100 CONTINUE
C
          CALL TEC2
C
C---------------R GRID-----------------C
          RR(1)=RD(1)
          DO 310 I=2,NR
          RR(I)=R(I)
   310 CONTINUE
          RR(NR+1)=RR(NR)+DR*0.5D0
          RR(NR1)=RD(NR)
C
          DO 10 I=1,NR1
          DO 10 J=1,NTH
          XX(I,J)=RR(I)*DCOS(TH(J))
          YY(I,J)=RR(I)*DSIN(TH(J))
     10 CONTINUE
C--------------Z GRID------------------C
C
          DO 30 I=1,NR1
          DO 30 J=1,NTH
          DO 30 K=1,NZ1
          UX(I,J,K)=U3(I,J,K)*DCOS(TH(J))-V4(I,J,K)*DSIN(TH(J))
          VY(I,J,K)=U3(I,J,K)*DSIN(TH(J))+V4(I,J,K)*DCOS(TH(J))
     30 CONTINUE
          WRITE(54,*) 'variables = X,Y,Z,UX,VY,WZ,T,P'
          WRITE(54,156)NR1,NTH,NZ1
   156 FORMAT('zone i=',I5,'j=',I5,'k=',I5)
C
          DO 90 J=1,NTH
          DO 90 K=1,NZ1
             UX(1,J,K)=0.0D0
             VY(1,J,K)=0.0D0
             W3(1,J,K)=0.0D0
     90 CONTINUE
C
          DO 400 K=1,NZ1
          DO 400 J=1,NTH
```

```
      DO 400 I=1,NR1
      WRITE(54,160) XX(I,J),YY(I,J),Z(K),UX(I,J,K),
     &VY(I,J,K),W3(I,J,K),
     &TT3(I,J,K),P(I,J,K)
  160 FORMAT(8D25.15)
  400 CONTINUE
C
      RETURN
      END
C******************************************************************C
      SUBROUTINE TECOUTD
C******************************************************************C
      IMPLICIT DOUBLE PRECISION(A-H,O-Z)
      PARAMETER(IP=51,JP=81,KP=70)
      COMMON/VELOC/U(IP,0:JP+1,KP),   V(IP,0:JP+1,KP),   W(IP,0:JP+1,KP)
     &            ,U2(IP,0:JP+1,KP) ,V2(IP,0:JP+1,KP) ,W2(IP,0:JP+1,KP)
     &            ,U3(IP,0:JP+1,KP) ,V3(IP,0:JP+1,KP) ,W3(IP,0:JP+1,KP)
     &            ,V4(IP,0:JP+1,KP)
      COMMON/PRESS/   P(IP,0:JP+1,KP)
      COMMON/TEMP/     T(IP,0:JP+1,KP),TN(IP,0:JP+1,KP),TCO(IP,0:JP+1,KP)
      COMMON/DIMLSS/   RA,PR,RER,REC,GRA
      COMMON/ITPMT/   OMEGA,EPS
      COMMON/RGRID/     R(IP),RD(IP),DR,DRC(IP),HR
      COMMON/THGRID/   TH(JP),THD(JP),DTH,DTHC(JP),HTH
      COMMON/XGRID/     X(IP),XD(IP),DX,DXC(IP),HX
      COMMON/YGRID/     Y(JP),YD(JP),DY,DYC(JP),HY
      COMMON/ZGRID/     Z(KP),ZD(KP),DZ,DZC(KP),HZ
      COMMON/NODE/ NR,NR1,NTH,NTH1,NX,NX1,NY,NY1,NYC,NZ,NZ1,NWR,NWZ,NTHC
      COMMON/VELOC2/UX(IP,0:JP+1,KP),   VY(IP,0:JP+1,KP)
      COMMON/KOUBAI/   XX(IP-1,JP-1), YY(IP-1,JP-1)
      COMMON/MATOTYU/MATOA,MATOB,MATOC,MATOD
      COMMON/REFORM/ TT3(IP,0:JP+1,KP),TZ(IP,0:JP+1,KP)
     &               ,PPSI3(IP,0:JP+1,KP),PSIZ(IP,0:JP+1,KP)
     &               ,RR(IP)
C
C-----------------   REFORMING BOUNDARY VALUE (TEMP.) ----------------C
      DO 100 J=1,NTH1
C
      DO 110 I=1,NR1
      TZ(I,J, 1 )=(T(I,J, 1 )+T(I,J, 2))*0.5D0
      DO 120 K=2,NZ1
      TZ(I,J, K )=T(I,J,K)
  120 CONTINUE
      TZ(I,J,NZ1)=(T(I,J,NZ1)+T(I,J,NZ))*0.5D0
  110 CONTINUE
C
      DO 150 K=1,NZ1
      TT3(1,J,K)=(TZ(1,J,K)+TZ(2,J,K))*0.5D0
      DO 155 I=2,NR+1
      TT3(I,J,K)=TZ(I,J,K)
  155 CONTINUE
      TT3(NR1,J,K)=(TZ(NR1,J,K)+TZ(NR,J,K))*0.5D0
  150 CONTINUE
C
  100 CONTINUE
C
      CALL TEC2
C
```

```
C---------------R GRID-----------------C
      RR(1)=RD(1)
      DO 310 I=2,NR
      RR(I)=R(I)
  310 CONTINUE
      RR(NR+1)=RR(NR)+DR*0.5D0
      RR(NR1)=RD(NR)
C
      DO 10 I=1,NR1
      DO 10 J=1,NTH
      XX(I,J)=RR(I)*DCOS(TH(J))
      YY(I,J)=RR(I)*DSIN(TH(J))
   10 CONTINUE
C--------------Z GRID------------------C
C
      DO 30 I=1,NR1
      DO 30 J=1,NTH
      DO 30 K=1,NZ1
      UX(I,J,K)=U3(I,J,K)*DCOS(TH(J))-V4(I,J,K)*DSIN(TH(J))
      VY(I,J,K)=U3(I,J,K)*DSIN(TH(J))+V4(I,J,K)*DCOS(TH(J))
   30 CONTINUE
      WRITE(55,*) 'variables = X,Y,Z,UX,VY,WZ,T,P'
      WRITE(55,156)NR1,NTH,NZ1
  156 FORMAT('zone i=',I5,'j=',I5,'k=',I5)
C
      DO 90 J=1,NTH
      DO 90 K=1,NZ1
        UX(1,J,K)=0.0D0
        VY(1,J,K)=0.0D0
        W3(1,J,K)=0.0D0
   90 CONTINUE
C
      DO 400 K=1,NZ1
      DO 400 J=1,NTH
      DO 400 I=1,NR1
      WRITE(55,160) XX(I,J),YY(I,J),Z(K),UX(I,J,K),
     &VY(I,J,K),W3(I,J,K),
     &TT3(I,J,K),P(I,J,K)
  160 FORMAT(8D25.15)
  400 CONTINUE
C
      RETURN
      END
C!!!!!!!!!!!!!!!!!!!!!!!!!!!!!!!!!!!!!!!!!!!!!!!!!!!!!!!!!!!!!!!!!!!!C
CMMMMMMMMMMMMMMMMMMMMMMMMMMMMMMMMMMMMMMMMMMMMMMMMMM
MMMMMMMMMMMMMC
CiiiiiiiiiiiiiiiiiiiiiiiiiiiiiiiiiiiiiiiiiiiiiiiiiiiiiiiiiiiiiiiiiiiiiC
C********************************************************************C
      SUBROUTINE COMUVW
C********************************************************************C
      IMPLICIT DOUBLE PRECISION(A-H,O-Z)
      PARAMETER(IP=51,JP=81,KP=70)
      INTEGER ICYL
      COMMON/VELOC/U(IP,0:JP+1,KP),    V(IP,0:JP+1,KP),    W(IP,0:JP+1,KP)
     &                        ,U2(IP,0:JP+1,KP) ,V2(IP,0:JP+1,KP) ,W2(IP,0:JP+1,KP)
     &                        ,U3(IP,0:JP+1,KP) ,V3(IP,0:JP+1,KP) ,W3(IP,0:JP+1,KP)
     &                        ,V4(IP,0:JP+1,KP)
      COMMON/VELOCN/ UN(IP,0:JP+1,KP),VN(IP,0:JP+1,KP),WN(IP,0:JP+1,KP)
```

```
      COMMON/PRESS/    P(IP,0:JP+1,KP)
      COMMON/TEMP/      T(IP,0:JP+1,KP),TN(IP,0:JP+1,KP),TCO(IP,0:JP+1,KP)
      COMMON/MAPMT/    IDX,MAXIT
      COMMON/TIME/      DT
      COMMON/RGRID/      R(IP),RD(IP),DR,DRC(IP),HR
      COMMON/THGRID/    TH(JP),THD(JP),DTH,DTHC(JP),HTH
      COMMON/XGRID/      X(IP),XD(IP),DX,DXC(IP),HX
      COMMON/YGRID/      Y(JP),YD(JP),DY,DYC(JP),HY
      COMMON/ZGRID/      Z(KP),ZD(KP),DZ,DZC(KP),HZ
      COMMON/NODE/ NR,NR1,NTH,NTH1,NX,NX1,NY,NY1,NYC,NZ,NZ1,NWR,NWZ,NTHC
      COMMON/DIMLSS/    RA,PR,RER,REC,GRA
      COMMON/NAM/          NAME(10)
      COMMON/MGDSTY/    BX(IP-1,JP-1,KP-1), BY(IP-1,JP-1,KP-1),
     &                             BZ(IP-1,JP-1,KP-1),              BR(IP-1,JP-1,KP-1),
     &                             BTH(IP-1,JP-1,KP-1),
     &                             PRM,ALPHAB,BX0,BY0,BZ0,
     &                             G(IP,JP,KP)
c
      COMMON/JIKA/ GANMA
      COMMON/PAR2/        ICYL,ITER
      COMMON/FUNC/ZGETA,RGETA
      COMMON/PAR/          GX(IP,JP,KP),GY(IP,JP,KP),GZ(IP,JP,KP),
     &                             GTH(IP,JP,KP),GR(IP,JP,KP),
     &                             SX(IP,JP,KP),SY(IP,JP,KP),
     &                             SZ(IP,JP,KP),STH(IP,JP,KP),
     &                             UI,VI,WI,DELT,DT1,ADT,EPSI,OMG,
     &                             BETA,ALPHA,VELMX,
     &                             TWPRT,TWPLT,TWFIN,CWPRT,CWPLT,Cm
C
      COMMON/WARI/        DRIN,DZIN,DTHIN
      COMMON/LEAN/SLOPER,SLOPED,PAI
      COMMON/MAGLIQ/    CONST
      common/Teramoto/hosei
C
C-----------------------
C         U-VELOCITY
C-----------------------
      DO 1000 J=2,NTH
      DO 1000 K=2,NZ
      DO 1000 I=2,NR-1
C
          RDR=1.0D0/(RD(I)*DR)
          RDTH=1.0D0/(RD(I)*DTH)
C
          UE=(UN(I+1,J,K)+UN(I,J,K))*0.5D0
          UW=(UN(I,J,K)+UN(I-1,J,K))*0.5D0
          VA=(VN(I+1, J ,K)+VN(I, J ,K))*0.5D0
          VB=(VN(I+1,J-1,K)+VN(I,J-1,K))*0.5D0
          WD=(WN(I+1,J, K )+WN(I, J ,K))*0.5D0
          WU=(WN(I+1,J,K-1)+WN(I,J,K-1))*0.5D0
C
C**************** 3UPWIND METHOD QUICK**********************
C
      IF((I.EQ.2).OR.(I.EQ.NR-1)) THEN
C
          TJE=R(I+1)*(UE*(UN(I+1,J,K)+UN(I,J,K))*0.5D0
     &           -(PR+DABS(UE)*DR*0.5D0)*(UN(I+1,J,K)-UN(I,J,K))*DRIN)
          TJW=R(I)*(UW*(UN(I,J,K)+UN(I-1,J,K))*0.5D0
```

```
     &      -(PR+DABS(UW)*DR*0.5D0)*(UN(I,J,K)-UN(I-1,J,K))*DRIN)
C
      ELSE
        UEE=(-UN(I+2,J,K)+9.0D0*UN(I+1,J,K)
     &      +9.0D0*UN(I,J,K)-UN(I-1,J,K))*0.0625D0
        UWW=(-UN(I+1,J,K)+9.0D0*UN(I,J,K)
     &      +9.0D0*UN(I-1,J,K)-UN(I-2,J,K))*0.0625D0
        TJE=R(I+1)*(UE*UEE
     &      -DABS(UE)
     &      *(-UN(I+2,J,K)+3.0D0*UN(I+1,J,K)
     &      -3.0D0*UN(I,J,K)+UN(I-1,J,K))*0.0625D0
     &      -PR*(UN(I+1,J,K)-UN(I,J,K))*DRIN)
        TJW=R(I)*(UW*UWW
     &      -DABS(UW)
     &      *(-UN(I+1,J,K)+3.0D0*UN(I,J,K)
     &      -3.0D0*UN(I-1,J,K)+UN(I-2,J,K))*0.0625D0
     &      -PR*(UN(I,J,K)-UN(I-1,J,K))*DRIN)
      END IF
C
        TJA=VA*(-UN(I,J+2,K)+9.0D0*UN(I,J+1,K)
     &      +9.0D0*UN(I,J,K)-UN(I,J-1,K))*0.0625D0
     &      -DABS(VA)
     &      *(-UN(I,J+2,K)+3.0D0*UN(I,J+1,K)
     &      -3.0D0*UN(I,J,K)+UN(I,J-1,K))*0.0625D0
     &      -PR*(UN(I,J+1,K)-UN(I,J,K))*RDTH
        TJB=VB*(-UN(I,J+1,K)+9.0D0*UN(I,J,K)
     &      +9.0D0*UN(I,J-1,K)-UN(I,J-2,K))*0.0625D0
     &      -DABS(VB)
     &      *(-UN(I,J+1,K)+3.0D0*UN(I,J,K)
     &      -3.0D0*UN(I,J-1,K)+UN(I,J-2,K))*0.0625D0
     &      -PR*(UN(I,J,K)-UN(I,J-1,K))*RDTH
C
      IF((K.EQ.2).OR.(K.EQ.NZ)) THEN
C
        TJD=WD*(UN(I,J,K+1)+UN(I,J,K))*0.5D0
     &      -(PR+DABS(WD)*DZ*0.5D0)*(UN(I,J,K+1)-UN(I,J,K))*DZIN
        TJU=WU*(UN(I,J,K)+UN(I,J,K-1))*0.5D0
     &      -(PR+DABS(WU)*DZ*0.5D0)*(UN(I,J,K)-UN(I,J,K-1))*DZIN
C
      ELSE
        TJD=WD*(-UN(I,J,K+2)+9.0D0*UN(I,J,K+1)
     &      +9.0D0*UN(I,J,K)-UN(I,J,K-1))*0.0625D0
     &      -DABS(WD)
     &      *(-UN(I,J,K+2)+3.0D0*UN(I,J,K+1)
     &      -3.0D0*UN(I,J,K)+UN(I,J,K-1))*0.0625D0
     &      -PR*(UN(I,J,K+1)-UN(I,J,K))*DZIN
        TJU=WU*(-UN(I,J,K+1)+9.0D0*UN(I,J,K)
     &      +9.0D0*UN(I,J,K-1)-UN(I,J,K-2))*0.0625D0
     &      -DABS(WU)
     &      *(-UN(I,J,K+1)+3.0D0*UN(I,J,K)
     &      -3.0D0*UN(I,J,K-1)+UN(I,J,K-2))*0.0625D0
     &      -PR*(UN(I,J,K)-UN(I,J,K-1))*DZIN
      END IF
************************************************************************
        TJR=(TJE-TJW)*RDR
        TJTH=(TJA-TJB)*RDTH
        TJZ=(TJD-TJU)*DZIN
        SSU=(VN( I ,J-1,K)+VN( I ,J,K)
```

```
      &        +VN(I+1,J-1,K)+VN(I+1,J,K))**2/(RD(I)*16.0D0)
      &       -PR/RD(I)**2*(
      &              UN(I,J,K)
      &             +1.0D0/DTH*(VN(I, J ,K)+VN(I+1, J ,K)
      &                         -VN(I,J-1,K)-VN(I+1,J-1,K))
      &                        )
C--------------------------------------------------------------
           SSU1=0.5D0*(TN(I,J,K)+TN(I+1,J,K))
C--------------------------------------------------------------
c          SSWU=0.5D0*(TN(I,J,K)+TN(I,J,K+1))
C--------------------------------------------------------------
          U(I,J,K)=UN(I,J,K)
      &              +DT*(-TJR-TJTH-TJZ+SSU)
      &              -DT*DRIN*(P(I+1,J,K)-P(I,J,K))
C
      &            -DT*PR*PR*GRA*SSU1*DCOS(SLOPER)*DSIN(TH(J))
C
c   2014年12月24日改造
c    &                       -DT*GANMA*0.5D0*CONST*PR*RA*SSU1
      &                      -DT*GANMA*0.5D0*CONST*PR*RA*SSU1
      &                   *( (BR(I+1,J,K)**2-BR(I,J,K)**2)/DR
      &                     +(BZ(I+1,J,K)**2-BZ(I,J,K)**2)/DR
      &                     )
C
      &         +DT*GANMA*0.5D0*CONST*PR*RA*(BR(I,J,K)**2+BZ(I,J,K)**2)
      &         *(TN(I+1,J,K)-TN(I,J,K))/DR
      &
C
C--------------------------------------------------------------
 1000 CONTINUE
C
C---------------------------
C     V-VELOCITY
C---------------------------
       DO 2000 I=2,NR
       DO 2000 J=2,NTH
       DO 2000 K=2,NZ
C
          RDR=1.0D0/(R(I)*DR)
          RDTH=1.0D0/(R(I)*DTH)
C
          UE=(UN( I ,J+1,K)+UN( I ,J,K))*0.5D0
          UW=(UN(I-1,J+1,K)+UN(I-1,J,K))*0.5D0
          VA=(VN(I,J+1,K)+VN(I,J,K))*0.5D0
          VB=(VN(I,J,K)+VN(I,J-1,K))*0.5D0
          WD=(WN(I,J+1, K )+WN(I,J, K ))*0.5D0
          WU=(WN(I,J+1,K-1)+WN(I,J,K-1))*0.5D0
C
C**************** 3UPWIND METHOD QUICK**********************
C
       IF((I.EQ.2).OR.(I.EQ.NR)) THEN
C
          TJE=RD( I )*(UE*(VN(I+1,J,K)+VN(I,J,K))*0.5D0
      &       -(PR+DABS(UE)*DR*0.5D0)*(VN(I+1,J,K)-VN(I,J,K))*DRIN)
          TJW=RD(I-1)*(UW*(VN(I,J,K)+VN(I-1,J,K))*0.5D0
      &       -(PR+DABS(UW)*DR*0.5D0)*(VN(I,J,K)-VN(I-1,J,K))*DRIN)
C
       ELSE
```

```
            TJE=RD( I )*(UE*(-VN(I+2,J,K)+9.0D0*VN(I+1,J,K)
     &          +9.0D0*VN(I,J,K)-VN(I-1,J,K))*0.0625D0
     &          -DABS(UE)
     &          *(-VN(I+2,J,K)+3.0D0*VN(I+1,J,K)
     &          -3.0D0*VN(I,J,K)+VN(I-1,J,K))*0.0625D0
     &          -PR*(VN(I+1,J,K)-VN(I,J,K))*DRIN)
            TJW=RD(I-1)*(UW*(-VN(I+1,J,K)+9.0D0*VN(I,J,K)
     &          +9.0D0*VN(I-1,J,K)-VN(I-2,J,K))*0.0625D0
     &          -DABS(UW)
     &          *(-VN(I+1,J,K)+3.0D0*VN(I,J,K)
     &          -3.0D0*VN(I-1,J,K)+VN(I-2,J,K))*0.0625D0
     &          -PR*(VN(I,J,K)-VN(I-1,J,K))*DRIN)
         END IF
C
            VAA=(-VN(I,J+2,K)+9.0D0*VN(I,J+1,K)
     &          +9.0D0*VN(I,J,K)-VN(I,J-1,K))*0.0625D0
            VBB=(-VN(I,J+1,K)+9.0D0*VN(I,J,K)
     &          +9.0D0*VN(I,J-1,K)-VN(I,J-2,K))*0.0625D0
            TJA=VA*VAA
     &          -DABS(VA)
     &          *(-VN(I,J+2,K)+3.0D0*VN(I,J+1,K)
     &          -3.0D0*VN(I,J,K)+VN(I,J-1,K))*0.0625D0
     &          -PR*(VN(I,J+1,K)-VN(I,J,K))*RDTH
            TJB=VB*VBB
     &          -DABS(VB)
     &          *(-VN(I,J+1,K)+3.0D0*VN(I,J,K)
     &          -3.0D0*VN(I,J-1,K)+VN(I,J-2,K))*0.0625D0
     &          -PR*(VN(I,J,K)-VN(I,J-1,K))*RDTH
C
         IF((K.EQ.2).OR.(K.EQ.NZ)) THEN
C
            TJD=WD*(VN(I,J,K+1)+VN(I,J,K))*0.5D0
     &          -(PR+DABS(WD)*DZ*0.5D0)*(VN(I,J,K+1)-VN(I,J,K))*DZIN
            TJU=WU*(VN(I,J,K)+VN(I,J,K-1))*0.5D0
     &          -(PR+DABS(WU)*DZ*0.5D0)*(VN(I,J,K)-VN(I,J,K-1))*DZIN
C
         ELSE
            TJD=WD*(-VN(I,J,K+2)+9.0D0*VN(I,J,K+1)
     &          +9.0D0*VN(I,J,K)-VN(I,J,K-1))*0.0625D0
     &          -DABS(WD)
     &          *(-VN(I,J,K+2)+3.0D0*VN(I,J,K+1)
     &          -3.0D0*VN(I,J,K)+VN(I,J,K-1))*0.0625D0
     &          -PR*(VN(I,J,K+1)-VN(I,J,K))*DZIN
            TJU=WU*(-VN(I,J,K+1)+9.0D0*VN(I,J,K)
     &          +9.0D0*VN(I,J,K-1)-VN(I,J,K-2))*0.0625D0
     &          -DABS(WU)
     &          *(-VN(I,J,K+1)+3.0D0*VN(I,J,K)
     &          -3.0D0*VN(I,J,K-1)+VN(I,J,K-2))*0.0625D0
     &          -PR*(VN(I,J,K)-VN(I,J,K-1))*DZIN
         END IF
C
C*****************************************************************
            TJR=(TJE-TJW)*RDR
            TJTH=(TJA-TJB)*RDTH
            TJZ=(TJD-TJU)*DZIN
            SSV=-0.25D0*VN(I,J,K)/R(I)*(
     &                UN(I-1,J,K)+UN(I-1,J+1,K)+UN(I,J,K)+UN(I,J+1,K) )
     &           -PR/R(I)**2*(VN(I,J,K)
```

```
     &              -1.0D0/DTH*
     &                    (UN(I-1,J+1,K)+UN(I,J+1,K)-UN(I-1,J,K)-UN(I,J,K))
     &                    )
C-----------------------------------------------------------------
          SSWV=0.5D0*(TN(I,J+1,K)+TN(I,J,K))
C-----------------------------------------------------------------
C            V(I,J,K)=VN(I,J,K)+DT*(-TJR-TJTH-TJZ+SSV)
C     &                -DT/(R(I)*DTH)*(P(I,J+1,K)-P(I,J,K))
C
          V(I,J,K)=VN(I,J,K)
     &              +DT*(-TJR-TJTH-TJZ+SSV)
     &              -DT/(R(I)*DTH)*(P(I,J+1,K)-P(I,J,K))
C     &   -DT*PR*PR*GRA*SSWV*DCOS(SLOPER)*DCOS(TH(J)-0.5D0*DTH)
     &              -DT*PR*RA*SSWV
     &                *DCOS(SLOPER)*DCOS(TH(J)+0.5D0*DTH)
C
C-----------------------------------------------------------------
 2000 CONTINUE
C----------------------
C     W-VELOCITY
C----------------------
      DO 3000 I=2,NR
      DO 3000 J=2,NTH
      DO 3000 K=2,NZ-1
C
          RDR=1.0D0/(R(I)*DR)
          RDTH=1.0D0/(R(I)*DTH)
C
          UE=(UN( I ,J,K+1)+UN( I ,J,K))*0.5D0
          UW=(UN(I-1,J,K+1)+UN(I-1,J,K))*0.5D0
          VA=(VN(I, J ,K+1)+VN(I, J ,K))*0.5D0
          VB=(VN(I,J-1,K+1)+VN(I,J-1,K))*0.5D0
          WD=(WN(I,J,K+1)+WN(I,J,K))*0.5D0
          WU=(WN(I,J,K)+WN(I,J,K-1))*0.5D0
C
C***************** 3UPWIND METHOD QUICK***********************
C
      IF((I.EQ.2).OR.(I.EQ.NR)) THEN
C
          TJE=RD( I )*(
     &       UE*(WN(I+1,J,K)+WN(I,J,K))*0.5D0
     &         -(PR+DABS(UE)*DR*0.5D0)*(WN(I+1,J,K)-WN(I,J,K))*DRIN
     &                  )
C
          TJW=RD(I-1)*(
     &       UW*(WN(I,J,K)+WN(I-1,J,K))*0.5D0
     &         -(PR+DABS(UW)*DR*0.5D0)*(WN(I,J,K)-WN(I-1,J,K))*DRIN
     &                  )
C
      ELSE
          TJE=RD( I )*(
     &       UE*(-WN(I+2,J,K)+9.0D0*WN(I+1,J,K)
     &         +9.0D0*WN(I,J,K)-WN(I-1,J,K))*0.0625D0
     &         -DABS(UE)*(-WN(I+2,J,K)+3.0D0*WN(I+1,J,K)
     &         -3.0D0*WN(I,J,K)+WN(I-1,J,K))*0.0625D0
     &         -PR*(WN(I+1,J,K)-WN(I,J,K))*DRIN
     &                  )
C
```

```
            TJW=RD(I-1)*(
     &        UW*(-WN(I+1,J,K)+9.0D0*WN(I,J,K)
     &           +9.0D0*WN(I-1,J,K)-WN(I-2,J,K))*0.0625D0
     &           -DABS(UW)*(-WN(I+1,J,K)+3.0D0*WN(I,J,K)
     &           -3.0D0*WN(I-1,J,K)+WN(I-2,J,K))*0.0625D0
     &           -PR*(WN(I,J,K)-WN(I-1,J,K))*DRIN
     &                         )
        END IF
C
            TJA=VA*(
     &      -WN(I,J+2,K)+9.0D0*WN(I,J+1,K)
     &          +9.0D0*WN(I,J,K)-WN(I,J-1,K))*0.0625D0
     &          -DABS(VA)*(-WN(I,J+2,K)+3.0D0*WN(I,J+1,K)
     &          -3.0D0*WN(I,J,K)+WN(I,J-1,K))*0.0625D0
     &          -PR*(WN(I,J+1,K)-WN(I,J,K))*RDTH
C
            TJB=VB*(-WN(I,J+1,K)+9.0D0*WN(I,J,K)
     &          +9.0D0*WN(I,J-1,K)-WN(I,J-2,K))*0.0625D0
     &          -DABS(VB)*(-WN(I,J+1,K)+3.0D0*WN(I,J,K)
     &          -3.0D0*WN(I,J-1,K)+WN(I,J-2,K))*0.0625D0
     &          -PR*(WN(I,J,K)-WN(I,J-1,K))*RDTH
C
        IF((K.EQ.2).OR.(K.EQ.NZ-1)) THEN
C
            TJD=WD*(WN(I,J,K+1)+WN(I,J,K))*0.5D0
     &           -(PR+DABS(WD)*DZ*0.5D0)*(WN(I,J,K+1)-WN(I,J,K))*DZIN
C
            TJU=WU*(WN(I,J,K)+WN(I,J,K-1))*0.5D0
     &           -(PR+DABS(WU)*DZ*0.5D0)*(WN(I,J,K)-WN(I,J,K-1))*DZIN
C
        ELSE
            WDD=(-WN(I,J,K+2)+9.0D0*WN(I,J,K+1)
     &          +9.0D0*WN(I,J,K)-WN(I,J,K-1))*0.0625D0
C
            WUU=(-WN(I,J,K+1)+9.0D0*WN(I,J,K)
     &          +9.0D0*WN(I,J,K-1)-WN(I,J,K-2))*0.0625D0
C
            TJD=WD*WDD
     &          -DABS(WD)*(-WN(I,J,K+2)+3.0D0*WN(I,J,K+1)
     &          -3.0D0*WN(I,J,K)+WN(I,J,K-1))*0.0625D0
     &          -PR*(WN(I,J,K+1)-WN(I,J,K))*DZIN
C
            TJU=WU*WUU
     &          -DABS(WU)*(-WN(I,J,K+1)+3.0D0*WN(I,J,K)
     &          -3.0D0*WN(I,J,K-1)+WN(I,J,K-2))*0.0625D0
     &          -PR*(WN(I,J,K)-WN(I,J,K-1))*DZIN
        END IF
C
C*******************************************************************
            TJR=(TJE-TJW)*RDR
            TJTH=(TJA-TJB)*RDTH
            TJZ=(TJD-TJU)*DZIN
            SSWW=0.5D0*(TN(I,J,K)+TN(I,J,K+1))
C-------------------------------------------------------------------
            W(I,J,K)=WN(I,J,K)
     &                 +DT*(-TJR-TJTH-TJZ)
     &                 -DT*PR*PR*GRA*SSWW*DSIN(SLOPER)
     &                 -DT*DZIN*(P(I,J,K+1)-P(I,J,K))
```

```
C
c 磁気勾配項の補正をγに含めるため以前からある磁気力項を
c 容器中心とコイル中心間距離で補正している。
c この Hosei 項は SUBROUTINE INP で定義済み
c          Hosei=2.0D0*3.141592653D0*SQRT(ZGETA**2+RGETA**2)
c   2014 年 12 月 24 日改造
c        &                      -DT*GANMA*0.5D0*CONST*PR*RA*SSWW
        &                      -DT*GANMA*0.5D0*CONST*PR*RA*SSWW
        &                           *( (BR(I,J,K+1)**2-BR(I,J,K)**2)/DZ
        &                             +(BZ(I,J,K+1)**2-BZ(I,J,K)**2)/DZ)
C
        &              +DT*GANMA*0.5D0*CONST*PR*RA*(BR(I,J,K)**2+BZ(I,J,K)**2)
        &              *(TN(I,J,K+1)-TN(I,J,K))/DZ
C
C         ( (BR(I,J,K+1)**2-BR(I,J,K)**2)/DZ
C         &                              +(BZ(I,J,K+1)**2-BZ(I,J,K)**2)/DZ)
C
C         &                              -DT*GANMA*0.5D0*CONST*PR*RA
C         &                         *(SSU1/DR)*((BZ(I+1,J,K)**2+BZ(I,J,K)**2)
C
C------------------------------------------------------------------
 3000 CONTINUE
      CALL BND
      RETURN
      END
C
C*******************************************************************C
      SUBROUTINE BND
C*******************************************************************C
      IMPLICIT DOUBLE PRECISION(A-H,O-Z)
      PARAMETER(IP=51,JP=81,KP=70)
      COMMON/VELOC/U(IP,0:JP+1,KP),    V(IP,0:JP+1,KP),    W(IP,0:JP+1,KP)
     &                 ,U2(IP,0:JP+1,KP) ,V2(IP,0:JP+1,KP) ,W2(IP,0:JP+1,KP)
     &                 ,U3(IP,0:JP+1,KP) ,V3(IP,0:JP+1,KP) ,W3(IP,0:JP+1,KP)
     &                 ,V4(IP,0:JP+1,KP)
      COMMON/PRESS/    P(IP,0:JP+1,KP)
      COMMON/RGRID/      R(IP),RD(IP),DR,DRC(IP),HR
      COMMON/THGRID/    TH(JP),THD(JP),DTH,DTHC(JP),HTH
      COMMON/XGRID/      X(IP),XD(IP),DX,DXC(IP),HX
      COMMON/YGRID/      Y(JP),YD(JP),DY,DYC(JP),HY
      COMMON/ZGRID/      Z(KP),ZD(KP),DZ,DZC(KP),HZ
      COMMON/NODE/ NR,NR1,NTH,NTH1,NX,NX1,NY,NY1,NYC,NZ,NZ1,NWR,NWZ,NTHC
      COMMON/MGDSTY/    BX(IP-1,JP-1,KP-1), BY(IP-1,JP-1,KP-1),
     &                       BZ(IP-1,JP-1,KP-1),             BR(IP-1,JP-1,KP-1),
     &                       BTH(IP-1,JP-1,KP-1),
     &                       PRM,ALPHAB,BX0,BY0,BZ0,
     &                       G(IP,JP,KP)
      COMMON/LEAN/SLOPER,SLOPED,PAI
C
C-------------------------------------------------------------------C
C                                U-VELOCITY                          C
C-------------------------------------------------------------------C
C
C----- INTERFACE & FREE SURFACE & BOTTOM WALL & SIDE WALL -----C
C
      DO 1000 J=1,NTH1
         DO 1010 I=2,NR-1
            U(I,J,1)=-U(I,J,2)
```

```
 1010       CONTINUE
            DO 1030 I=2,NR
                U(I,J,NZ1)=-U(I,J,NZ)
 1030       CONTINUE
            DO 1040 K=1,NZ1
                U(NR,J,K)=0.0D0
 1040       CONTINUE
 1000 CONTINUE
        CALL UCNTER(U)
C-------------------------------------------------------------------C
C                          V-VELOCITY                              C
C-------------------------------------------------------------------C
C
C----- INTEFACE & FREE SURFACE & SIDE WALL & BOTTOM -----C
C
        DO 2000 J=1,NTH1
            DO 2010 I=2,NR
                V(I,J,1)=-V(I,J,2)
C
 2010       CONTINUE
            DO 2040 I=2,NR
                V(I,J,NZ1)=-V(I,J,NZ)
 2040       CONTINUE
            DO 2030 K=1,NZ1
                V(NR1,J,K)=-V(NR,J,K)
 2030       CONTINUE
 2000 CONTINUE
        CALL VCNTER(V)
C-------------------------------------------------------------------C
C                          W-VELOCITY                              C
C-------------------------------------------------------------------C
C
C----- TOP & BOTTOM & SIDE WALL -----C
C
        DO 3000 J=1,NTH1
            DO 3010 I=2,NR1
                W(I,J, 1)=0.0D0
                W(I,J,NZ)=0.0D0
 3010       CONTINUE
            DO 3020 K=1,NZ
                W(NR1,J,K)=-W(NR,J,K)
 3020       CONTINUE
 3000 CONTINUE
        CALL CENTER(W)
        CALL CENTER(P)
        DO 5000 I=1,NR1
        DO 5000 K=1,NZ1
            U(I,NTH1,K)=U(I, 2,K)
            U(I, 1 ,K)=U(I,NTH,K)
            U(I,NTH1+1,K)=U(I,3,K)
            U(I,0,K)=U(I,NTH-1,K)
            V(I,NTH1,K)=V(I, 2,K)
            V(I, 1 ,K)=V(I,NTH,K)
            V(I,NTH1+1,K)=V(I,3,K)
            V(I,0,K)=V(I,NTH-1,K)
            W(I,NTH1,K)=W(I, 2,K)
            W(I, 1 ,K)=W(I,NTH,K)
            W(I,NTH1+1,K)=W(I,3,K)
```

```
          W(I,0,K)=W(I,NTH-1,K)
          P(I,NTH1,K)=P(I, 2,K)
          P(I, 1 ,K)=P(I,NTH,K)
 5000 CONTINUE
cc
      RETURN
      END
C******************************************************************C
      SUBROUTINE TBND
C******************************************************************C
      IMPLICIT DOUBLE PRECISION(A-H,O-Z)
      PARAMETER(IP=51,JP=81,KP=70)
      COMMON/TEMP/    T(IP,0:JP+1,KP),TN(IP,0:JP+1,KP),TCO(IP,0:JP+1,KP)
      COMMON/RGRID/    R(IP),RD(IP),DR,DRC(IP),HR
      COMMON/THGRID/   TH(JP),THD(JP),DTH,DTHC(JP),HTH
      COMMON/XGRID/    X(IP),XD(IP),DX,DXC(IP),HX
      COMMON/YGRID/    Y(JP),YD(JP),DY,DYC(JP),HY
      COMMON/ZGRID/    Z(KP),ZD(KP),DZ,DZC(KP),HZ
      COMMON/NODE/ NR,NR1,NTH,NTH1,NX,NX1,NY,NY1,NYC,NZ,NZ1,NWR,NWZ,NTHC

C----- INTERFACE & FREE SURFACE & BOTTOM & SIDE WALL-----C
C
      DO 1000 J=1,NTH1
          DO 1010 I=2,NR
              T(I,J,1)=-T(I,J,2)-1.0D0
 1010     CONTINUE
          DO 1030 I=2,NR
              T(I,J,NZ1)=-T(I,J,NZ)+1.0D0
 1030     CONTINUE
          DO 1040 K=1,NZ1
              T(NR1,J,K)=T(NR,J,K)
 1040     CONTINUE
 1000 CONTINUE
      CALL CENTER(T)
      DO 2000 I=1,NR1
      DO 2000 K=1,NZ1
          T(I,NTH1,K)=T(I,2,K)
          T(I, 1 ,K)=T(I,NTH,K)
          T(I,NTH1+1,K)=T(I,3,K)
          T(I,0,K)=T(I,NTH-1,K)
 2000 CONTINUE
      RETURN
      END
C******************************************************************C
      SUBROUTINE CENTER(TT)
C******************************************************************C
      IMPLICIT DOUBLE PRECISION(A-H,O-Z)
      PARAMETER(IP=51,JP=81,KP=70)
      COMMON/NODE/ NR,NR1,NTH,NTH1,NX,NX1,NY,NY1,NYC,NZ,NZ1,NWR,NWZ,NTHC
      DIMENSION TT(IP,0:JP+1,KP),S(IP,KP)
C
C ここは赤松さん
      DO 1002 K=1,NZ1
          DO 1000 J=1,NTHC
              TT(1,J,K)=TT(2,J+NTHC-1,K)
 1000     CONTINUE
          DO 1001 J=NTHC+1,NTH1
              TT(1,J,K)=TT(2,J-NTHC+1,K)
```

```
 1001       CONTINUE
 1002 CONTINUE
      RETURN
      END
C
C******************************************************************C
      SUBROUTINE UCNTER(TT)
C******************************************************************C
      IMPLICIT DOUBLE PRECISION(A-H,O-Z)
      PARAMETER(IP=51,JP=81,KP=70)
      COMMON/VELOC/U(IP,0:JP+1,KP),    V(IP,0:JP+1,KP),    W(IP,0:JP+1,KP)
     &              ,U2(IP,0:JP+1,KP) ,V2(IP,0:JP+1,KP) ,W2(IP,0:JP+1,KP)
     &              ,U3(IP,0:JP+1,KP) ,V3(IP,0:JP+1,KP) ,W3(IP,0:JP+1,KP)
     &              ,V4(IP,0:JP+1,KP)
      COMMON/TEMP/     T(IP,0:JP+1,KP),TN(IP,0:JP+1,KP),TCO(IP,0:JP+1,KP)
      COMMON/NODE/ NR,NR1,NTH,NTH1,NX,NX1,NY,NY1,NYC,NZ,NZ1,NWR,NWZ,NTHC
C
      DIMENSION VV(JP,KP),MM(JP*2),TT(IP,0:JP+1,KP)
C
      DO 1500 K=1,NZ1
         DO 1000 J=2,NTH
            VV(J,K)=0.5D0*(V(2,J-1,K)+V(2,J,K))
 1000    CONTINUE
      VV(NTH1,K)=VV( 2,K)
      VV( 1 ,K)=VV(NTH,K)
 1500 CONTINUE
C
      DO 2000 J=2,NTH
         MM(J)=J
         MM(NTH+J-1)=J
 2000 CONTINUE
C
      JOQ=(NTH-1)/4
      JTQ=(NTH-1)/4*3
C
      DO 3000 K=1,NZ1
         DO 3100 J=2,NTH
            TT(1,J,K)=(VV(MM(J+JTQ),K)-VV(MM(J+JOQ),K))*0.5D0
 3100    CONTINUE
         TT(1,NTH1,K)=TT(1, 2,K)
         TT(1, 1 ,K)=TT(1,NTH,K)
         TT(1,NTH1+1,K)=TT(1, 3,K)
         TT(1, 0 ,K)=TT(1,NTH-1,K)
 3000 CONTINUE
      RETURN
      END
C******************************************************************C
      SUBROUTINE VCNTER(TT)
C******************************************************************C
      IMPLICIT DOUBLE PRECISION(A-H,O-Z)
      PARAMETER(IP=51,JP=81,KP=70)
      COMMON/NODE/ NR,NR1,NTH,NTH1,NX,NX1,NY,NY1,NYC,NZ,NZ1,NWR,NWZ,NTHC
      DIMENSION TT(IP,0:JP+1,KP)
C
      DO 1002 K=1,NZ1
         DO 1000 J=1,NTHC
            TT(1,J,K)=-TT(2,J+NTHC-1,K)
 1000    CONTINUE
```

```
            DO 1001 J=NTHC+1,NTH1
               TT(1,J,K)=-TT(2,J-NTHC+1,K)
 1001       CONTINUE
 1002 CONTINUE
      RETURN
      END
C
c*****************************************************************
      SUBROUTINE JIKARYOKU
c*****************************************************************
      IMPLICIT DOUBLE PRECISION(A-H,O-Z)
      PARAMETER(IP=51,JP=81,KP=70)
      INTEGER ICYL
      COMMON/NODE/ NR,NR1,NTH,NTH1,NX,NX1,NY,NY1,NYC,NZ,NZ1,NWR,NWZ,NTHC
      COMMON/MGDSTY/    BX(IP-1,JP-1,KP-1), BY(IP-1,JP-1,KP-1),
     &                  BZ(IP-1,JP-1,KP-1),             BR(IP-1,JP-1,KP-1),
     &                  BTH(IP-1,JP-1,KP-1),
     &                  PRM,ALPHAB,BX0,BY0,BZ0,
     &                  G(IP,JP,KP)
      COMMON/JIKA/ GANMA
*-----------------------------------------------------------------
      DO 320 K=1,NZ1
      DO 320 J=1,NTH
      DO 320 I=1,NR1
      READ(102,20) BR(I,J,K),BTH(I,J,K),BZ(I,J,K)
   20 FORMAT(3D15.7)
  320 CONTINUE
      RETURN
      END
C
c*****************************************************************
      SUBROUTINE DATAVIEW
c*****************************************************************
      IMPLICIT DOUBLE PRECISION(A-H,O-Z)
      PARAMETER(IP=51,JP=81,KP=70)
      INTEGER ICYL
      COMMON/NAM/          NAME(10)
      COMMON/RGRID/      R(IP),RD(IP),DR,DRC(IP),HR
      COMMON/THGRID/    TH(JP),THD(JP),DTH,DTHC(JP),HTH
      COMMON/XGRID/      X(IP),XD(IP),DX,DXC(IP),HX
      COMMON/YGRID/      Y(JP),YD(JP),DY,DYC(JP),HY
      COMMON/ZGRID/      Z(KP),ZD(KP),DZ,DZC(KP),HZ
      COMMON/NODE/ NR,NR1,NTH,NTH1,NX,NX1,NY,NY1,NYC,NZ,NZ1,NWR,NWZ,NTHC
      COMMON/TEMP/      T(IP,0:JP+1,KP),TN(IP,0:JP+1,KP),TCO(IP,0:JP+1,KP)
      COMMON/VELOC/U(IP,0:JP+1,KP),    V(IP,0:JP+1,KP),    W(IP,0:JP+1,KP)
     &            ,U2(IP,0:JP+1,KP) ,V2(IP,0:JP+1,KP) ,W2(IP,0:JP+1,KP)
     &            ,U3(IP,0:JP+1,KP) ,V3(IP,0:JP+1,KP) ,W3(IP,0:JP+1,KP)
     &            ,V4(IP,0:JP+1,KP)
      COMMON/VELOCN/    UN(IP,0:JP+1,KP),VN(IP,0:JP+1,KP),WN(IP,0:JP+1,KP)
C
      COMMON/MGDSTY/    BX(IP-1,JP-1,KP-1), BY(IP-1,JP-1,KP-1),
     &                  BZ(IP-1,JP-1,KP-1),             BR(IP-1,JP-1,KP-1),
     &                  BTH(IP-1,JP-1,KP-1),
     &                  PRM,ALPHAB,BX0,BY0,BZ0,
     &                  G(IP,JP,KP)
      COMMON/PRESS/      P(IP,0:JP+1,KP)
      COMMON/DIMLSS/    RA,PR,RER,REC,GRA
      COMMON/MAPMT/      IDX,MAXIT
```

```
      COMMON/TIME/      DT
      COMMON/JIKA/ GANMA
      COMMON/PAR2/      ICYL,ITER
C
      DIMENSION DTDZF(IP,JP),DTDZB(IP,JP),
     &            TF(IP,0:JP+1,KP),TB(IP,0:JP+1,KP)
C
C--------- DATAview      109 -------------------------------------------
      WRITE(109,*)' U(I,J,K)'
      WRITE(109,100) ((U(I,3,K),I=1,NR1),K=1,NZ1)
  100 FORMAT(32D12.4)
C
      WRITE(109,*)' U3(I,J,K)'
      WRITE(109,100) ((U(I,3,K),I=1,NR1),K=1,NZ1)
c   150 FORMAT(34D12.4)
C
      WRITE(109,*)' V(I,J,K)'
      WRITE(109,100) ((V(I,3,K),I=1,NR1),K=1,NZ1)
c   200 FORMAT(34D12.4)
C
      WRITE(109,*)' V3(I,J,K)'
      WRITE(109,100) ((V(I,3,K),I=1,NR1),K=1,NZ1)
c   250 FORMAT(34D12.4)
C
      WRITE(109,*)' W(I,J,K)'
      WRITE(109,100) ((V(I,3,K),I=1,NR1),K=1,NZ1)
c   300 FORMAT(34D12.4)
C
      WRITE(109,*)' W3(I,J,K)'
      WRITE(109,100) ((V(I,3,K),I=1,NR1),K=1,NZ1)
c   350 FORMAT(34D12.4)
C
      WRITE(109,*)' T(I,J,K)'
      WRITE(109,100) ((V(I,3,K),I=1,NR1),K=1,NZ1)
c   400 FORMAT(34D12.4)
C
      WRITE(109,*)' TB(I,J,K)'
      WRITE(109,100) ((TB(I,3,K),I=1,NR1),K=1,NZ1)
c   500 FORMAT(34D12.4)
C
      WRITE(109,*)' TF(I,J,K)'
      WRITE(109,100) ((TF(I,3,K),I=1,NR1),K=1,NZ1)
c   600 FORMAT(34D12.4)
C
      RETURN
      END
```
