# Supplementary material for: Magnetothermal Convection of Water with the Presence or Absence of a Magnetic Force Acting on the Susceptibility Gradient
Source: PLoS One. 2016 Sep 8;11(9):e0160090. doi: 10.1371/journal.pone.0160090 (PMC5015909; doi:10.1371/journal.pone.0160090)
Supplement: S1 File — (ZIP) [file pone.0160090.s001.zip › S1 File Maki/S1 file.doc]

**Supporting Information Captions:**

Now I open two types of program codes to carry out the three-dimensional computation of magnetothermal convection. Both of them are written with FORTRAN. One is to compute the convection with the magnetic force acting on the susceptibility gradient *f*_sc_ and the magnetic field gradient *f*_b_ (code145_fsc_and_fb). The other is to compute the convection with magnetic force acting on the *f*_b_ only (code147_fb_only). The output data is necessary to visualize with the software Tecplot (http://www.tecplot.com/).

The code requires one text file to control over the computation (ma2pmt), one data file of the magnetic field (majibaout), ten text files to record the output (maouta, maoutb maoutc maoutd, maout, mateca, matecb, matecc, matecd, and matec), and three text files (mainp, matrnvol, DATAview). The magnetic field data are four types (case A-D, or case E-H). When you use the magnetic field, for example, change the name of “majibaout (for case A and E)” as “majibaout” (Remove “(for case A and E)”). In the compressed files, the program code, ma2pmt, and majibaout were only attached. As regards another text files, please create empty text files before the computation.

The program code of “code145_fsc_and_fb.txt” is for the case A~D, and the other code of “code147_fb_only.txt” is for the case E~H. Both are controlled by ma2pmt.

The text files of maouta, maoutb maoutc maoutd and maout, and mateca, matecb, matecc, matecd, and matec are to record the output data. The former five texts (maouta~maout) are to record the computation results. When we continue the computation, we change the name of “maout*” to “mainp”, and restart the computation after the addition of one empty file named “maout*”. Before the restart, change the initial condition in the “ma2pmt” (number of times for computation and nondimensional time etc.).

The latter five texts (mateca ~ matec) are used for the visualization by the Tecplot. The data of “maout*” and “matec*” are corresponded each other. For example, the result of “maouta” is visualized with the data of “mateca”.

This code adopts an explicit method. Hence the time interval of computation (e.g. nondimensionalized time) is influenced by the stability of computation and the grid size. In other words, the time interval differs as the computation progresses. The time interval is also changed in the “ma2pmt”. If the computation is diverged, we make the time interval be small or the level of “EPS” in ma2pmt be loosen.

Whether the computation has converged to a stable solution or not can be detected with “matrnvol”. The “matrnvol” records the lapse of computation results.

The text of “DATAview” is unnecessary to carry out the computation.

Finally, the program code and ma2pmt contain lots of Japanese comments. But they do not influence the computation because they are regarded as the comment. All the readers who can understand FORTRAN, this program code will be comprehended easily. I hope this code can be modified and applied.
